# Soluble epoxide hydrolase inhibition restores pro-resolving lipid mediators and reduces inflammation in localized provoked vulvodynia

**DOI:** 10.3389/fphar.2026.1741914

**Published:** 2026-03-26

**Authors:** Emanuelle Chrysilla, Subin Hong, Ruth Ajiboye, Hyun Ah (Annette) Lee, Krishna Rao Maddipati, Tanzy Love, Mitchell A. Linder, Megan L. Falsetta

**Affiliations:** 1 Department of Pharmacology and Physiology, University of Rochester, Rochester, NY, United States; 2 Department of Obstetrics and Gynecology, University of Rochester, Rochester, NY, United States; 3 Lipidomics Core Facility and Bioactive Lipids Research Program, Wayne State University, Detroit, MI, United States; 4 Department of Biostatistics and Computational Biology, University of Rochester, Rochester, NY, United States

**Keywords:** chronic pain, inflammation, sEH, soluble epoxide hydrolase, specialized pro-resolving lipid mediators, vulvodynia

## Abstract

Localized provoked vulvodynia (LPV) is a chronic pain disorder characterized by persistent inflammation of the vulvar vestibule, a ring of tissue immediately surrounding the vaginal opening, with no effective, mechanism-based treatments. Recent findings suggest that LPV is associated with a deficiency in pro-resolving lipid mediators, namely, epoxyeicosatrienoic acids (EETs), that may impede the resolution of inflammation. Soluble epoxide hydrolase (sEH) is the enzyme responsible for metabolizing these EETs into less potent dihydroxyeicosatrienoic acids (DHETs). Inhibiting sEH therefore prolongs the ability of these lipids to exert their anti-inflammatory properties, making it a promising therapeutic approach to restore inflammation resolution in LPV. In this study, we examined sEH expression and activity in fibroblasts derived from vestibular and external vulvar biopsies of LPV patients and controls. siRNA knockdown of sEH in primary vestibular fibroblasts reduced pro-inflammatory mediator release following IL-1β stimulation. Pharmacological sEH inhibition reduced DHET levels, increased the EET/DHET ratio, and shifted the lipidomic profile toward a pro-resolving phenotype. Treatment with three different sEH inhibitors consistently reduced pro-inflammatory mediator production in LPV fibroblasts, including IL-6, IL-8, and PGE2. These findings demonstrate that elevated sEH activity contributes to the chronic inflammatory state in LPV, and that pharmacological sEH inhibition restores lipid mediator balance and suppresses inflammatory signaling, thus supporting a novel therapeutic strategy for LPV.

## 1 Introduction

Localized provoked vulvodynia (LPV) is a condition characterized by severe pain lasting more than 3 months localized to the vulvar vestibule, a specialized ring of tissue surrounding the vaginal opening, upon light pressure or contact (Sadownik, 2014; Bohm-Starke, 2010; Bornstein et al., 2016), while adjacent vulvar areas are non-painful to touch. Affecting an estimated 8%–16% of women of reproductive age in the United States (Reed et al., 2012; Bergeron et al., 2020), LPV presents as a significant burden due to its multifactorial etiology, underdiagnosis, and lack of standardized management strategies (Torres-Cueco and Nohales-Alfonso, 2021; Vasileva et al., 2020; Paavonen and Eschenbach, 2021). Women with LPV often endure years of misdiagnosis and ineffective interventions, leading to psychological distress, impaired sexual function, and a diminished overall quality of life (Bergeron et al., 2014; Falsetta et al., 2023). Current pharmacological treatments, including non-steroidal anti-inflammatory drugs (NSAIDs), corticosteroids, and selective serotonin reuptake inhibitors (SSRIs), only manage the symptoms of pain and fail to address the underlying pathophysiology of the disease (Falsetta et al., 2017; Goldstein et al., 2016). Therefore, new mechanistically targeted therapies are desperately needed.

Recent findings suggest that inflammation plays a key role in the initiation of the condition (Falsetta et al., 2015; Foster et al., 2015; Bekauri et al., 2024; Akopians and Rapkin, 2015). Approximately 70% of LPV patients have a history of recurrent yeast infections (Donders and Bellen, 2012; Farmer et al., 2011), compared to 5%–8% of healthy women (Foxman et al., 1998), which is defined as four or more yeast infections annually (Sobel, 2007). Because the vulvar vestibule is non-keratinized (Kim et al., 2024; Farmer, 2015), it displays an increased inflammatory response compared to adjacent vulvar tissues to facilitate the rapid detection of pathogens. In LPV patients, this inflammatory response is exaggerated to the point that it is deleterious, which, when coupled with a defect in the resolution machinery, culminates in pain (Bekauri et al., 2024; Falsetta et al., 2015; Falsetta et al., 2023). The downregulation of anti-inflammatory lipid mediators known as epoxyeicosatrienoic acids (EETs) in tissue biopsies obtained from the painful vestibule LPV patients is evidence of a defect in resolution (Bekauri et al., 2024). These endogenous signaling molecules, derived from arachidonic acid (AA) via the action of cytochrome P450 (CYP450) epoxygenases, play a pivotal role in promoting tissue repair, modulating nociception, and reducing pro-inflammatory cytokine production (Shi et al., 2022; Liu et al., 2021; Thomson et al., 2012).

The biological availability of EETs is tightly regulated by enzyme soluble epoxide hydrolase (sEH), which rapidly metabolizes EETs into less active, and often pro-inflammatory, dihydroxyeicosatrienoic acids (DHETs) (Harris and Hammock, 2013). Upregulation of sEH activity has been implicated in the attenuation of EETs in several chronic inflammatory disorders, including cardiovascular disease, arthritis, and neuropathic pain (Hammock et al., 2021; Luther et al., 2021; Martini et al., 2022). Given the emerging evidence that LPV involves chronic inflammation driven in part by diminished EET bioavailability (Bekauri et al., 2024), pharmacological inhibition of sEH offers a novel therapeutic strategy to treat LPV by restoring EET-mediated resolution, alleviating inflammation and its associated pain.

Therefore, in this study, we explored the therapeutic potential of sEH inhibitors as a mechanism-based intervention for vulvodynia using an *in vitro* model of LPV. Increased sEH activity was observed in LPV vestibular fibroblasts compared to controls. Under inflammatory conditions, knockdown of sEH in LPV fibroblasts caused a significant decrease in pro-inflammatory mediator production, thereby implicating sEH as a key contributor to LPV inflammation. Metabololipidomics data showed a significant increase in EET/DHET ratio upon sEH inhibitor treatment compared to baseline. In addition, inhibiting sEH consistently suppressed multiple pro-inflammatory cytokines and chemokines. Overall, these findings suggest that pharmacological sEH inhibition could serve as a novel therapy for LPV due to its ability to attenuate inflammation and reduce LPV-associated vulvar pain.

## 2 Methods

### 2.1 Patient/Sample selection

LPV cases (fulfilling Friedrich’s Criteria (Bergeron et al., 2001)) and pain-free controls were recruited from the Division of General Obstetrics and Gynecology clinical practice at the University of Rochester between December 2012 and January 2025. All subjects provided informed consent, and the research was approved by the University of Rochester Institutional Review Board (RSRB # 42136). Expanded details on our selection criteria and sampling procedures have been previously published (Foster et al., 2015; Falsetta et al., 2016). In brief, cases and controls were age- and race-matched with a mean age of 33.5 years for both groups. A decision to match by age (within 3 years) was based on reports of increased IL-6 production by fibroblasts from older donors (Wolf et al., 2012). All case and control subjects were Caucasian, non-Hispanic. Furthermore, all subjects denied the use of corticosteroids and non-steroidal anti-inflammatory medications and had no chronic inflammatory illnesses other than LPV. All subjects did not have active yeast infections at time of sampling. Prior to biopsy of the vestibular and external vulvar sites, sampling sites underwent mechanical Wagner™ algometry. We used a method-of-limits technique for measuring vulvodynia mechanical pain threshold initially described by Zolnoun et al. (2012) and replicated in an earlier publication (Foster et al., 2015; Falsetta et al., 2016). 6 mm biopsies from the vestibular and external vulvar sites were then obtained and processed for fibroblast strain development as previously described (Foster et al., 2015). A total of 10 case and 8 control patient-derived fibroblast strains were used in this study (Supplementary Table S1).

### 2.2 Fibroblast culture and experimental treatments

Primary fibroblast strains were cultured in Minimum Essential Medium (MEM) supplemented with 10% fetal bovine serum (FBS), GlutaMAX, gentamycin, and antibiotic/antimycotic solution (Thermo Fisher, Waltham, MA). Early passage (4–10) external vulvar and vestibular fibroblast strains were seeded into 6-well, 12-well, or 96-well tissue culture plates at 2.5 × 10^4^ cells/cm^2^. Once confluent, cells were collected and used for gene expression, protein expression, and enzymatic activity assays. For experimental treatments, cells were transitioned to serum-free MEM or serum-free, phenol-free MEM for 4–24 h to reduce background inflammation. The following treatments were prepared in serum-free MEM or serum-free, phenol-free MEM and applied to cultures for 48 h prior to subsequent experimentation: vehicle [dimethyl sulfoxide (DMSO)], recombinant human interleukin 1-beta (rhIL-1β) [500 pg/mL] (Invitrogen, Waltham, MA, A42507), N-[1-(1-oxopropyl)-4-piperidinyl]-N′-[4-(trifluoromethoxy)phenyl)-urea (TPPU) [10 nM] (Cayman Chemical Company, Ann Arbor, MI), GSK2256294 [250 pM] (MedChemExpress, Monmouth Junction, NJ), and EC5026 [5 nM] (MedChemExpress). The doses described above were chosen based on each inhibitor’s predicted concentration at which it will inhibit 90% of the enzyme (IC_90_), calculated using GraphPad (https://www.graphpad.com/quickcalcs/ecanything1/), based upon each inhibitor’s known IC_50_ and a Hill Slope of 1. All experiments were conducted using 6 or 12 biological replicates, consisting of paired vestibular and external vulvar fibroblast strains derived from LPV patients and matched healthy controls. Each strain was assayed in technical triplicate (unless otherwise stated in the figure legend) per treatment condition.

### 2.3 RNA extraction, cDNA synthesis, and reverse transcriptase quantitative PCR

Confluent primary fibroblast strains were lysed using Qiazol Lysis Reagent (Qiagen, Carlsbad, CA). Total RNA was isolated from fibroblasts using the RNeasy Mini Kit (Qiagen), following the manufacturer’s instructions. RNA was quantified by spectrophotometric analysis using a NanoDrop One spectrophotometer (Thermo Fisher). RNA was reverse transcribed into cDNA using the iScript cDNA Synthesis Kit (BioRad, Hercules, CA). Each reaction contained 300 ng of RNA template diluted in 15 μL of RNase-free molecular grade water (Qiagen), 1 μL of iScript reverse transcriptase, and 4 μL of 5x iScript reaction mix buffer. Negative reverse transcriptase controls that contained no iScript reverse transcriptase were also prepared to confirm the absence of DNA contamination. After amplification, cDNA samples were diluted 5-fold in RNase-free molecular grade water (Qiagen) and used as templates for reverse transcriptase quantitative PCR (RT-qPCR) reactions (5 μL/reaction). sEH mRNA expression was evaluated using the following sequence: EPHX2, F: 5′-TGCCATCCTCACCAACAC-3′/R: 5′-ACG​GAC​CCT​GGG​CTT​TAC-3′ and using 18S rRNA as the reference gene.

### 2.4 Western blotting

Fibroblasts were manually scraped off culture plates, collected in 1X Cell Lysis Buffer (Cell Signaling Technology, Danvers, MA), and homogenized by sonication. Protein concentrations were determined using a DC Protein Assay (Bio-Rad) with bovine serum albumin (BSA) as the standard. Samples (25 µg per lane) were diluted in 4X Laemmli Buffer (Bio-Rad), heated at 95 °C for 10 min, and separated by SDS-PAGE on a 10% polyacrylamide gel. Protein gels were imaged and crosslinked using a ChemiDoc MP Imaging System (Bio-Rad) and then transferred to PVDF membranes using a Trans-Blot Turbo Transfer System (Bio-Rad). Protein blots were probed with EPHX2 rabbit polyclonal antibody (Abclonal, Woburn, MA, Cat: A1885) at a dilution of 1:1,000, hFAB Rhodamine Anti-Tubulin Primary Antibody (Bio-Rad, Cat: #12004166) at a dilution of 1:5,000, and peroxidase AffiniPure goat anti-rabbit IgG (H + L) (Jackson ImmunoResearch, West Grove, PA, Cat: 111-035-003) at a dilution of 1:4,000. Protein bands were visualized using Clarity Western ECL Substrate (Bio-Rad). Bands were normalized to β-tubulin using Image Lab software version 6.1 (Bio-Rad).

### 2.5 sEH activity assay

sEH activity was assessed using a soluble epoxide hydrolase activity assay kit following the manufacturer’s instructions (Cayman Chemical Company, Cat: 600090). After treatment, cells were lysed using a digitonin-containing lysis buffer to release intracellular sEH. 100 μL of Epoxy Fluor 7 substrate reaction mix, which is hydrolyzed by sEH to a highly fluorescent product, was added to each well, and the plates were read on a FlexStation 3 microplate reader (Molecular Devices, San Jose, CA). Fluorescence was monitored kinetically at 30-s intervals for 30 min, using excitation and emission wavelengths of 330 nm and 465 nm, respectively. Standard curves were generated using linear regression, and the sEH activity of each sample was determined using the following equation:

sEH activity pmol/⁡min⁡/mL=Sample Fluorescence‐y interceptslope×dilution factor30 minutes

### 2.6 Small-interfering (siRNA) knockdown

An siRNA against human EPHX2 (Silencer Select, Thermo Fisher, Cat: AM16708) was transfected into fibroblasts from three LPV cases using Lipofectamine 3000 (Thermo Fisher). Cells were then treated with vehicle (ethanol) or IL-1β [500 pg/mL] (Cayman Chemical Company) for 48 h, and IL-6 and PGE_2_ levels were measured. Knockdown of sEH was confirmed by Western blotting.

### 2.7 Enzyme-linked immunosorbent assay (ELISA)

Cell supernatants were collected 48 h post-treatment and stored at 4 °C until molecular analysis. Standard sandwich ELISAs were used to measure the production of IL-6 and IL-8 (BD Biosciences, Franklin Lakes, NJ), and competitive EIAs were used to measure the production of PGE2 (Cayman Chemical Company, 500141).

### 2.8 Lipidomic analysis

Fibroblasts were manually scraped from the culture plates, collected in 3 mL culture tubes, and homogenized using a probe sonicator. 0.1% butylated hydroxytoluene (BHT, Sigma Aldrich, St. Louis, MO) was added to each sample to stabilize lipids. Samples were then stored at −80 °C until analysis. Quantitative targeted lipidomic analysis was performed by the Wayne State University Lipidomics Core using liquid chromatography mass spectrometry (LC-MS). Briefly, samples were prepared using C18 reverse phase cartridges (Phenomenex, StrataX SPE cartridge, 30 mg sorbent), and then subjected to reverse phase HPLC on a C18 column (Luna, C18, 3 μm, 2 mm × 150 mm, Phenomenex, CA) as published earlier (Maddipati et al., 2016; Maddipati et al., 2014).

### 2.9 17-plex luminex assay

Cell supernatants were collected 48 h post-treatment, stabilized with 0.5% BSA (Thermo Fisher), and stored at −80 °C until molecular analysis. The levels of 17 different cytokines were measured using a Human Cytokine 17-Plex Assay Kit (Bio-Rad, Cat: M5000031YV) following the manufacturer’s instructions. Briefly, 50 µL of supernatants or standards were added to the wells containing the capture antibody-immobilized beads and incubated for 30 min at room temperature on a plate shaker. After washing, 25 µL of biotinylated detection antibody was added, followed by incubation for 30 min. The beads were washed again, and 50 µL of streptavidin-phycoerythrin (SAPE) was added for 10 min before a final wash step. The plate was then read on a Luminex 200 instrument (Luminex Corporation, Austin, TX) controlled by xPonent® 3.1 software. Standard curves were generated using a five-parameter logistic curve fit, and analyte concentrations were determined using Bio-Plex Manager software (Bio-Rad).

### 2.10 Calcium imaging

After treatment, cells were loaded with 4 µM Fura-2/AM (Invitrogen) in MEM and incubated at 37 °C in the dark for 1 h. The cells were subsequently washed three times with calcium imaging buffer (10 mM HEPES, 1.26 mM CaCl_2_, 137 mM NaCl, 4.7 mM KCl, 5.5 mM glucose, 1 mM Na_2_HPO_4_, and 0.56 mM MgCl_2_, at pH 7.4) before assessing calcium influx on a FlexStation 3 microplate reader (Molecular Devices) by alternately exciting Fura-2/AM at 340 and 380 nm and collecting emission fluorescence at 510 nm. After recording the baseline fluorescence for 30 s, one of the following TRPV4 agonists: GSK1016790A [1 µM] (Cayman Chemical Company), 5,6-EET [1 µM] (Cayman Chemical Company), 8,9-EET [1 µM] (Cayman Chemical Company), 11,12-EET [1 µM] (Cayman Chemical Company), or 14,15-EET [1 µM] (Cayman Chemical Company), were added to the cells through a multi-channel pipettor included as a part of the fluidics module of the FlexStation 3. The fluorescence changes were monitored for an additional 180 s. The relative Fura-2/AM fluorescence changes for each well were expressed as ΔF = (F − F_0_), where F and F_0_ are fluorescence values at the time of interest and the beginning of the reading, respectively.

### 2.11 Statistical analysis

For each comparison of mRNA/protein expression, enzyme activity, cytokine abundance, and calcium flux, statistical comparisons were performed using one-way or two-way analysis of variance (ANOVA) followed by Tukey’s *post hoc* test for multiple group comparisons. Normality and variance were assessed prior to statistical testing. A linear mixed-effects model was fit for our lipidomics analysis. The technical replicates for each subject were random effects, whereas the LPV status (case/control), location (vest/vulv), and treatment were fixed effects. We tested for differences between the treatment groups and for location and LPV status differences within each treatment group. First, main effects for treatment differences were tested, then the pairwise contrasts between the four LPV status and location combinations were tested, and the significance of these is denoted in the figures. Differences were reported as significant if the p-value for the difference was less than 0.05. “ns” denotes comparisons that did not reach significance. Analyses and visualizations of the data were performed using GraphPad Prism (version 10.4.1) software. Each data point shown in the figures represents one biological replicate, and each biological replicate was assayed in technical triplicate (unless otherwise stated in the figure legend), with replicate values averaged and presented as mean ± standard error of the mean (SEM).

## 3 Results

### 3.1 sEH contributes to inflammatory signaling in LPV

Fibroblasts isolated from the vestibular region of LPV patients have been shown to produce significantly higher levels of pro-inflammatory mediators such as interleukin-6 (IL-6) and prostaglandin E2 (PGE2) compared to fibroblasts derived from non-painful external vulvar sites or from healthy controls (Falsetta et al., 2021; Falsetta et al., 2016). Elevated production of these mediators correlates with increased pain sensitivity in LPV cases (Foster et al., 2015). Since fibroblasts play a key role in the immune response, retain their phenotypes *in vitro*, and produce pro-inflammatory mediators (Foster et al., 2015; Falsetta et al., 2018; Van Linthout et al., 2014), they represent a highly relevant cellular model for studying LPV.

**FIGURE 1:**
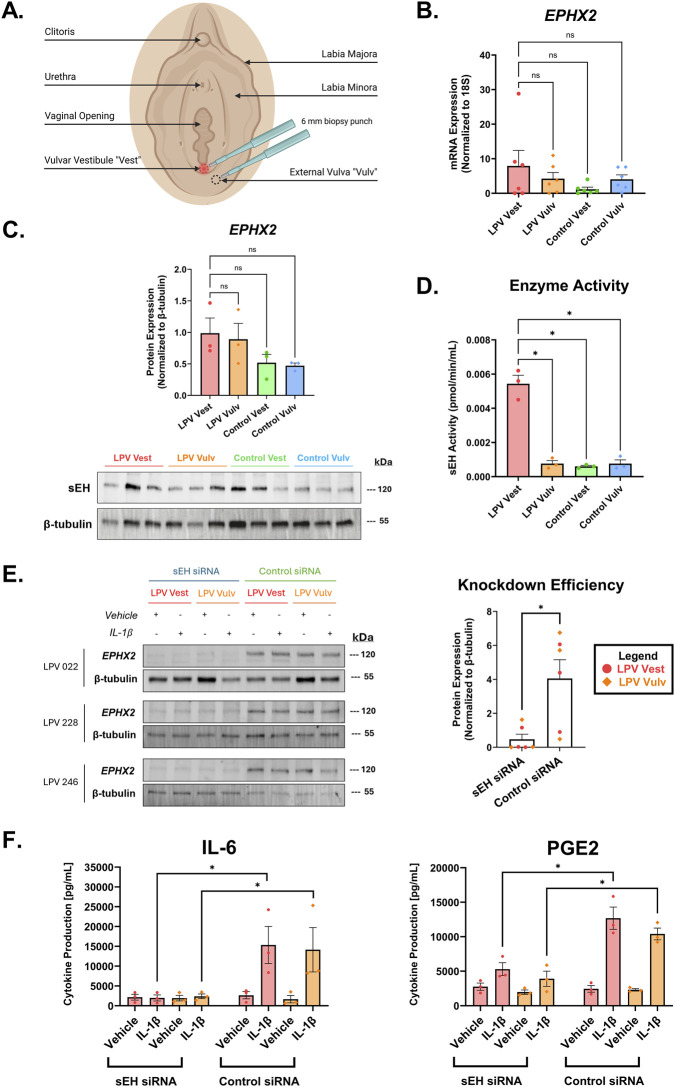
sEH is involved in the LPV inflammatory process. **(A)** Illustration of punch biopsy collection from LPV cases and healthy controls during surgery. Light touch to the vulvar vestibule (Vest) is immensely painful, whereas the adjacent external vulva (Vulv) is not painful to touch. **(B)** RT-qPCR data showing sEH (EPHX2) mRNA expression, normalized to 18S rRNA, in LPV and control fibroblasts (n = 6 cases and 6 controls). **(C)** Western blot data showing sEH (EPHX2) protein expression, normalized to β-tubulin, in LPV and control fibroblasts (n = 3 cases and 3 controls). **(D)** sEH enzyme activity is significantly elevated in the LPV vest compared to LPV vulv, control vest, and control vulv (n = 3 cases and 3 controls). The data was collected in technical triplicates and are represented as mean ± SEM, one-way ANOVA + Tukey’s *post hoc* test, *P < 0.05, ns = not significant. **(E)** siRNA knockdown with sEH siRNA [5 µg] in vulvar and vestibular fibroblasts derived from 3 LPV cases was confirmed through Western blotting. **(F)** Transfection of sEH siRNA in LPV case vestibular and vulvar fibroblasts, but not control siRNA, reduced IL-6 and PGE2 production upon IL-1β [500 pg/mL] stimulation (n = 3 cases). The data was collected in six technical replicates and are represented as mean ± SEM, two-way ANOVA + Tukey’s *post hoc* test, *P < 0.05.

To examine the expression of sEH between painful and non-painful areas of the vulva, 6 mm punch biopsies were collected from both the vestibular (vest) and external vulvar (vulv) regions of LPV cases and controls (Figure 1A). These punch biopsies were then used to establish primary human vestibular and vulvar fibroblast strains. RT-qPCR analysis of fibroblasts from these sites revealed a trend toward increased sEH (EPHX2) mRNA expression in vestibular fibroblasts from LPV patients compared to control subjects, and to their own external vulvar fibroblasts, although this difference did not reach statistical significance (Figure 1B). Similarly, Western blot analysis demonstrated a non-significant but upward trend in sEH protein levels in LPV vestibular fibroblasts relative to controls (Figure 1C). In contrast, enzymatic activity assays of cell lysates from the same anatomical sites showed a statistically significant elevation in sEH activity specifically in the vestibular region of LPV cases (P < 0.05; Figure 1D). This indicates that despite moderate expression changes at the mRNA and protein levels, sEH is functionally more active in the affected site of LPV cases, thus supporting a possible role of the enzyme in LPV pathophysiology.

To determine whether sEH is involved in the pathogenesis of LPV, fibroblasts derived from three LPV cases were transfected with 5 µg of anti-sEH siRNA or negative control siRNA for 48 h before stimulating them with vehicle or 500 pg/mL of the inflammatory instigator interleukin 1-beta (IL-1β) for another 48 h. Because inflammation plays a key role in the LPV disease mechanism (Falsetta et al., 2017; Akopians and Rapkin, 2015; Falsetta et al., 2015; Falsetta et al., 2016; Falsetta et al., 2018; Baker et al., 2016), the addition of IL-1β was used to induce pro-inflammatory mediator production. Western blot analysis confirmed that transfection of the negative control siRNA did not affect sEH protein expression in LPV fibroblasts (Figure 1E). However, transfection of the anti-sEH siRNA ablated sEH expression (Figure 1E). In addition, knocking down sEH *in vitro* led to a reduction in pro-inflammatory cytokine production. LPV case vest and vulv fibroblasts transfected with anti-sEH siRNA showed decreased IL-6 and PGE2 production upon 500 pg/mL IL-1β stimulation compared to cells transfected with negative control siRNA (Figure 1F). Treatment with vehicle (DMSO) did not affect IL-6 or PGE2 production upon transfection with negative control or anti-sEH siRNA. Taken together, these findings suggest that sEH directly contributes to the LPV inflammatory signaling cascade, thus making it a promising target for a mechanism-based therapy.

### 3.2 Effects of sEH inhibition on lipid mediator profiles of primary vestibular fibroblasts

Although sEH inhibitors have not been tested as an LPV therapy, there is a wealth of preclinical data and even early results from clinical trials to suggest that they have the capacity to reduce pain and inflammation in other diseases (Trindade-da-Silva et al., 2020; Napimoga et al., 2018; Abdalla et al., 2023; Ballassini Abdalla et al., 2022). The sEH inhibitor 1-Trifluoromethoxyphenyl-3-(1-propionylpiperidin-4-yl) urea (TPPU) has become the preferred sEH inhibitor in biological research due to its favorable pharmacokinetic profile and high potency against both human and rodent sEH enzymes, with IC_50_ values of 3.7 nM and 2.8 nM, respectively (Lee et al., 2014; Huang, 2023). Therefore, we elected to use TPPU to begin our preclinical studies on inhibiting sEH as a potential therapy for LPV.

**FIGURE 2:**
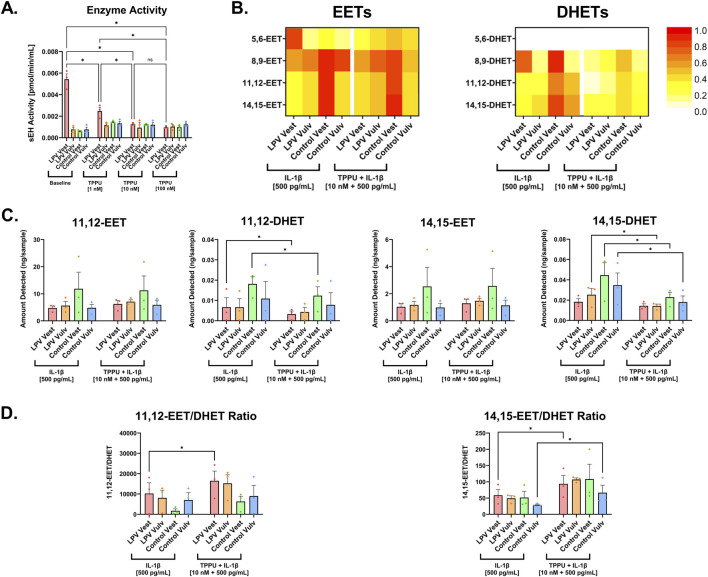
Pharmacological sEH inhibition using TPPU significantly reduced DHET formation and increased EET/DHET ratio. **(A)** sEH activity was quantified in LPV and control fibroblasts treated without TPPU (Baseline), with 1 nM, 10 nM, or 100 nM TPPU. **(B)** Heat map analyses showing 10 nM TPPU treatment maintained the levels of anti-inflammatory EETs and reduced the levels of pro-inflammatory DHETs. For a given lipid, all values were scaled relative to the highest observed quantity of that lipid within the dataset, which was set to 1 (or 100%). Heatmap colors represent relative changes within each lipid across treatments. **(C)** Individual bar graphs of 11,12-EET, 11,12-DHET, 14,15-EET, and 14,15-DHET. **(D)** Increased EET/DHET ratios in the painful vestibule of LPV patients upon IL-1β + 10 nM TPPU treatment compared to IL-1β treatment alone. The data was collected in technical triplicates and are represented as mean ± SEM, n = 3 cases and 3 controls, *P < 0.05, ns = not significant.

To determine the optimum dose of TPPU, 3 LPV case and 3 control fibroblast strains were treated with varying doses of TPPU (1 nM, 10 nM, and 100 nM). Dose response analysis was done using a fluorescence-based sEH activity assay to confirm that these predicted doses were effective at reducing sEH activity. Enzyme activity assays revealed that TPPU significantly inhibited sEH activity in a dose-dependent manner (Figure 2A). 1 nM, 10 nM, and 100 nM TPPU reduced sEH activity in LPV vestibular fibroblasts compared to baseline (P < 0.05). Both 10 nM and 100 nM TPPU significantly reduced sEH activity compared to 1 nM TPPU (P < 0.05), but no significant difference was observed between the 10 nM and 100 nM doses. Based on these results, the 10 nM dose was selected for subsequent experimentation.

To characterize changes in vulvar fibroblast lipid mediator profiles following sEH inhibition, a lipidomic analysis was performed on cells treated with a combination of IL-1β and 10 nM TPPU, and a comparison was made on these profiles to those from IL-1β-treated cells. Lipid profiling via LC-MS detected 122 unique lipid mediators in vulvar fibroblasts, including metabolites derived from cyclooxygenase (COX), lipoxygenase (LOX), and CYP450 pathways (Supplementary Table S2; Supplementary Figure S1). CYP450 epoxygenases produce four EET regioisomers from AA: 5,6-EET, 8,9-EET, 11,12-EET, and 14,15-EET. Heatmap analysis showed that TPPU treatment resulted in the preservation of these EETs and a corresponding decrease in their less active DHET metabolites compared to IL-1β treatment alone (Figure 2B). It is known that 11,12-EET and 14,15-EET are the two predominant EETs produced by many different cell types and tissues (Spector et al., 2004). Our data confirmed their abundance, with control vestibular fibroblast strains producing upwards of 20 ng and 5 ng of 11,12-EET and 14,15-EET, respectively (Figure 2C), compared to less than 0.1 ng of 5,6-EET and 8,9-EET produced (Supplementary Figure S1). Therefore, subsequent analysis highlighted these two EETs as they represent the most abundant EET species. Consistent with previously reported findings (Bekauri et al., 2024), LPV vestibular fibroblasts produced significantly lower levels of 11,12-EET and 14,15-EET compared to matched controls (Figure 2C). Although a significant elevation in EETs was not observed, individual lipid plots confirmed the significant reduction of 11,12-DHET and 14,15-DHET upon TPPU treatment (P < 0.05; Figure 2C). These shifts resulted in significantly elevated EET/DHET ratios in TPPU-treated LPV vestibular fibroblasts compared to those treated with IL-1β alone (P < 0.05; Figure 2D). These results demonstrate that TPPU effectively increased EET/DHET ratios in LPV vestibular fibroblasts, altering the local lipid mediator environment in favor of the resolution of inflammation.

**FIGURE 3:**
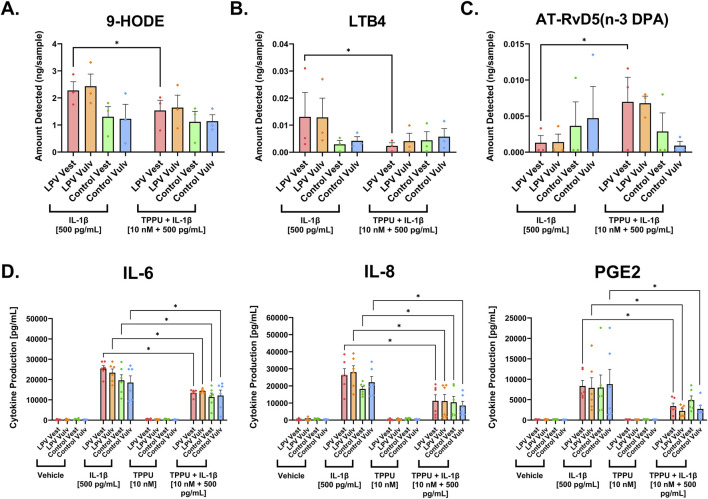
Effect of TPPU on inflammation and resolution in vulvodynia. Individual bar graphs of **(A)** 9-HODE, **(B)** LTB4, and **(C)** AT-RvD5(n-3 DPA) detected in vulvar and vestibular fibroblasts upon 48-h treatment with IL-1β [500 pg/mL] or a combination of IL-1β [500 pg/mL] and TPPU [10 nM] (n = 3 cases and 3 controls). **(D)** ELISA data showing levels of IL-6, IL-8, and PGE2 upon 48-h vehicle, IL-1β [500 pg/mL], TPPU [10 nM], and combination treatments. Addition of TPPU in the presence of IL-1β significantly decreased production of IL-6, IL-8, and PGE2 on average across fibroblast strains (n = 6 cases and 6 controls). The data was collected in technical triplicates and are represented as mean ± SEM, two-way ANOVA + Tukey’s *post hoc* test, *P < 0.05.

Beyond the CYP450 pathway, metabolites of COX and LOX were also altered upon TPPU treatment (Supplementary Table S2; Supplementary Figure S1). Of note, the pro-inflammatory lipids 9-hydroxyoctadecadienoic acid (9-HODE) and leukotriene B4 (LTB4) were significantly reduced in all fibroblast strains treated with TPPU (Figures 3A,B). In contrast, there was an increase in pro-resolving mediators such as resolvin D5 derived from n-3 DPA [AT-RvD5(n-3-DPA)], specifically in LPV case vestibular strains upon pharmacological inhibition of sEH (Figure 3C). ELISA data further corroborated these findings, showing significantly reduced levels of IL-6, IL-8, and PGE2 in fibroblast strains treated with a combination of IL-1β and TPPU compared to treatment with IL-1β alone (P < 0.05; Figure 3D). Fibroblast strains treated with TPPU only (no IL-1β stimulation) had no effect on IL-6, IL-8, and PGE2 production, suggesting that the inhibitor alone had no basal effects, and the observed reductions in mediator levels reflect modulation of inflammatory signaling. Because IL-6, IL-8, and PGE2 are considered surrogate markers of inflammation in LPV (Foster et al., 2015; Falsetta et al., 2016; Falsetta et al., 2021), reducing the levels of these mediators should at least in part attenuate inflammatory signaling. Collectively, these results show that pharmacological sEH inhibition using TPPU shifts the vulvar fibroblast lipidome profile away from prostaglandin and leukotriene-driven inflammatory signaling and toward resolution pathways.

### 3.3 Clinically relevant sEH inhibitors reduce sEH activity and pro-inflammatory mediator production *in vitro*

**FIGURE 4:**
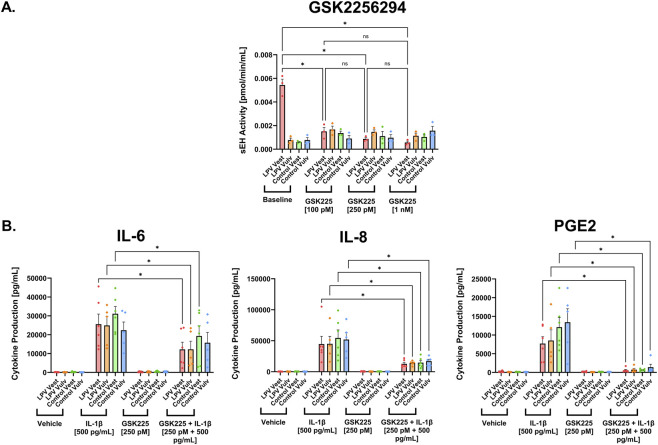
The clinically relevant sEH inhibitor GSK2256294 also reduced surrogate markers of pain in LPV fibroblasts. **(A)** sEH activity was quantified in LPV and control fibroblasts treated without GSK2256294 (Baseline), with 100 pM, 250 pM, or 1 nM GSK2256294 (n = 3 cases and 3 controls). **(B)** ELISA data showing levels of IL-6, IL-8, and PGE2 upon 48-h vehicle, IL-1β [500 pg/mL], GSK2256294 [250 pM], and combination treatments. Addition of GSK2256294 in the presence of IL-1β significantly decreased production of IL-6, IL-8, and PGE2, on average across fibroblast strains (n = 6 cases and 6 controls). The data was collected in technical triplicates and are represented as mean ± SEM, two-way ANOVA + Tukey’s *post hoc* test, *P < 0.05, ns = not significant.

**FIGURE 5:**
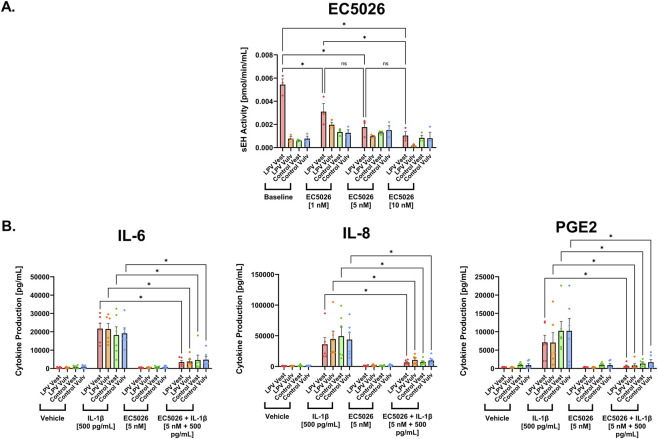
sEH inhibition using EC5026 mitigates pro-inflammatory mediator production in vulvar and vestibular fibroblasts. **(A)** sEH activity was quantified in LPV and control fibroblasts treated without EC5026 (Baseline), with 1 nM, 5 nM, or 10 nM EC5026 (n = 3 cases and 3 controls). **(B)** ELISA data showing levels of IL-6, IL-8, and PGE2 upon 48-h vehicle, IL-1β [500 pg/mL], EC5026 [5 nM], and combination treatments. Addition of EC5026 in the presence of IL-1β significantly decreased production of IL-6, IL-8, and PGE2, on average across fibroblast strains (n = 6 cases and 6 controls). The data was collected in technical triplicates and are represented as mean ± SEM, two-way ANOVA + Tukey’s *post hoc* test, *P < 0.05, ns = not significant.

Although pharmacological sEH inhibition with TPPU gave promising results in its ability to alleviate LPV-associated inflammation, it has not yet been used in clinical studies. To speed translation for vulvodynia, we screened other sEH inhibitors that are further along the drug development pipeline, specifically GSK2256294 and EC5026, both of which are currently undergoing Phase I and II clinical trials for other indications (Hammock et al., 2021; Luther et al., 2021; Martini et al., 2022). Similar to TPPU, sEH activity assays demonstrated that both inhibitors significantly suppressed enzymatic activity in LPV and control fibroblasts in a dose-dependent manner. For GSK2256294, concentrations of 250 pM and 1 nM significantly reduced sEH activity compared to cells without the inhibitor (P < 0.05), with no additional suppression observed between 250 pM and 1 nM (Figure 4A). Similarly, EC5026 at 5 nM and 10 nM significantly decreased sEH activity (P < 0.05), with no significant difference between the two highest doses (Figure 5A). Since neither the 250 pM GSK2256294 nor 5 nM EC5026 doses were toxic to cells (Supplementary Figure S2), these doses were chosen for downstream analysis.

To determine whether GSK2256294 and EC5026 also reduce pro-inflammatory cytokine production associated with pain in vulvodynia, the production of IL-6, IL-8, and PGE2 were examined in vulvar fibroblasts cultured from 6 LPV cases and 6 controls upon treatment with vehicle (DMSO), GSK2256294, or EC5026 for 48 h in the presence or absence of the inflammatory instigator IL-1β. IL-1β stimulation significantly elevated IL-6, IL-8, and PGE2 levels, confirming a robust inflammatory response in LPV and control fibroblast strains. Again, treatment with either inhibitor in the absence of IL-1β had no effect on IL-6, IL-8, and PGE2 production. Treatment with either GSK2256294 (Figure 4B) or EC5026 (Figure 5B) in the presence of IL-1β significantly attenuated IL-1β–induced production of IL-6 and IL-8 in LPV fibroblasts (P < 0.05). A modest but significant decrease in PGE2 was also observed with both inhibitors (P < 0.05). These results were consistent across all three inhibitors: TPPU, GSK2256294, and EC5026, thereby confirming that multiple sEH-targeting compounds can modulate inflammatory mediators associated with LPV pain.

### 3.4 GSK2256294 and EC5026 treatment suppress a broad range of pro-inflammatory cytokines in LPV fibroblasts

As discussed, there is a heightened response to inflammatory stimuli in the vulvar vestibule of LPV patients, leading to vestibular fibroblasts producing increased levels of IL-6 and PGE2 which are directly correlated with their pain thresholds (Falsetta et al., 2021; Foster et al., 2015; Falsetta et al., 2016). Therefore, in our *in vitro* studies thus far, upon treatment with candidate sEH inhibitors, we used reductions in IL-6, IL-8, and PGE2 as indicators of therapeutic potential. Because inflammation is a complex process involving a wide array of signaling molecules, namely, cytokines and chemokines, focusing on only a few pro-inflammatory mediators may not be adequate in determining whether a drug is effective at reducing inflammation. To confirm that pharmacological sEH inhibition does reduce inflammation in LPV, we need to also assess a broader range of cytokines and chemokines upon sEH inhibitor treatment.

**FIGURE 6:**
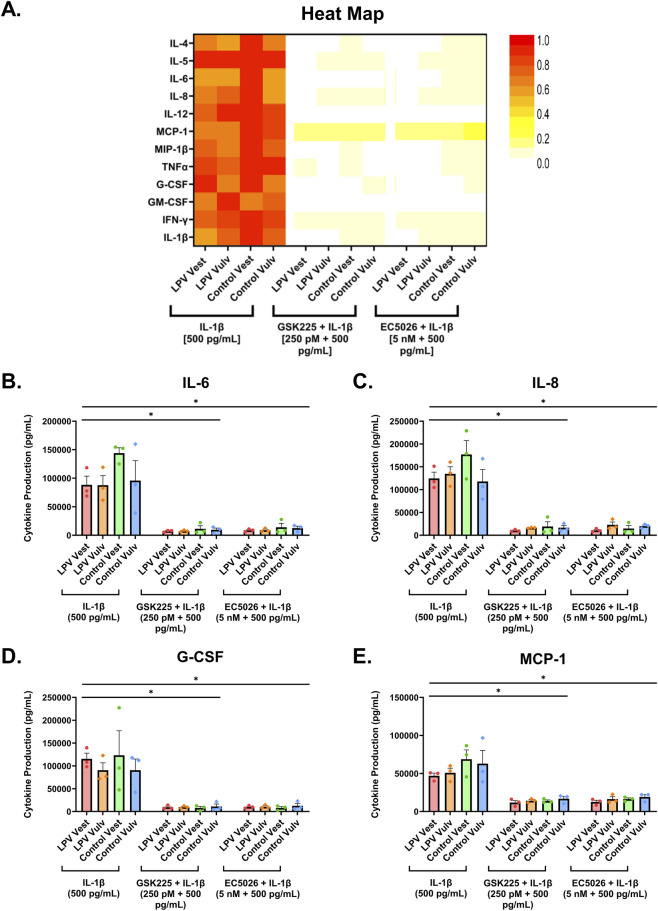
GSK2256294 and EC5026 treatment significantly decreased global pro-inflammatory cytokine levels *in vitro*. **(A)** Heatmap of 12 cytokines detected in vulvar and vestibular fibroblasts upon 48-h treatment with IL-1β [500 pg/mL] or a combination of IL-1β [500 pg/mL] and GSK2256294 [250 pM], or EC5026 [5 nM]. This heat map illustrates changes in cytokine levels between treatment conditions. For a given cytokine, all values were scaled relative to the highest observed quantity of that cytokine within the dataset, which was set to 1 (or 100%). Heatmap colors represent relative changes within each cytokine across treatments. Individual bar graphs of **(B)** IL-6, **(C)** IL-8, **(D)** G-CSF, and **(E)** MCP-1. The data was collected in technical duplicates and are expressed as mean ± SEM, n = 3 cases and 3 controls, two-way ANOVA + Tukey’s *post hoc* test, *P < 0.05.

To assess the global anti-inflammatory effects of sEH inhibition in LPV fibroblasts, cytokine secretion profiles were analyzed following 48-h treatment with IL-1β alone or in combination with either GSK2256294 or EC5026. A Luminex multiplex assay revealed that sEH inhibition markedly reduced a wide spectrum of pro-inflammatory cytokines and chemokines in LPV-derived vestibular fibroblasts, as well as control-derived fibroblasts (Supplementary Table S3; Supplementary Figure S3). Heatmap analysis demonstrated that IL-1β stimulation alone elicited a robust upregulation of a broad panel of pro-inflammatory cytokines, including interleukins (IL)-4, -5, -6, -8, and -12, monocyte chemoattractant protein-1 (MCP-1), tumor necrosis factor alpha (TNF-α), granulocyte colony stimulating factor (G-CSF), interferon-gamma (IFN-γ), and IL-1β itself (Figure 6A). This pattern was consistent across both LPV and control fibroblasts, confirming IL-1β’s potent inflammatory effect. Individual plots of the most distinguished cytokines confirmed these trends. Compared to other cytokines, LPV vestibular and vulvar fibroblast strains produced the most robust levels of IL-6 and IL-8 upon IL-1β stimulation, with the highest concentration being 200,000 and 400,000 pg/mL respectively (Figures 6B,C).

In response to both inhibitors, there was a strong suppression of nearly all the tested cytokines across all fibroblast strains. Both GSK2256294 and EC5026 treatment nearly ablated IL-6 and IL-8 levels (P < 0.05; Figures 6B,C). Similarly, G-CSF and MCP-1, both elevated upon IL-1β stimulation, were also significantly attenuated by either GSK2256294 or EC5026 (P < 0.05; Figures 6D,E). These data demonstrate that both sEH inhibitors exert broad anti-inflammatory effects *in vitro*, reinforcing their potential as targeted therapies for LPV by downregulating multiple cytokines and chemokines implicated in both nociception and inflammation.

### 3.5 Pharmacological sEH inhibition does not affect TRPV4 Ca^2+^ influx *in vitro*

Transient receptor potential vanilloid 4 (TRPV4), a member of the transient receptor potential vanilloid (TRPV) channel family, is a polymodal, non-selective ion channel permeable to calcium (Zhang et al., 2023). TRPV4 is widely expressed across various tissue and cell types, including both sensory and non-sensory cells, and plays a role in various physiological processes such as inflammation (An et al., 2023; Pairet et al., 2018; Walter et al., 2016) and mechanical allodynia (Silverman et al., 2020; Dias et al., 2019). Previous studies have shown that TRPV4 activation contributes to the culmination of pain experienced by LPV patients. Not only is TRPV4 more highly expressed in the painful vestibule of LPV patients compared to controls (Bekauri et al., 2024), but pro-inflammatory mediators that are elevated in the LPV vestibule, such as PGE2, also further perpetuate TRPV4 activation (Cambria et al., 2021; Luo et al., 2018). Additionally, existing literature suggests that TRPV4 can also be activated by certain EETs, namely, 5,6-EET and 8,9-EET (Watanabe et al., 2003; Vriens et al., 2005). Because sEH inhibition alters EET metabolism, we next examined whether pharmacological sEH inhibition affects TRPV4-associated calcium signaling in LPV-derived fibroblasts.

To measure TRPV4 calcium flux in response to pharmacological sEH inhibition, a calcium imaging technique was used where changes in the calcium flux of multiple case and control fibroblast strains were monitored simultaneously in response to varying drug treatments using a FlexStation 3 plate reader. Cells were treated overnight with IL-1β alone, or a combination of IL-1β and GSK2256294, EC5026, or HC-067047 (TRPV4 antagonist). The next day, cells were stimulated with 1 µM of either 5,6-EET, 8,9-EET, 11,12-EET, 14,15-EET, or GSK1016790A (TRPV4 agonist), which was added concomitantly to each well by the FlexStation 3 plate reader during assaying. Again, IL-1β was used as the inflammatory instigator here because cells that were not pretreated with IL-1β did not respond to stimulation with a TRPV4 agonist (Bekauri et al., 2024).

**FIGURE 7:**
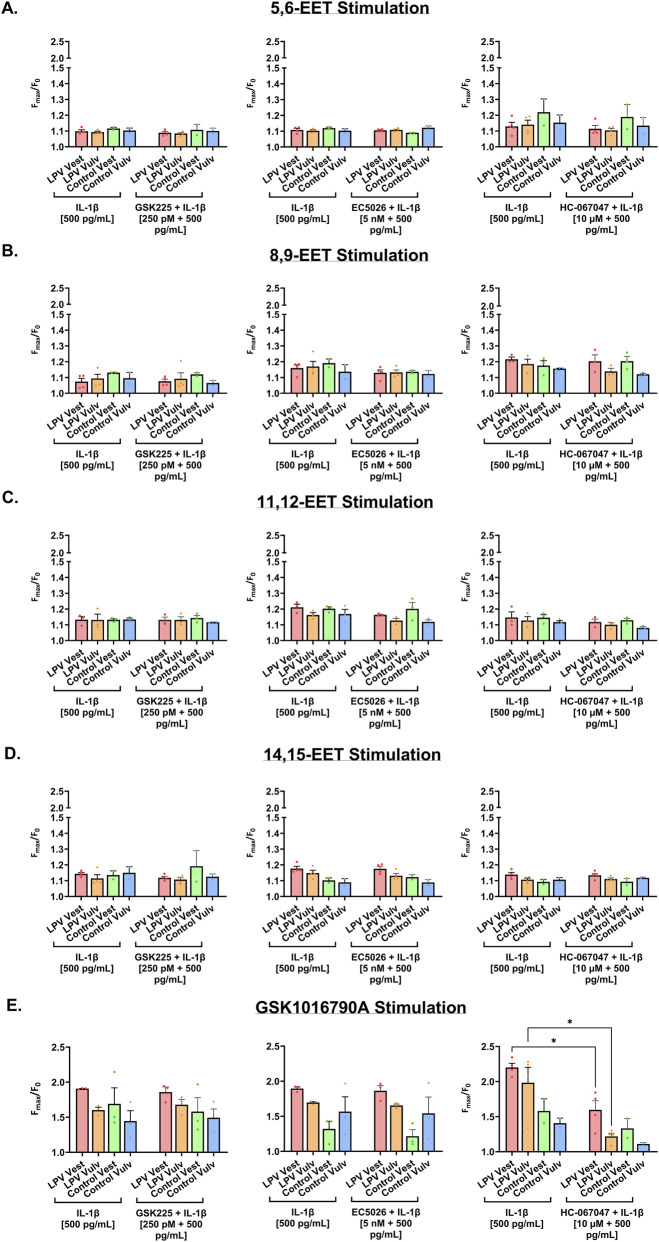
Effect of GSK2256294 and EC5026 on TRPV4 Ca^2+^ responses in vulvar fibroblasts. Calcium influx in vulvar and vestibular fibroblasts treated with IL-1β [500 pg/mL] or a combination of IL-1β [500 pg/mL] and GSK2256294 [250 pM], EC5026 [5 nM], or the TRPV4 antagonist HC-067047 [10 µM] in response to stimulation with **(A)** 1 µM 5,6-EET, **(B)** 1 µM 8,9-EET, **(C)** 1 µM 11,12-EET, **(D)** 1 µM 14,15-EET, and **(E)** 1 µm GSK1016790A. The data was collected in technical quadruplicates and are expressed as mean ± SEM, n = 3 cases and 3 controls, two-way ANOVA + Tukey’s *post hoc* test, *P < 0.05. A split y-axis, indicated by parallel lines, was used to ensure that the scale of the y-axis remained consistent throughout the figure without compromising smaller Fmax/F_0_ values.

Stimulation with each of the EET isoforms (5,6-EET, 8,9-EET, 11,12-EET, and 14,15-EET) resulted in minimal calcium responses across all treatment conditions (Figures 7A–D). In addition, pre-treatment with sEH inhibitors did not significantly amplify EET-induced calcium signaling compared to treatment with IL-1β alone, indicating that the increased EET/DHET ratio resulting from sEH inhibition did not augment calcium influx. Interestingly, overnight treatment with the TRPV4 antagonist HC-067047, followed by stimulation with each individual EET species, did not significantly reduce calcium responses compared to IL-1β-only treatment. This suggests that although EETs are endogenous TRPV4 agonists, they may also be involved in the regulation of other calcium channels. 5,6-EET, but not other EETs, has been recently implicated as an endogenous activator of Transient Receptor Potential Ankyrin 1 (TRPA1) channels (Sisignano et al., 2012), a polymodal calcium-permeable channel involved in thermosensation and pain (Zhang et al., 2022). To further explore the contributions of 5,6-EET agonism in calcium signaling in vulvar fibroblasts, cells were treated overnight with IL-1β alone or a combination of IL-1β, HC-067047, and HC-030031 (TRPA1 antagonist), followed by stimulation with 5,6-EET. Again, co-treatment with both the TRPV4 and TRPA1 antagonist did not attenuate calcium responses compared to IL-1β treatment alone (Supplementary Figure S4).

As a positive control, LPV and control fibroblast strains were also stimulated with the TRPV4-selective agonist GSK1016790A. GSK1016790A stimulation on cells treated overnight with IL-1β alone or a combination of IL-1β and GSK2256294 or EC5026 resulted in robust calcium responses, which was significantly attenuated by treatment with HC-067047 in LPV case vest and vulv fibroblast strains (P < 0.05; Figure 7E). This indicates that LPV fibroblasts have functional TRPV4 channels, and the observed calcium flux is indeed TRPV4-specific. Co-treatment with either GSK2256294 or EC5026 did not enhance calcium responses to GSK1016790A beyond those observed with IL-1β alone. Together, these findings demonstrate that while LPV-derived fibroblasts express functional TRPV4 channels, pharmacological sEH inhibition does not augment TRPV4-associated calcium flux.

## 4 Discussion

Current treatment options for vulvodynia remain inadequate, with no approved pharmacologic agents that reliably target the underlying disease mechanisms (Merlino et al., 2022; Falsetta et al., 2017; Goldstein et al., 2016). In this study, we explored pharmacological sEH inhibition as a targeted approach to resolve inflammation in LPV by regulating the degradation of EETs. Given the evidence of impaired inflammation resolution and diminished EET levels in LPV tissue biopsies (Bekauri et al., 2024), sEH represents a promising therapeutic target to restore the balance between inflammation and resolution and alleviate chronic vulvar pain.

Fibroblasts derived from the painful vestibule of LPV patients exhibit a hyperinflammatory phenotype compared to the external vulva and that of controls (Falsetta et al., 2021; Foster et al., 2015; Falsetta et al., 2016). These cells retain this behavior in culture, producing key mediators such as IL-6, IL-8, and PGE2 which correlate with patient pain thresholds (Foster et al., 2015). It is likely that other cell types, mainly mast cells, afferent nerve fibers, and other immune cells, also contribute to LPV pathogenesis (Tonc et al., 2023; Barry et al., 2019; Liao et al., 2017). However, due to the localized aspect of the disease, the fact that fibroblasts derived from different anatomical sites display distinct gene expression profiles (Chang et al., 2002; Lendahl et al., 2022; Rinn et al., 2006), including those implicated in cell surface receptor expression, lipid metabolism, and cytokine production, makes them a highly representative model of *in vivo* disease conditions.

The selective sEH inhibitor TPPU effectively inhibited sEH activity in vestibular fibroblasts in a dose-dependent manner. Lipidomic analysis of TPPU-treated cells revealed that despite there being no significant increase in EET concentrations, a consistent and significant reduction in their corresponding diol metabolites, DHETs, was observed. This finding may appear confusing given that sEH inhibition should, in principle, increase EETs by preventing their conversion into DHETs. However, several explanations could account for this outcome. First, EETs are highly lipophilic and can be rapidly incorporated into cellular membranes which can sequester them in compartments that are not captured in standard lipidomic analyses (Spector et al., 2004; Spector and Norris, 2007). Additionally, EETs may be rapidly metabolized by alternative pathways, such as β-oxidation (Fang et al., 2002) or conjugation with glutathione (Spearman et al., 1985), both of which can occur independently of sEH. Therefore, the elevated EET/DHET ratio observed after sEH inhibition is likely a more reliable indicator of a change in EET levels than absolute EET concentrations.

Lipidomic analysis revealed that sEH inhibition shifted the lipid mediator profile away from inflammation via reductions in select prostaglandins and leukotrienes, while increasing the abundance of certain specialized pro-resolving mediators, and also reducing the pro-inflammatory cytokines IL-6 and IL-8. These shifts are consistent with prior studies demonstrating that EETs can modulate enzymatic activity and downstream mediator production within both the COX and LOX pathways. For example, 11,12-EET and 14,15-EET have been shown to suppress PGE2 production in multiple murine cell types (Fang et al., 1998; Kozak et al., 2003). Similarly, EETs can also interfere with the 5-lipoxygenase (5-LOX) pathway, leading to reduced LTB4 synthesis (Jiang et al., 2015). The present lipidomic findings and ELISA data, namely, reductions in 9-HODE, LTB4, and PGE2, along with increases in SPMs, are consistent with this broader lipid reprogramming effect and support the hypothesis that sEH inhibition restores EET-mediated resolution across multiple branches of the AA cascade.

To advance the translational relevance of sEH inhibition, two clinically relevant compounds, GSK2256294 and EC5026, were evaluated. Both inhibitors suppressed sEH activity at low nanomolar concentrations and reproduced the anti-inflammatory effects observed with TPPU. A broader analysis of cytokine secretion using multiplex assays confirmed that sEH inhibition suppressed a wide array of pro-inflammatory cytokines and chemokines, including G-CSF, TNF-α, and MCP-1. These mediators play well-established roles in immune cell recruitment and pain sensitization. G-CSF stimulates the mobilization and activation of neutrophils (Martin et al., 2021) and has been shown to contribute to hyperalgesia by acting directly on the dorsal root ganglion (DRG) in a rat model of pathological pain (Yeh et al., 2021). TNF-α is a key initiator of inflammatory cascades, promoting both leukocyte recruitment and the upregulation of nociceptive pathways through sensitization of TRP channels (Sedger and Mcdermott, 2014; García-Domínguez, 2025). MCP-1, also known as CCL2, has been directly implicated in the development and maintenance of neuropathic pain by inducing microglia activation (Alvarez et al., 2014; Thacker et al., 2009; Zhang and De Koninck, 2006). The reduction of these factors by sEH inhibitors is relevant to the mast cell hypothesis of vulvodynia, which proposes that increased mast cell accumulation and degranulation in the vulvar vestibule promotes nerve sprouting and maintains hypersensitivity, thereby contributing to pain and inflammation in LPV (Tonc et al., 2023). By attenuating cytokines and chemokines involved in immune cell recruitment and activation, sEH inhibition may indirectly reduce mast cell-driven hyperalgesia in LPV.

Because certain EET isomers, namely, 5,6-EET and 8,9-EET, can directly bind to and activate TRPV4 (Vriens et al., 2005; Watanabe et al., 2003), and TRPV4 has been implicated in LPV-associated vulvar pain (Bekauri et al., 2024), we assessed whether sEH inhibition could potentiate TRPV4-mediated calcium flux. Calcium imaging assays revealed that neither sEH inhibition nor EET stimulation enhanced TRPV4-mediated calcium influx in fibroblasts. Because fibroblasts are not sensory cells, these findings should not be interpreted as direct evidence regarding nociceptive signaling, but simply as an assessment that increasing EET/DHET ratio does not exacerbate TRPV4 activation in fibroblasts under the conditions tested. It is possible that while measurable increases in EET/DHET ratio were observed, the EET concentrations reaching the channel were insufficient for channel activation. Because 11,12-EET and 14,15-EET were the predominant EET species produced by these fibroblasts, and the effect of these EETs on TRPV4 is more debated (Arnold et al., 2025; Vriens et al., 2005), there is a low likelihood that sEH inhibition will increase TRPV4 activation *in vitro*. While *in vivo* studies are needed to fully assess nociceptive effects, the data suggest that sEH inhibition can reduce inflammation without exacerbating TRPV4-mediated calcium flux in fibroblasts.

While these results strongly support the role of sEH in LPV pathogenesis and its inhibition as a promising therapeutic strategy, several limitations should be noted. First, this study relied on primary fibroblast cultures, which do not fully recapitulate the multicellular environment of the vulvar vestibule. Studies utilizing a validated animal model of LPV (Falsetta et al., 2021; Bekauri et al., 2024; Farmer et al., 2011) are necessary to corroborate the efficacy and safety of sEH inhibitors in reducing pain and inflammation *in vivo*. Furthermore, while this study focused on inflammatory signaling, LPV is now understood to be a complex, multifactorial disorder that likely involves multiple pathways aside from inflammation that are not yet fully defined. Emerging evidence points to contributions from genetic predispositions, hormonal influences, and neuroimmune interactions (Torres-Cueco and Nohales-Alfonso, 2021). Thus, stimulating these fibroblasts with IL-1β may only recapitulate a fraction of the disease mechanism. Third, the present cohort consisted entirely of Caucasian, non-Hispanic participants, which limits generalizability. Future studies incorporating more diverse populations are necessary to determine whether these findings extend across broader patient groups. Lastly, there is a discrepancy between previously published findings (Falsetta et al., 2017; Falsetta et al., 2015; Falsetta et al., 2016; Foster et al., 2015; Falsetta et al., 2023) and findings here where some LPV fibroblast strains exhibit weaker inflammatory responses to IL-1β than those from healthy controls. This dampened response may potentially reflect a state of chronic activation, receptor downregulation, or feedback inhibition within inflammatory pathways. These mechanisms are consistent with known features of chronically inflamed fibroblasts, including prolonged NF-κB activation which can lead to IL-1 receptor internalization or reduced surface expression following sustained stimulation (Prescott et al., 2021).

Taken together, these findings identify sEH as a mechanistically relevant therapeutic target for LPV. By restoring the balance of pro-resolving lipid mediators and suppressing global pro-inflammatory cytokines without augmenting TRPV4 signaling in fibroblasts, sEH inhibition offers a promising therapeutic strategy that addresses both the defect in resolution and chronic inflammation in LPV. These results contribute to the expanding body of evidence highlighting pharmacological sEH inhibition as a novel approach to treating chronic inflammation and pain.

## Data Availability

The datasets presented in this study can be found in online repositories. The names of the repository/repositories and accession number(s) can be found in the article/Supplementary Material.
