# Supplementary material for: Soluble epoxide hydrolase inhibition restores pro-resolving lipid mediators and reduces inflammation in localized provoked vulvodynia
Source: Front Pharmacol. 2026 Mar 26;17:1741914. doi: 10.3389/fphar.2026.1741914 (PMC13062195; doi:10.3389/fphar.2026.1741914)
Supplement: Supplementary file 1 [file DataSheet1.pdf]

Figure S1. Individual bar graphs of full lipidomic dataset.
The data was collected in triplicates and are expressed as mean ± SEM; n=6; *P < 0.05.

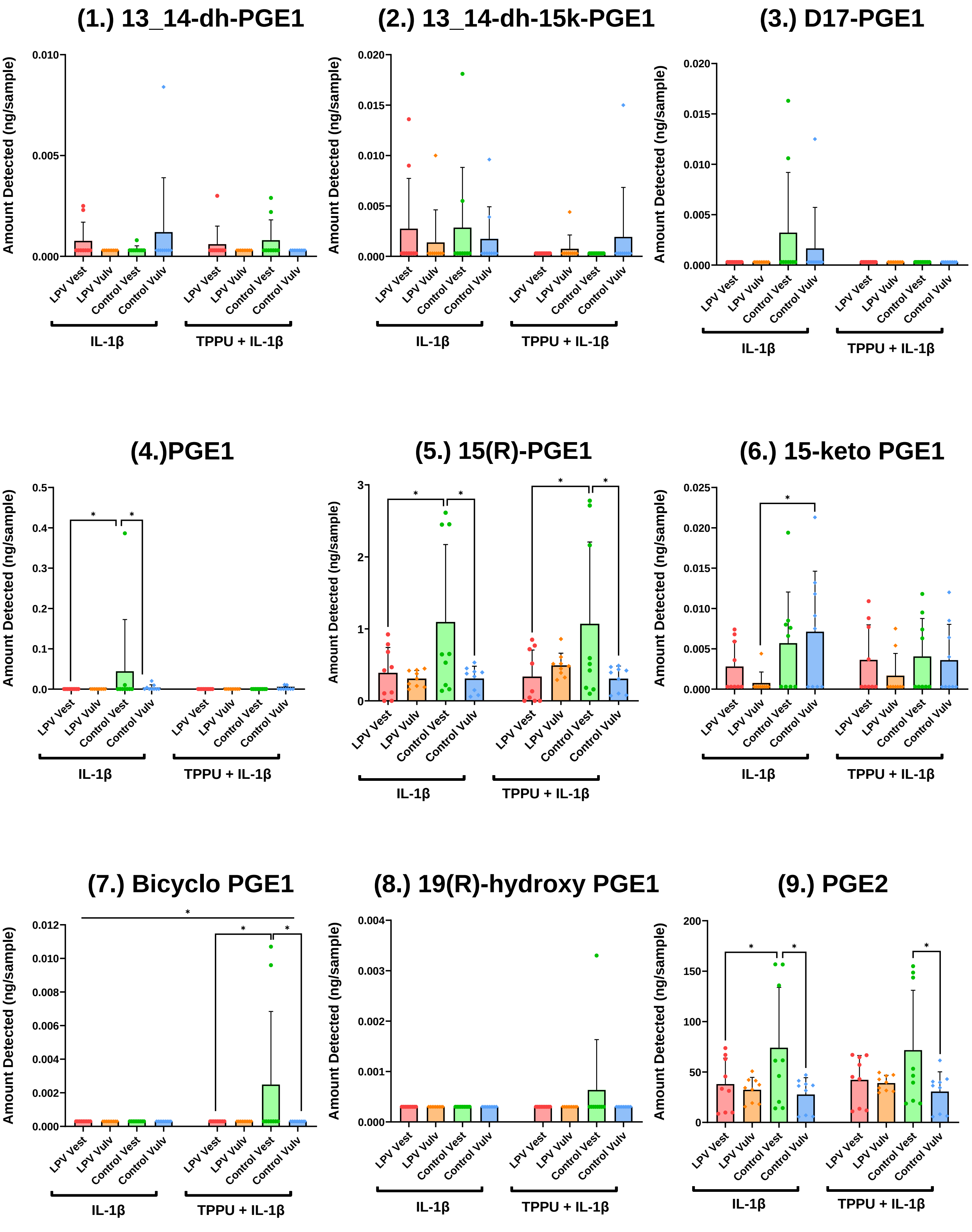

(11.) 15-keto PGE2

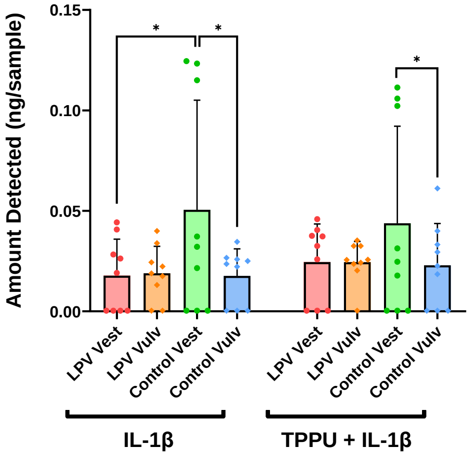

(12.) 13_14-dh-15k-PGE2

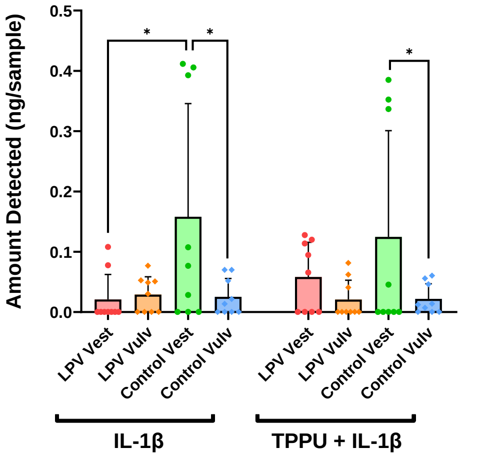

(13.) Bicyclo PGE2

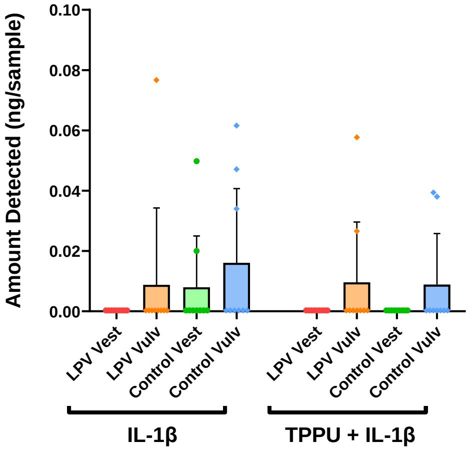

(14.) PGA2

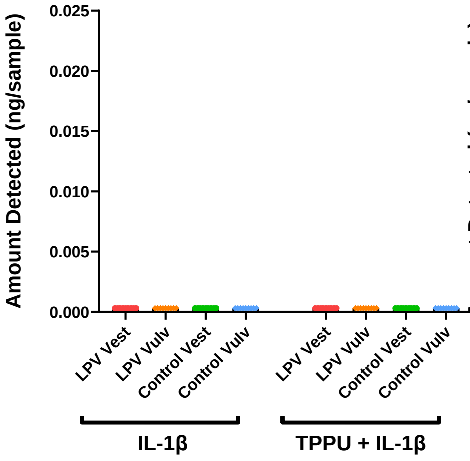

(15.) 19(R)-OH PGE2 & 20-OH PGE2

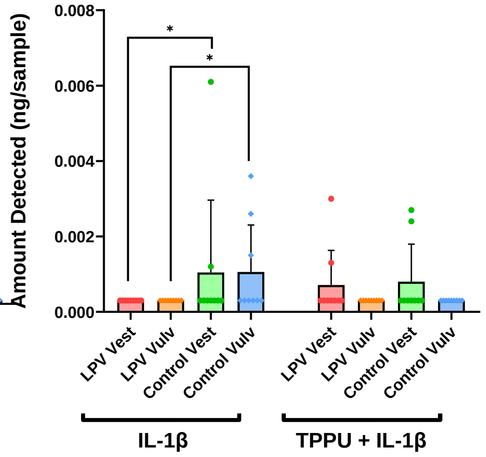

(16.) tetranor PGEM

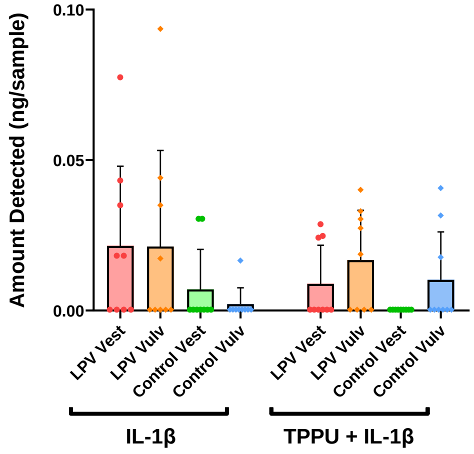

(17.) PGE3

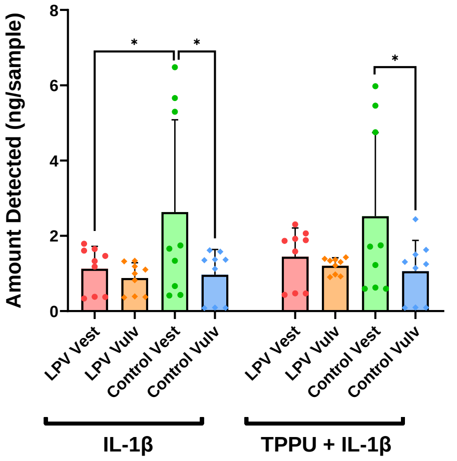

(18.) PGD2

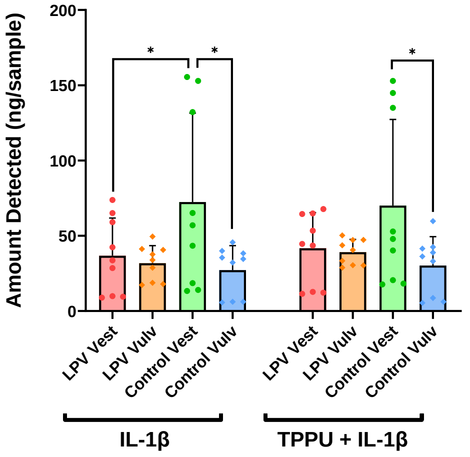

(20.) D12-PGJ2

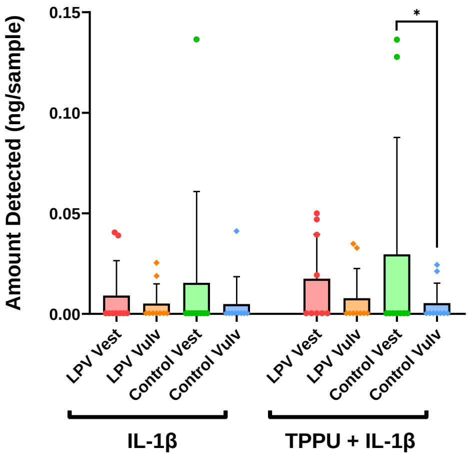

(22.) 13_14-dh-15k-PGD2

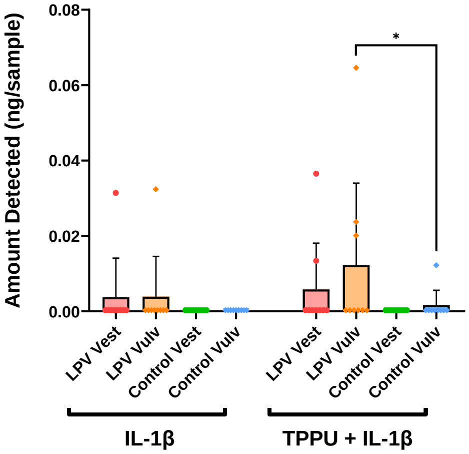

(23.) PGD3

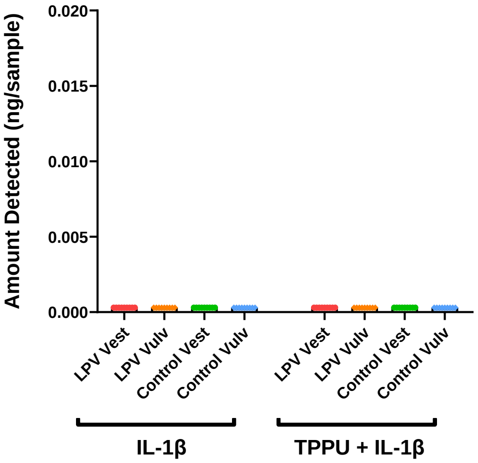

(24.) PGF1a

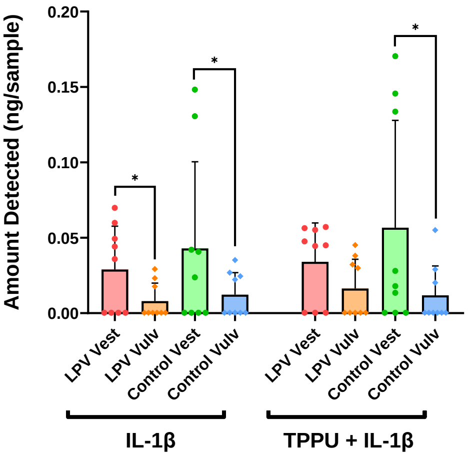

(26.) PGF2a

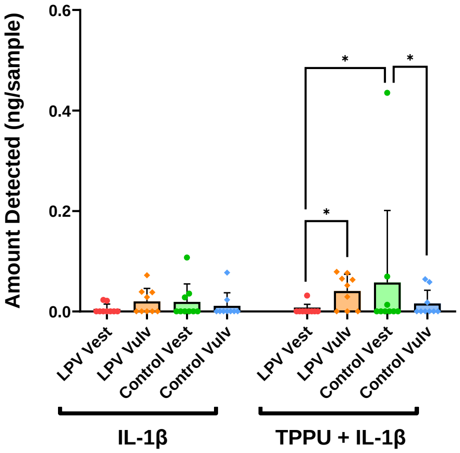

(27.) 15-keto PGF2a

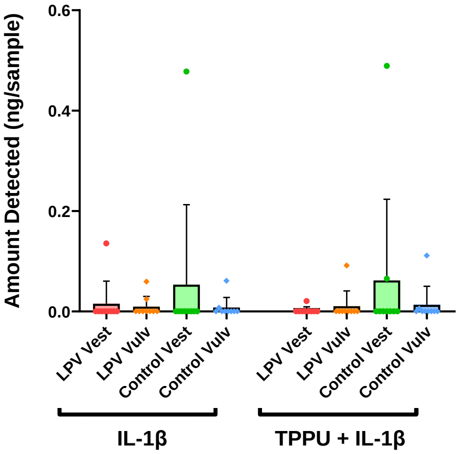

(28.) 13_14-dh-15k-PGF2a

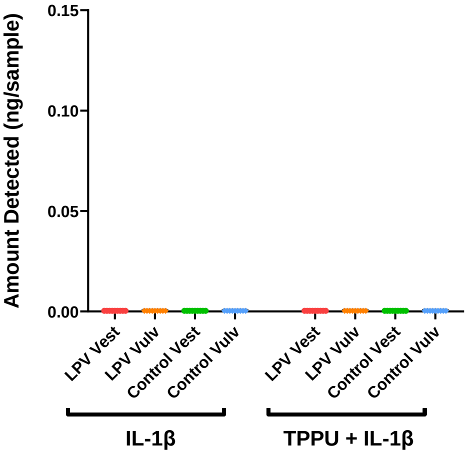

(29.) 19(R)-OH PGF2a & 20-OH PGF2a

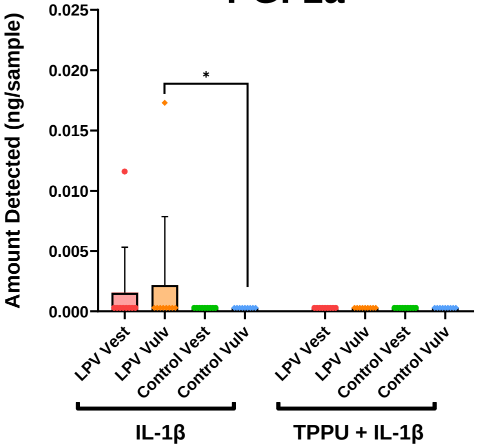

(30.) PGF3a

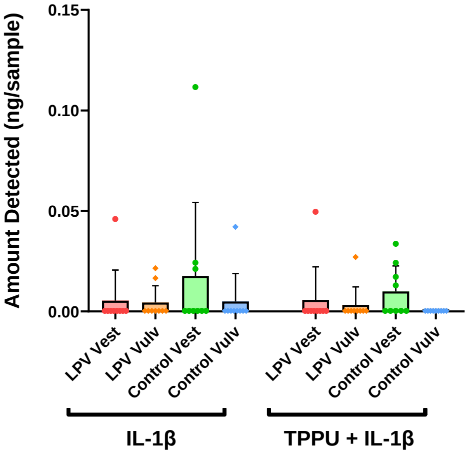

(31.) 8-isoPGF2a & 11bPGF2a

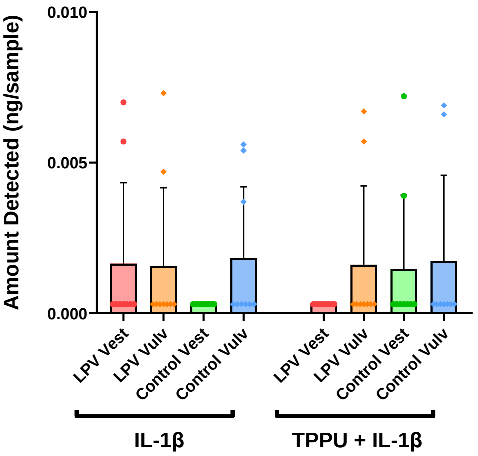

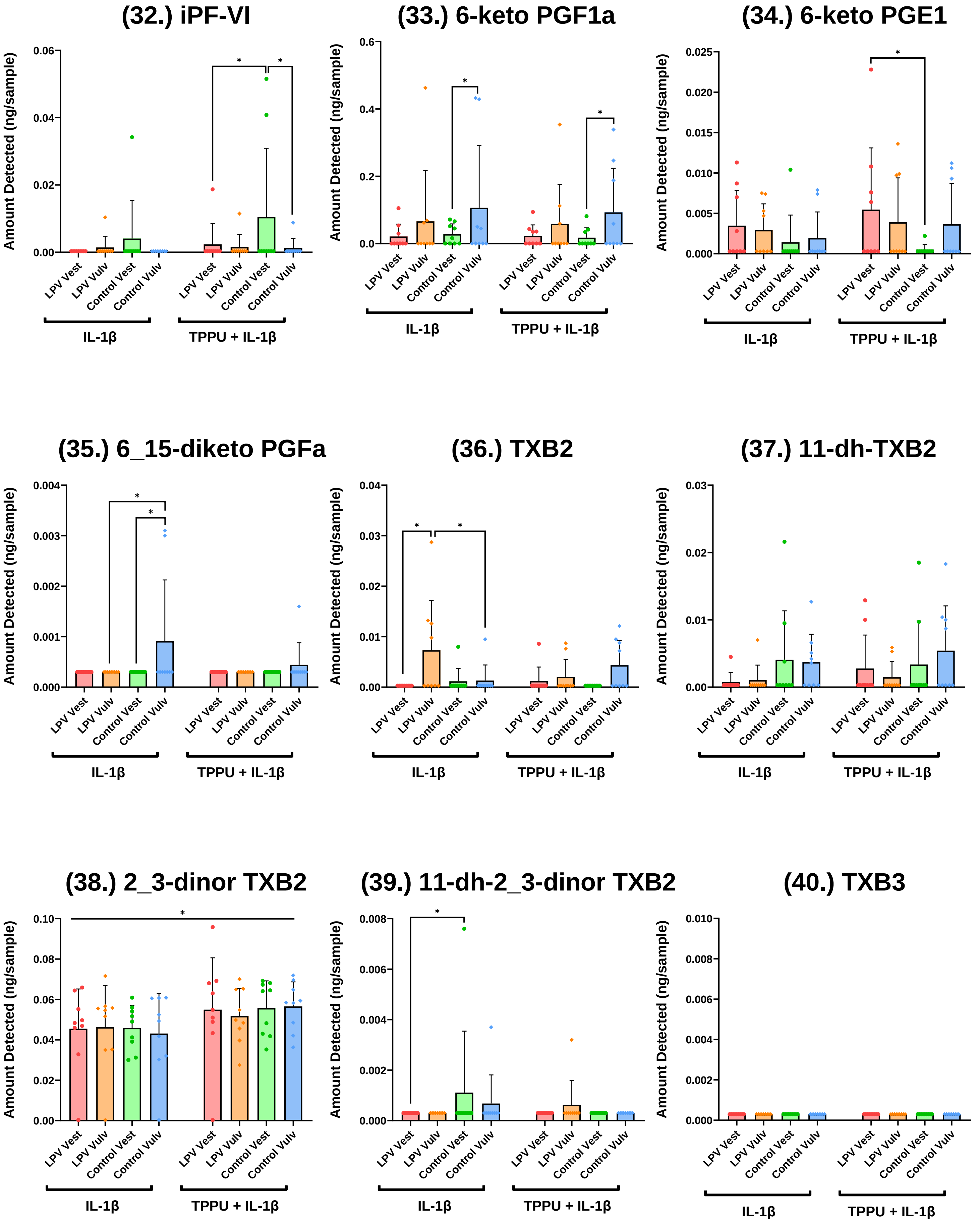
(32.) iPF-VI
(33.) 6-keto PGF1a
(34.) 6-keto PGE1
(35.) 6_15-diketo PGFa
(36.) TXB2
(37.) 11-dh-TXB2
(38.) 2_3-dinor TXB2
(39.) 11-dh-2_3-dinor TXB2
(40.) TXB3
Amount Detected (ng/sample)
LPV Vest
LPV Vulv
Control Vest
Control Vulv
IL-1β
TPPU + IL-1β
*

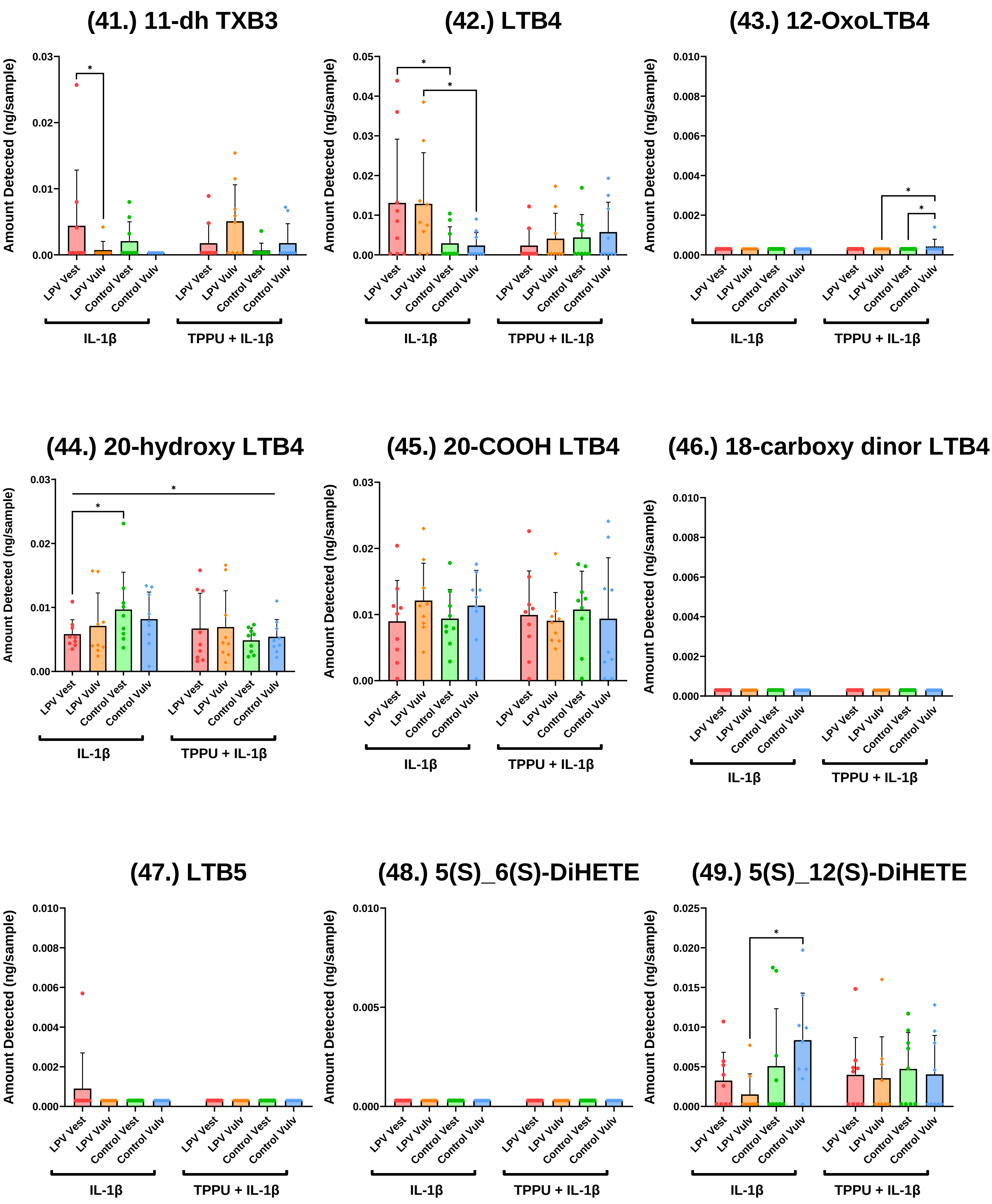
(41.) 11-dh TXB3
(42.) LTB4
(43.) 12-OxoLTB4
(44.) 20-hydroxy LTB4
(45.) 20-COOH LTB4
(46.) 18-carboxy dinor LTB4
(47.) LTB5
(48.) 5(S)_6(S)-DiHETE
(49.) 5(S)_12(S)-DiHETE
Amount Detected (ng/sample)
LPV Vest
LPV Vulv
Control Vest
Control Vulv
IL-1β
TPPU + IL-1β
*

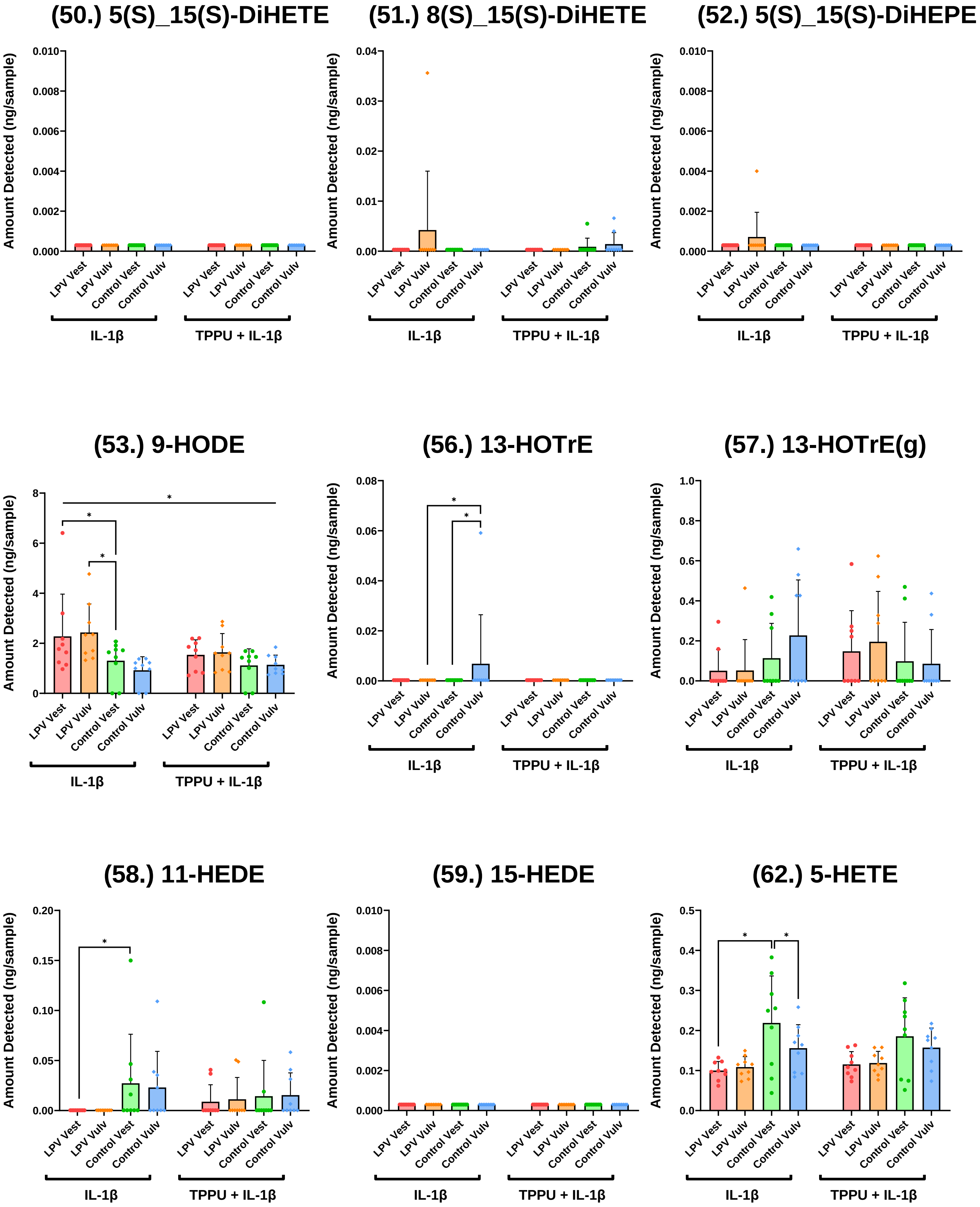

(50.) 5(S)_15(S)-DiHETE
Amount Detected (ng/sample)
0.000
0.002
0.004
0.006
0.008
0.010
LPV Vest
LPV Vulv
Control Vest
Control Vulv
IL-1β
TPPU + IL-1β
(51.) 8(S)_15(S)-DiHETE
Amount Detected (ng/sample)
0.00
0.01
0.02
0.03
0.04
LPV Vest
LPV Vulv
Control Vest
Control Vulv
IL-1β
TPPU + IL-1β
(52.) 5(S)_15(S)-DiHEPE
Amount Detected (ng/sample)
0.000
0.002
0.004
0.006
0.008
0.010
LPV Vest
LPV Vulv
Control Vest
Control Vulv
IL-1β
TPPU + IL-1β
(53.) 9-HODE
Amount Detected (ng/sample)
0
2
4
6
8
*
LPV Vest
LPV Vulv
Control Vest
Control Vulv
IL-1β
TPPU + IL-1β
(56.) 13-HOTrE
Amount Detected (ng/sample)
0.00
0.02
0.04
0.06
0.08
*
LPV Vest
LPV Vulv
Control Vest
Control Vulv
IL-1β
TPPU + IL-1β
(57.) 13-HOTrE(g)
Amount Detected (ng/sample)
0.0
0.2
0.4
0.6
0.8
1.0
LPV Vest
LPV Vulv
Control Vest
Control Vulv
IL-1β
TPPU + IL-1β
(58.) 11-HEDE
Amount Detected (ng/sample)
0.00
0.05
0.10
0.15
0.20
*
LPV Vest
LPV Vulv
Control Vest
Control Vulv
IL-1β
TPPU + IL-1β
(59.) 15-HEDE
Amount Detected (ng/sample)
0.000
0.002
0.004
0.006
0.008
0.010
LPV Vest
LPV Vulv
Control Vest
Control Vulv
IL-1β
TPPU + IL-1β
(62.) 5-HETE
Amount Detected (ng/sample)
0.0
0.1
0.2
0.3
0.4
0.5
*
LPV Vest
LPV Vulv
Control Vest
Control Vulv
IL-1β
TPPU + IL-1β

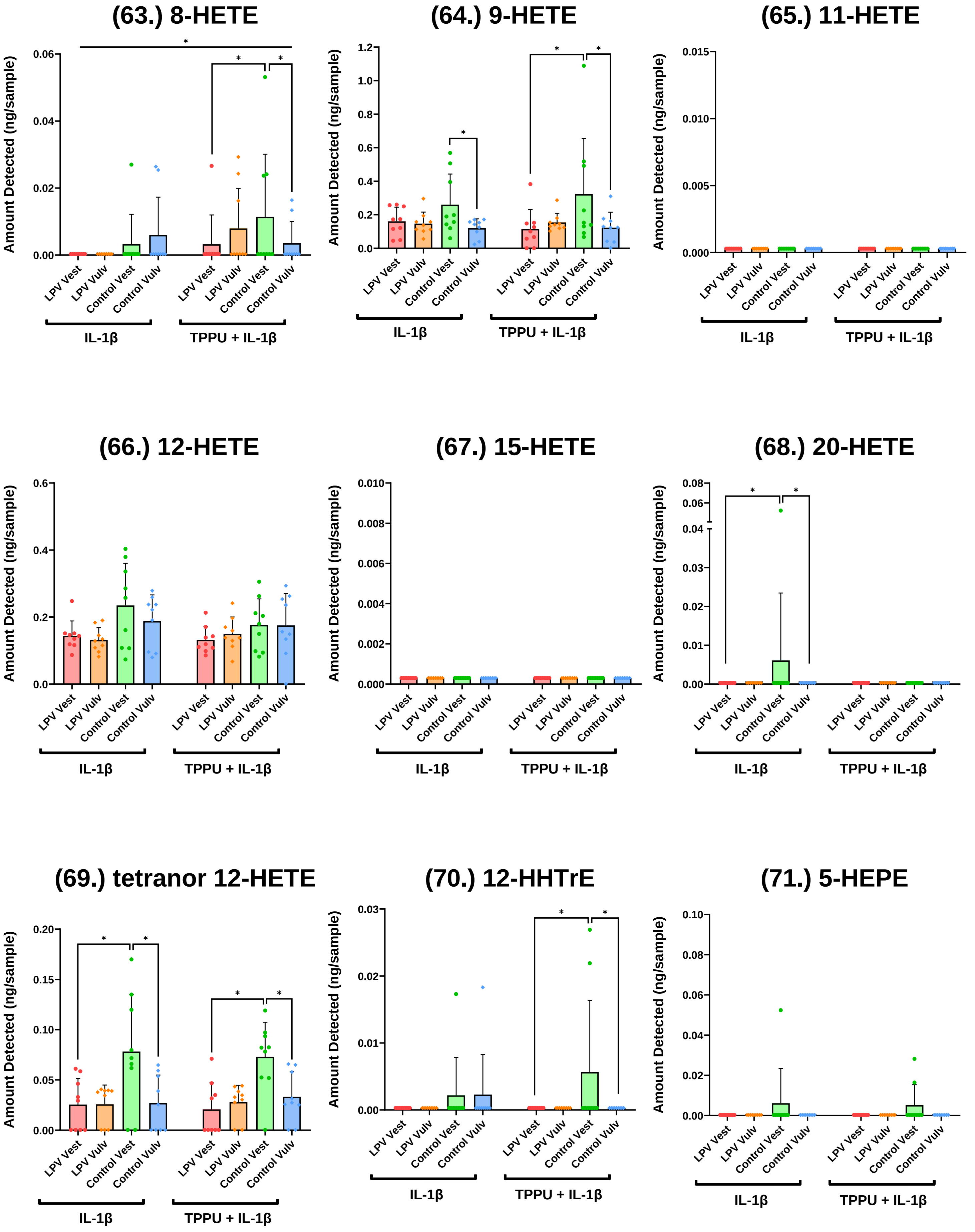
(63.) 8-HETE
(64.) 9-HETE
(65.) 11-HETE
(66.) 12-HETE
(67.) 15-HETE
(68.) 20-HETE
(69.) tetranor 12-HETE
(70.) 12-HHTrE
(71.) 5-HEPE
Amount Detected (ng/sample)
LPV Vest
LPV Vulv
Control Vest
Control Vulv
IL-1β
TPPU + IL-1β

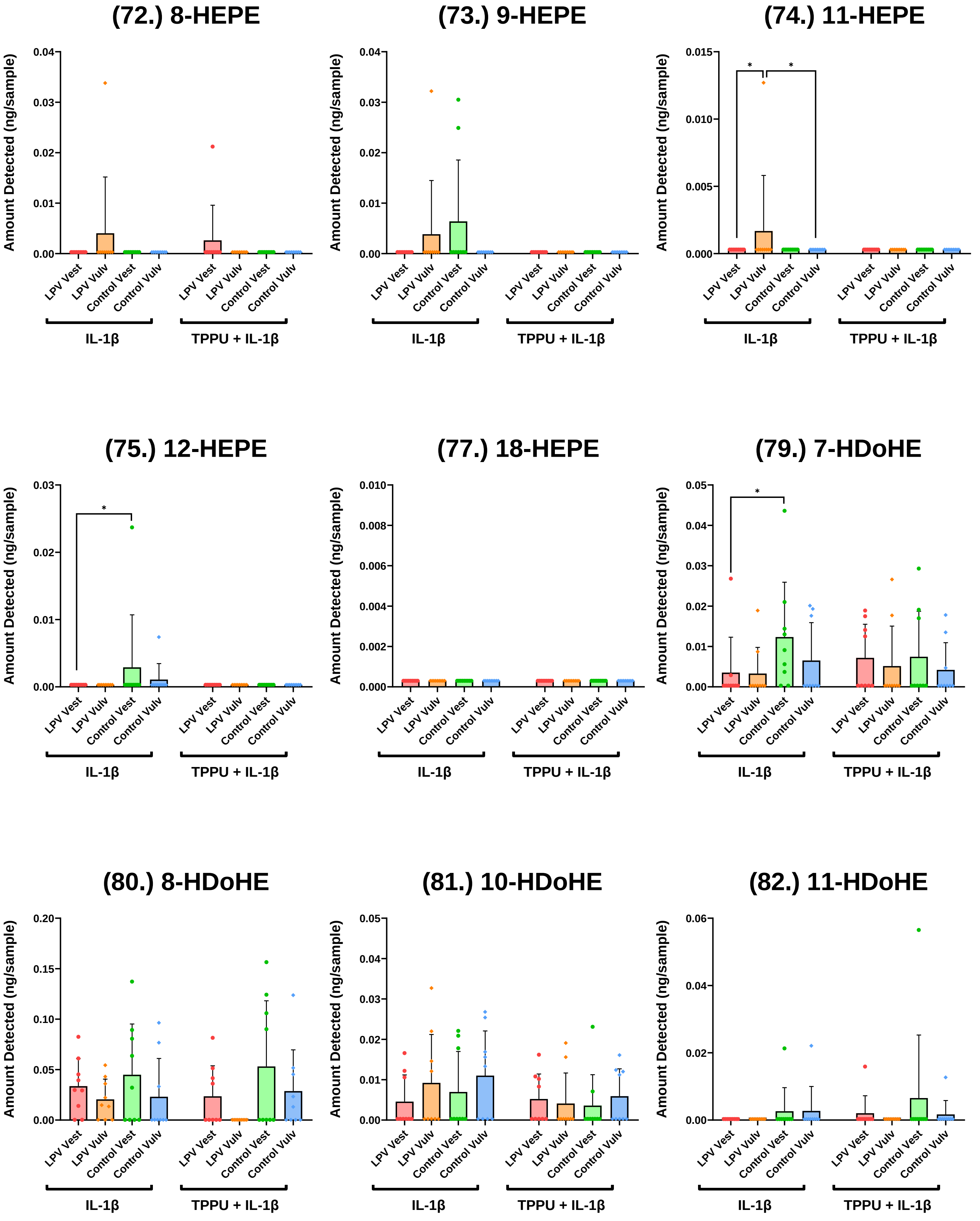

(72.) 8-HEPE
(73.) 9-HEPE
(74.) 11-HEPE
(75.) 12-HEPE
(77.) 18-HEPE
(79.) 7-HDoHE
(80.) 8-HDoHE
(81.) 10-HDoHE
(82.) 11-HDoHE
Amount Detected (ng/sample)
0.00
0.01
0.02
0.03
0.04
0.000
0.005
0.010
0.015
0.002
0.004
0.006
0.008
0.05
0.10
0.15
0.20
0.06
*
LPV Vest
LPV Vulv
Control Vest
Control Vulv
IL-1β
TPPU + IL-1β

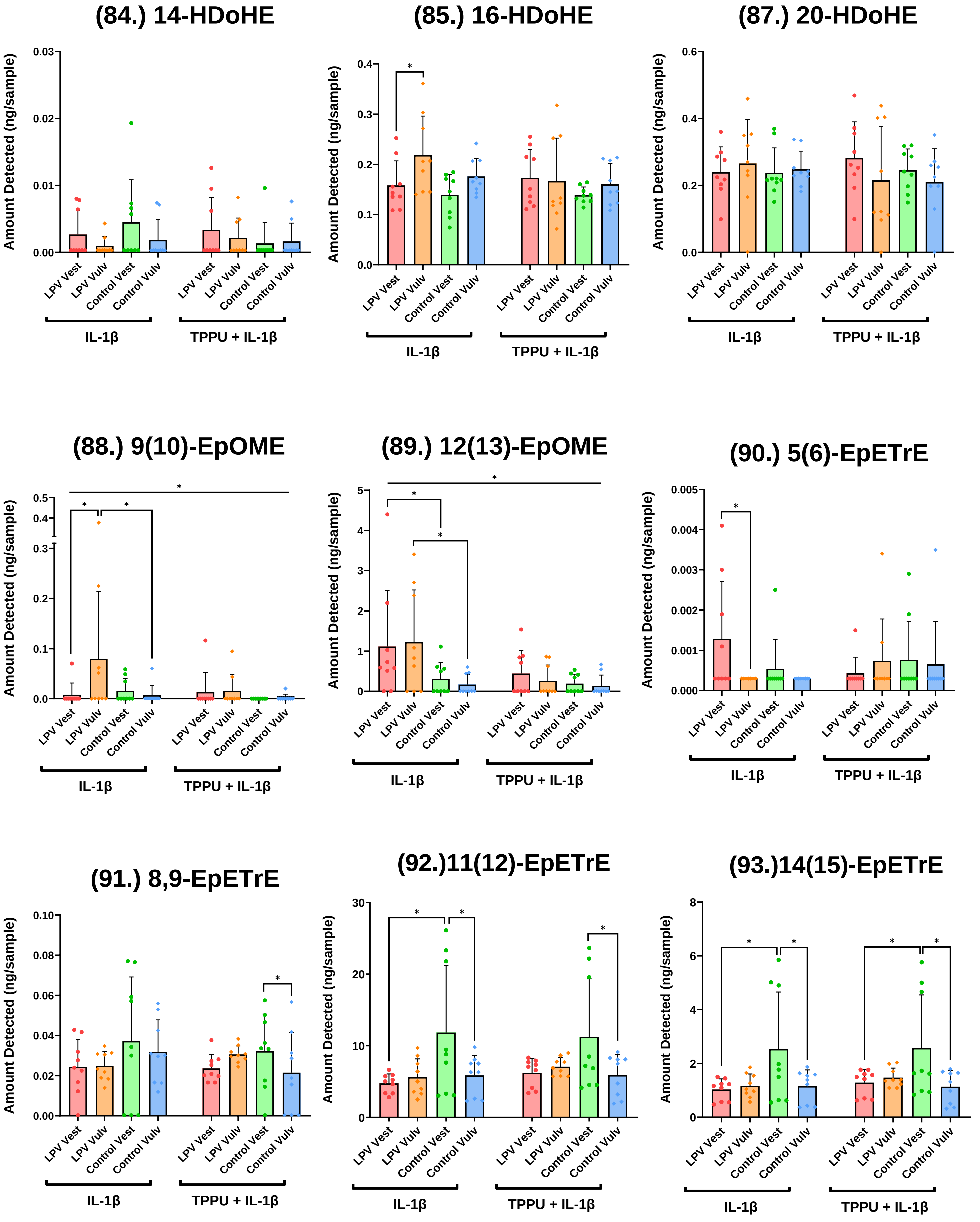
(84.) 14-HDoHE
(85.) 16-HDoHE
(87.) 20-HDoHE
(88.) 9(10)-EpOME
(89.) 12(13)-EpOME
(90.) 5(6)-EpETrE
(91.) 8,9-EpETrE
(92.)11(12)-EpETrE
(93.)14(15)-EpETrE
Amount Detected (ng/sample)
LPV Vest
LPV Vulv
Control Vest
Control Vulv
IL-1β
TPPU + IL-1β
*

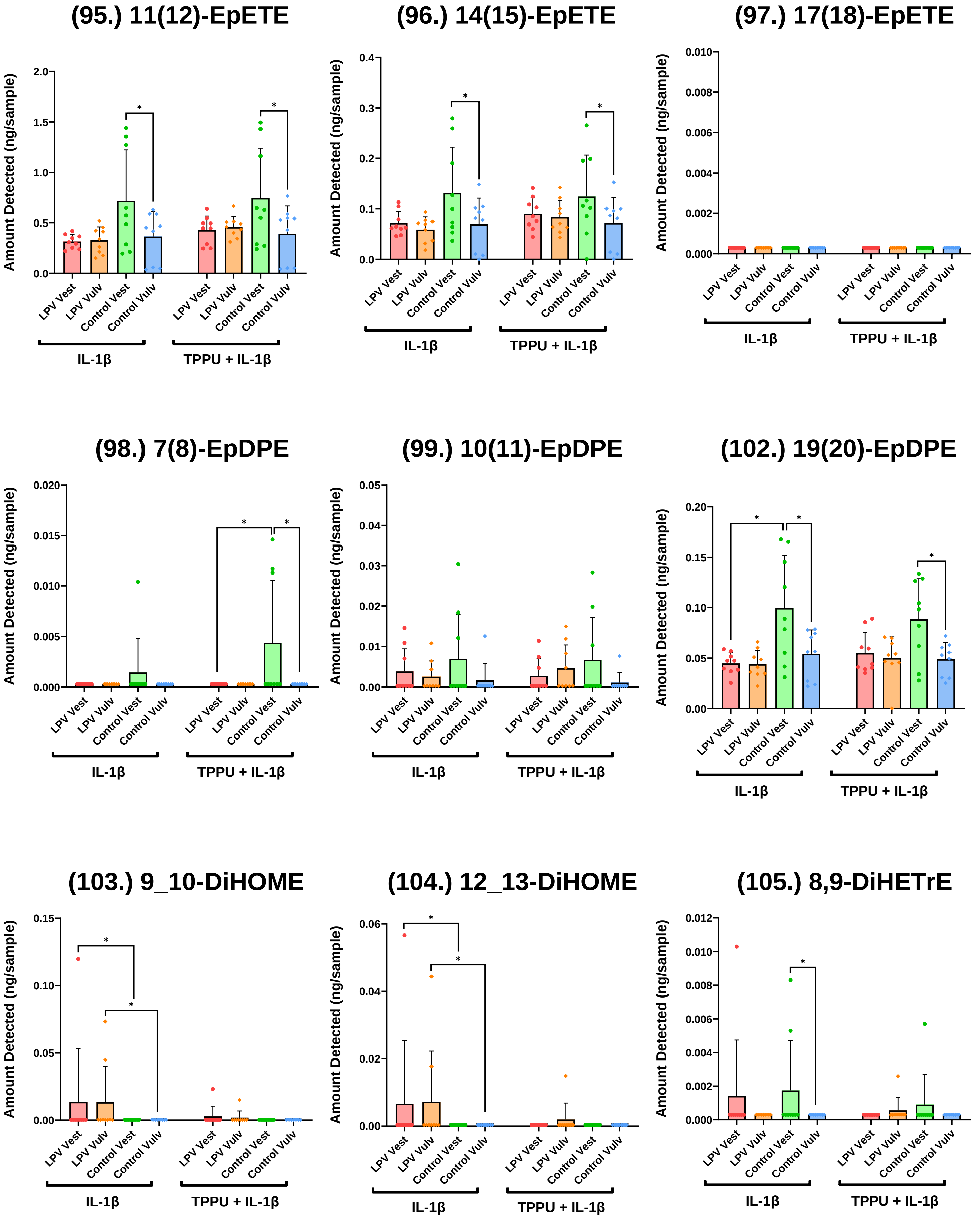

(95.) 11(12)-EpETE
(96.) 14(15)-EpETE
(97.) 17(18)-EpETE
(98.) 7(8)-EpDPE
(99.) 10(11)-EpDPE
(102.) 19(20)-EpDPE
(103.) 9_10-DiHOME
(104.) 12_13-DiHOME
(105.) 8,9-DiHETrE
Amount Detected (ng/sample)
LPV Vest
LPV Vulv
Control Vest
Control Vulv
IL-1β
TPPU + IL-1β
*

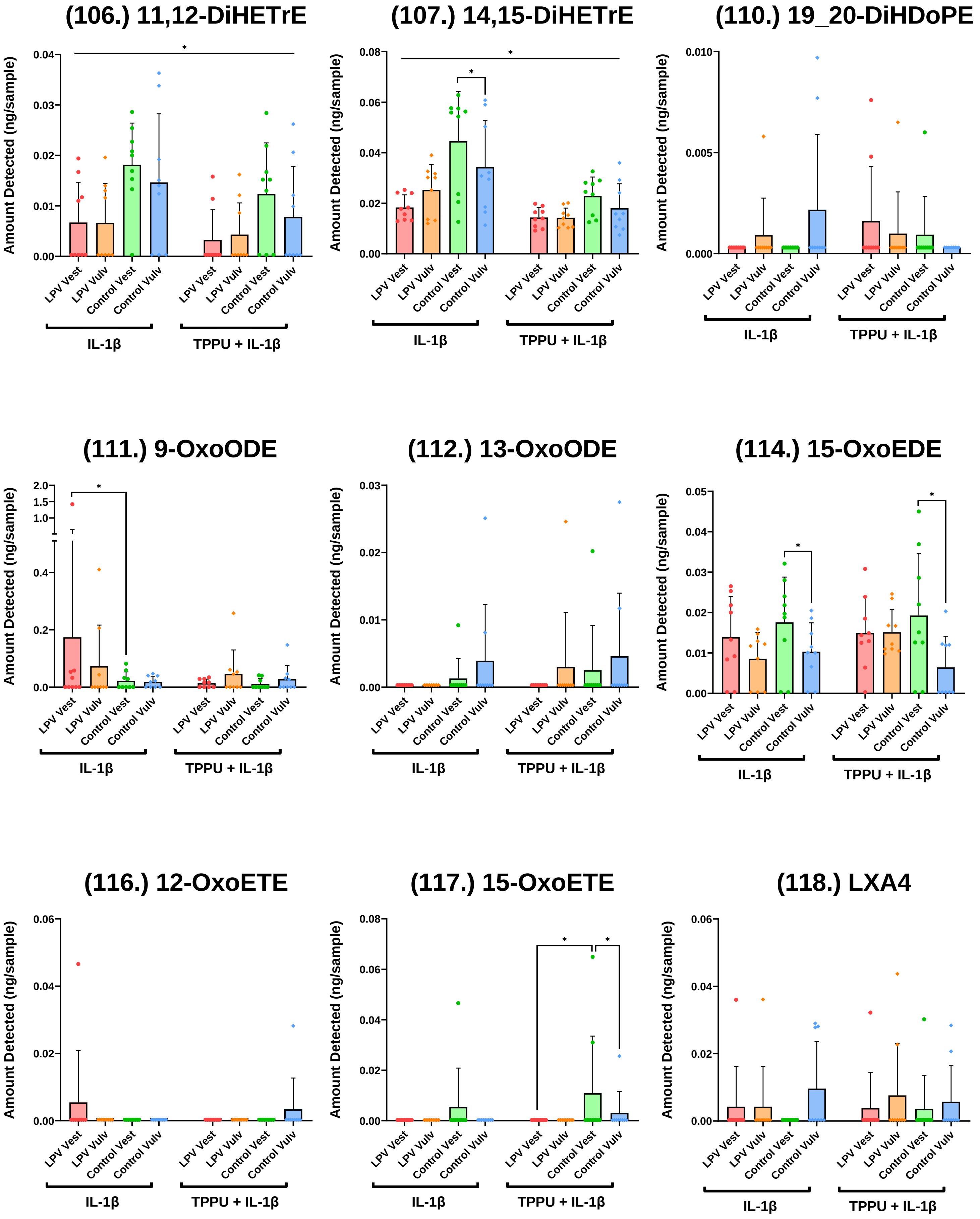

(106.) 11,12-DiHETrE
(107.) 14,15-DiHETrE
(110.) 19_20-DiHDoPE
(111.) 9-OxoODE
(112.) 13-OxoODE
(114.) 15-OxoEDE
(116.) 12-OxoETE
(117.) 15-OxoETE
(118.) LXA4
Amount Detected (ng/sample)
LPV Vest
LPV Vulv
Control Vest
Control Vulv
IL-1β
TPPU + IL-1β
*
0.00
0.01
0.02
0.03
0.04
0.05
0.06
0.08
0.000
0.005
0.010
0.0
0.2
0.4
1.0
1.5
2.0

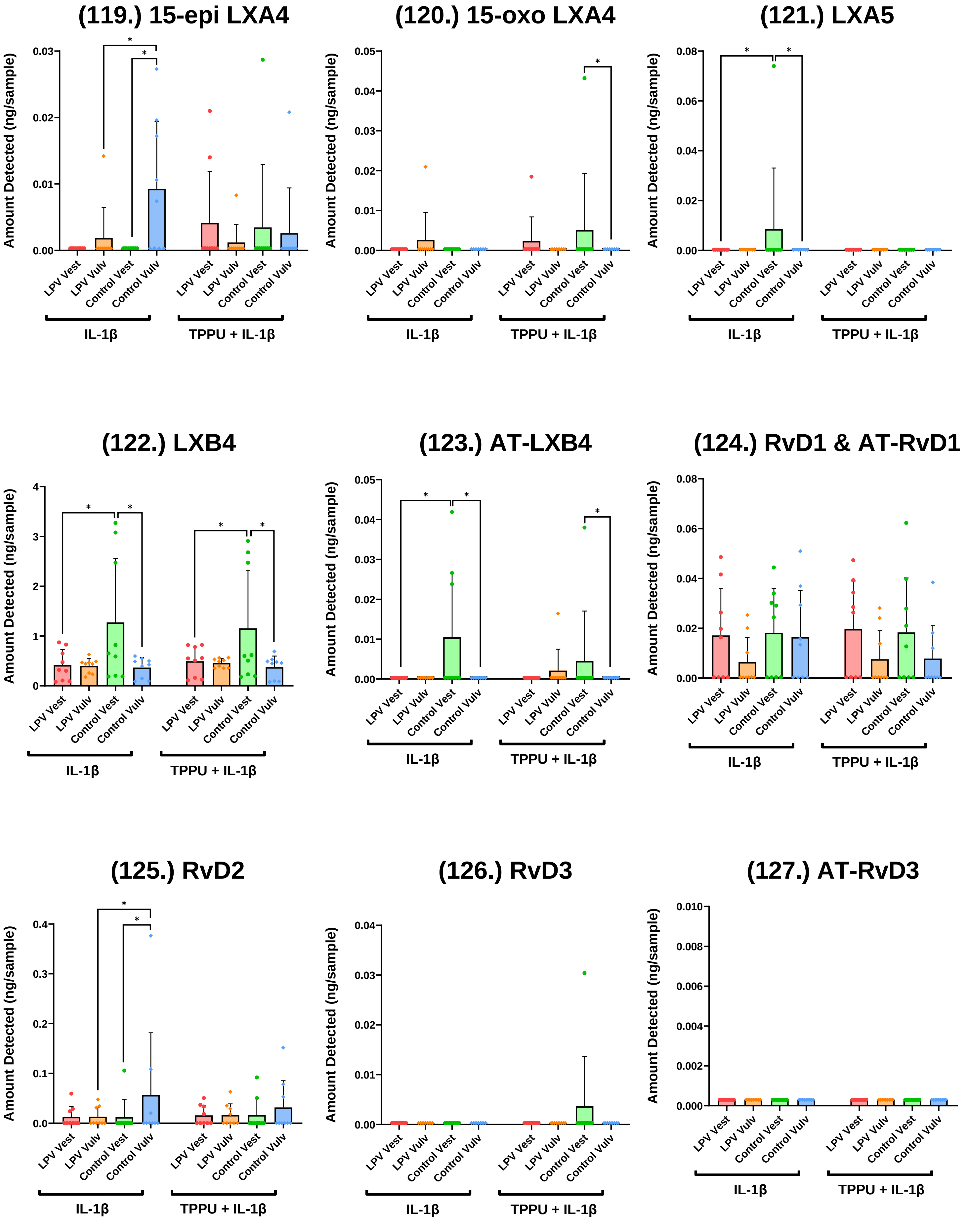

(119.) 15-epi LXA4
Amount Detected (ng/sample)
0.03
0.02
0.01
0.00
*
*
LPV Vest
LPV Vulv
Control Vest
Control Vulv
IL-1β
TPPU + IL-1β
(120.) 15-oxo LXA4
Amount Detected (ng/sample)
0.05
0.04
0.03
0.02
0.01
0.00
*
LPV Vest
LPV Vulv
Control Vest
Control Vulv
IL-1β
TPPU + IL-1β
(121.) LXA5
Amount Detected (ng/sample)
0.08
0.06
0.04
0.02
0.00
*
*
LPV Vest
LPV Vulv
Control Vest
Control Vulv
IL-1β
TPPU + IL-1β
(122.) LXB4
Amount Detected (ng/sample)
4
3
2
1
0
*
*
*
*
LPV Vest
LPV Vulv
Control Vest
Control Vulv
IL-1β
TPPU + IL-1β
(123.) AT-LXB4
Amount Detected (ng/sample)
0.05
0.04
0.03
0.02
0.01
0.00
*
*
*
LPV Vest
LPV Vulv
Control Vest
Control Vulv
IL-1β
TPPU + IL-1β
(124.) RvD1 & AT-RvD1
Amount Detected (ng/sample)
0.08
0.06
0.04
0.02
0.00
LPV Vest
LPV Vulv
Control Vest
Control Vulv
IL-1β
TPPU + IL-1β
(125.) RvD2
Amount Detected (ng/sample)
0.4
0.3
0.2
0.1
0.0
*
*
LPV Vest
LPV Vulv
Control Vest
Control Vulv
IL-1β
TPPU + IL-1β
(126.) RvD3
Amount Detected (ng/sample)
0.04
0.03
0.02
0.01
0.00
LPV Vest
LPV Vulv
Control Vest
Control Vulv
IL-1β
TPPU + IL-1β
(127.) AT-RvD3
Amount Detected (ng/sample)
0.010
0.008
0.006
0.004
0.002
0.000
LPV Vest
LPV Vulv
Control Vest
Control Vulv
IL-1β
TPPU + IL-1β

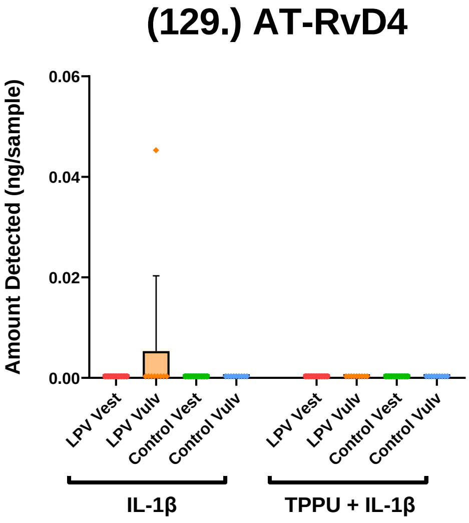
(129.) AT-RvD4
Amount Detected (ng/sample)
0.06
0.04
0.02
0.00
LPV Vest
LPV Vulv
Control Vest
Control Vulv
IL-1β
TPPU + IL-1β

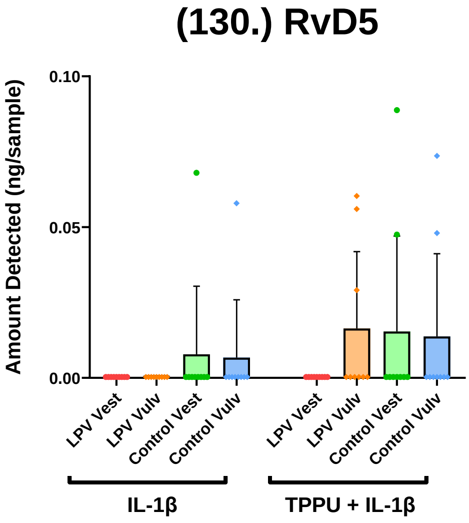
(130.) RvD5
Amount Detected (ng/sample)
0.10
0.05
0.00
LPV Vest
LPV Vulv
Control Vest
Control Vulv
IL-1β
TPPU + IL-1β

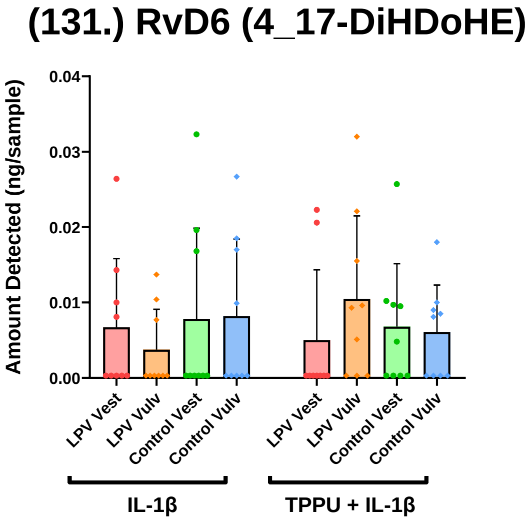
(131.) RvD6 (4_17-DiHDoHE)
Amount Detected (ng/sample)
0.04
0.03
0.02
0.01
0.00
LPV Vest
LPV Vulv
Control Vest
Control Vulv
IL-1β
TPPU + IL-1β

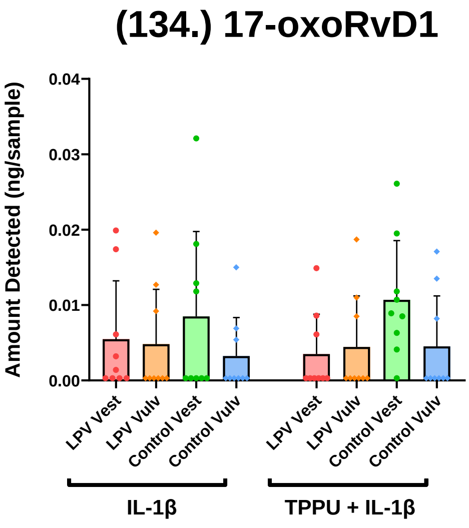
(134.) 17-oxoRvD1
Amount Detected (ng/sample)
0.04
0.03
0.02
0.01
0.00
LPV Vest
LPV Vulv
Control Vest
Control Vulv
IL-1β
TPPU + IL-1β

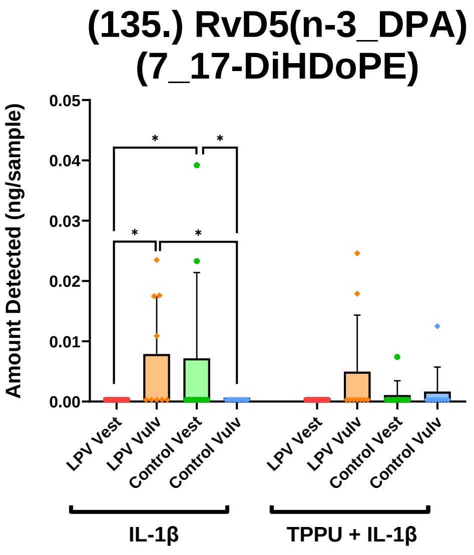
(135.) RvD5(n-3_DPA) (7_17-DiHDoPE)
Amount Detected (ng/sample)
0.05
0.04
0.03
0.02
0.01
0.00
*
*
*
*
LPV Vest
LPV Vulv
Control Vest
Control Vulv
IL-1β
TPPU + IL-1β

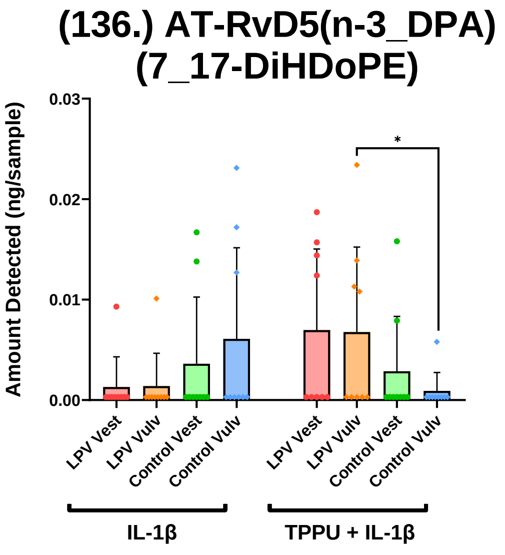
(136.) AT-RvD5(n-3_DPA) (7_17-DiHDoPE)
Amount Detected (ng/sample)
0.03
0.02
0.01
0.00
*
LPV Vest
LPV Vulv
Control Vest
Control Vulv
IL-1β
TPPU + IL-1β

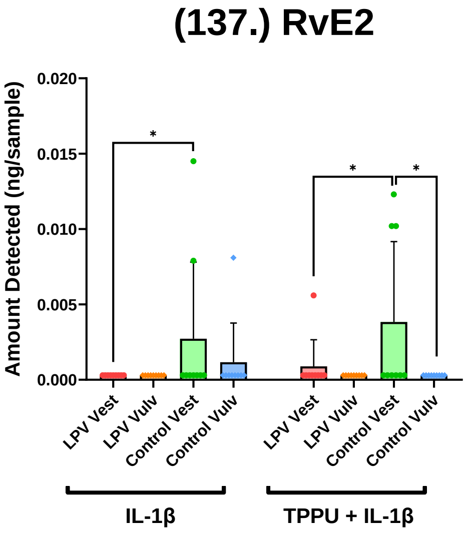
(137.) RvE2
Amount Detected (ng/sample)
0.020
0.015
0.010
0.005
0.000
*
*
*
LPV Vest
LPV Vulv
Control Vest
Control Vulv
IL-1β
TPPU + IL-1β

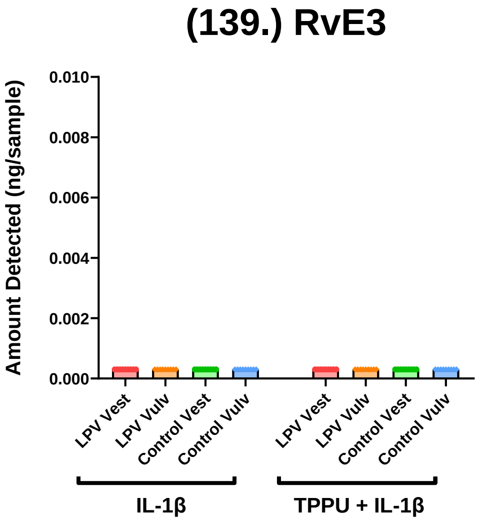
(139.) RvE3
Amount Detected (ng/sample)
0.010
0.008
0.006
0.004
0.002
0.000
LPV Vest
LPV Vulv
Control Vest
Control Vulv
IL-1β
TPPU + IL-1β

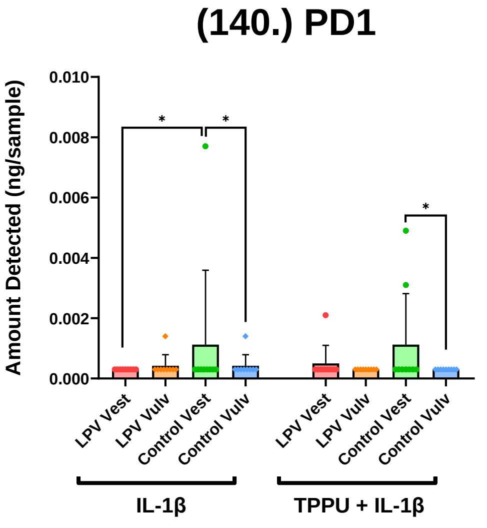
(140.) PD1
Amount Detected (ng/sample)
0.010
0.008
0.006
0.004
0.002
0.000
*
*
*
LPV Vest
LPV Vulv
Control Vest
Control Vulv
IL-1β
TPPU + IL-1β

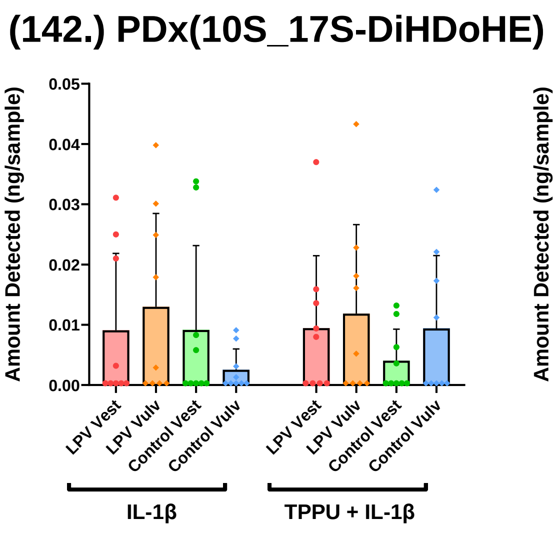

(142.) PDx(10S_17S-DiHDoHE)
Amount Detected (ng/sample)
0.05
0.04
0.03
0.02
0.01
0.00
LPV Vest
LPV Vulv
Control Vest
Control Vulv
LPV Vest
LPV Vulv
Control Vest
Control Vulv
IL-1β
TPPU + IL-1β

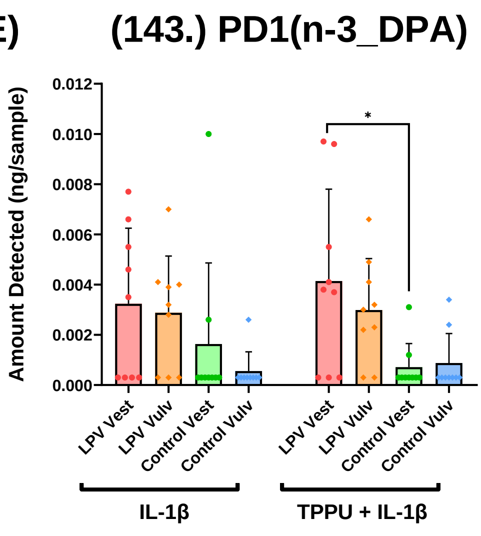

(143.) PD1(n-3_DPA)
Amount Detected (ng/sample)
0.012
0.010
0.008
0.006
0.004
0.002
0.000
*
LPV Vest
LPV Vulv
Control Vest
Control Vulv
LPV Vest
LPV Vulv
Control Vest
Control Vulv
IL-1β
TPPU + IL-1β

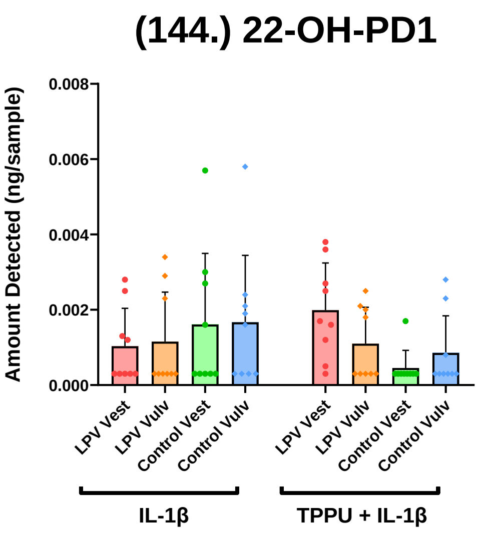

(144.) 22-OH-PD1
Amount Detected (ng/sample)
0.008
0.006
0.004
0.002
0.000
LPV Vest
LPV Vulv
Control Vest
Control Vulv
LPV Vest
LPV Vulv
Control Vest
Control Vulv
IL-1β
TPPU + IL-1β

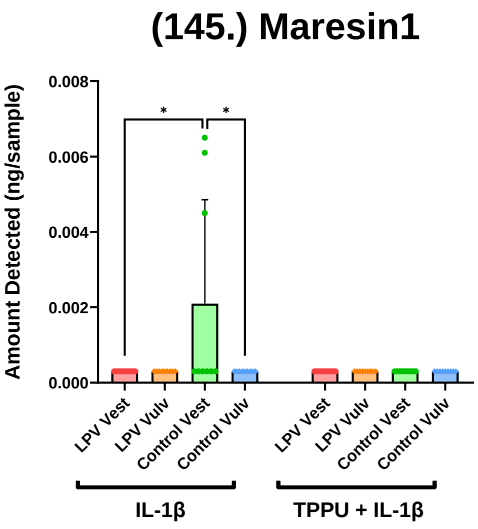

(145.) Maresin1
Amount Detected (ng/sample)
0.008
0.006
0.004
0.002
0.000
*
*
LPV Vest
LPV Vulv
Control Vest
Control Vulv
LPV Vest
LPV Vulv
Control Vest
Control Vulv
IL-1β
TPPU + IL-1β

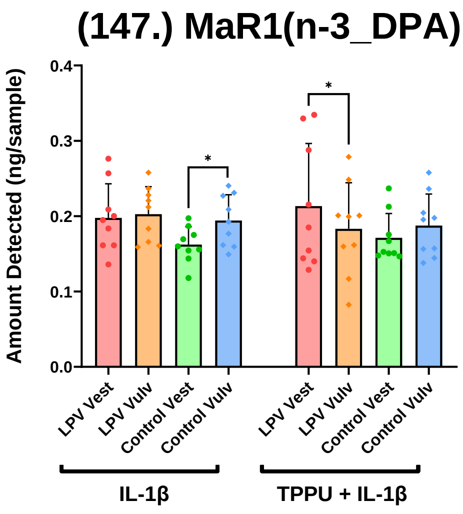

(147.) MaR1(n-3_DPA)
Amount Detected (ng/sample)
0.4
0.3
0.2
0.1
0.0
*
*
LPV Vest
LPV Vulv
Control Vest
Control Vulv
LPV Vest
LPV Vulv
Control Vest
Control Vulv
IL-1β
TPPU + IL-1β

Figure S2. sEH inhibitor treatment at varying doses did not affect cell viability.
An alamarBlue cell viability reagent (Invitrogen; Waltham, MA) was used to ensure that the chosen doses of each inhibitor were not toxic to cells. Treatment with vehicle (DMSO), 500 pg/mL IL-1β, (A) 250 pM and 1 nM GSK2256294, as well as (B) 5 nM and 10 nM EC5026, did not affect cell viability compared to vehicle treatment. The data are expressed as mean ± SEM; n=12.

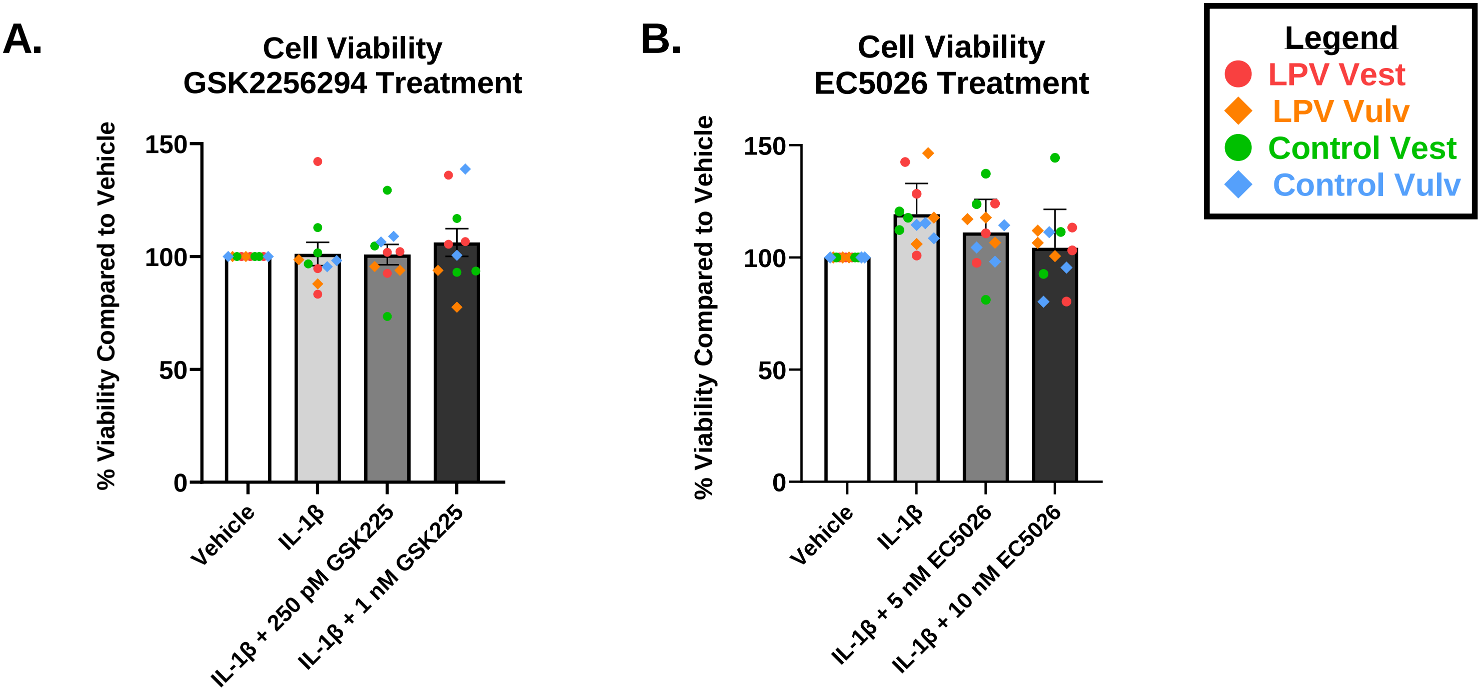

Figure S3. Individual bar graphs of 17-plex Luminex dataset.
The data was collected in duplicates and are expressed as mean ± SEM; n=6; *P < 0.05. 5 out of 17 cytokines were below the assay's limit of detection and not plotted on a graph.

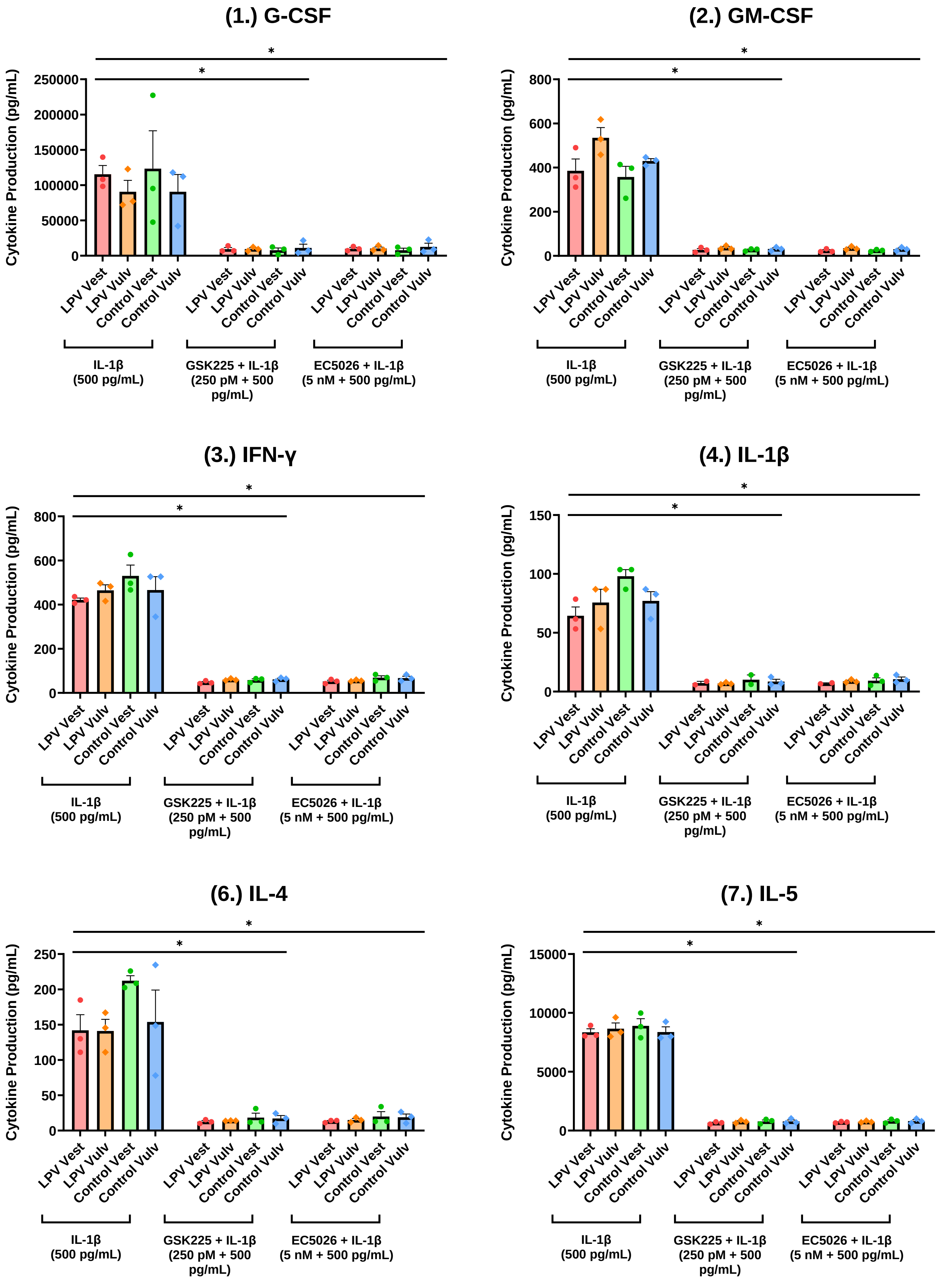

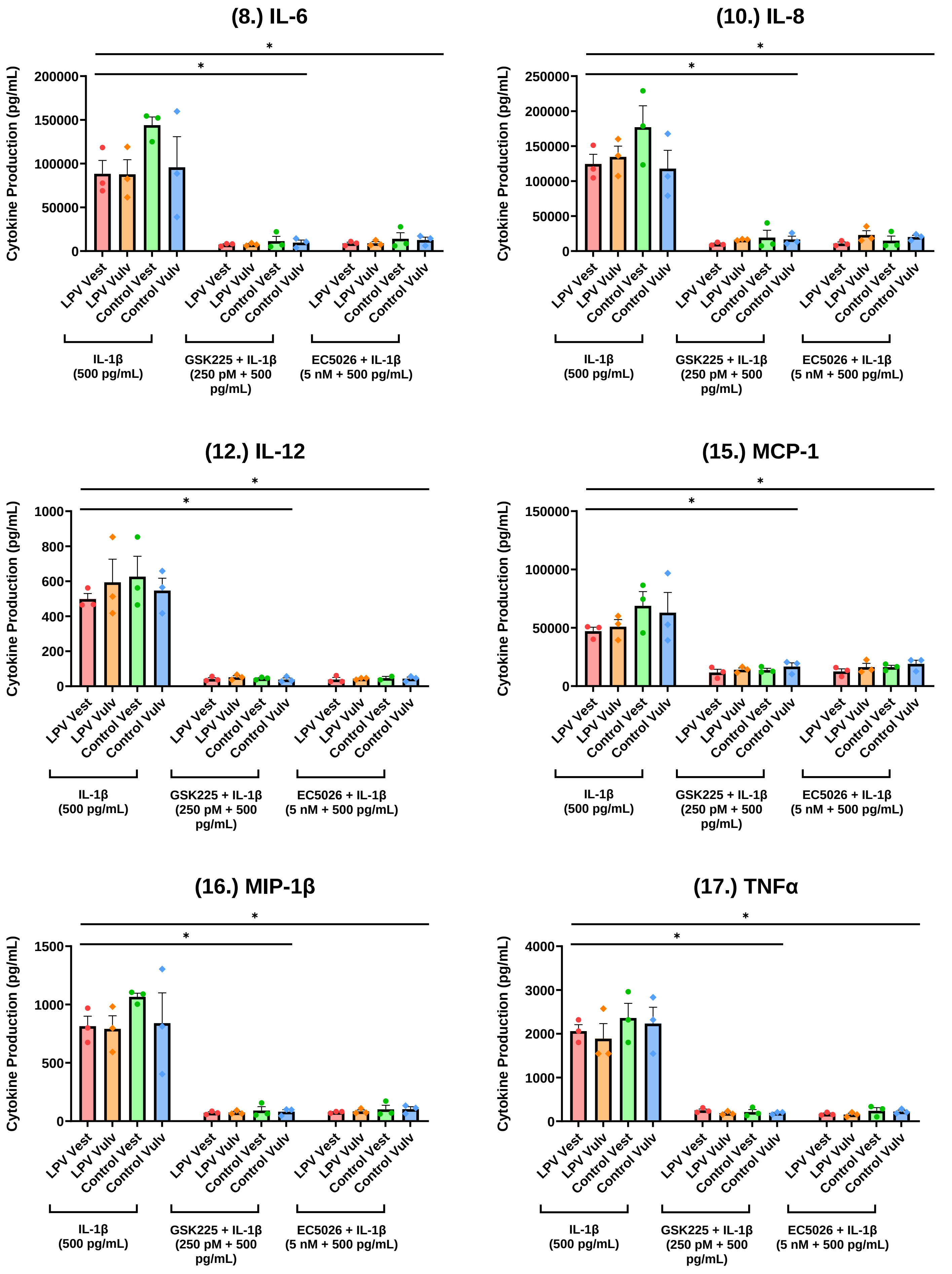
(8.) IL-6
(10.) IL-8
(12.) IL-12
(15.) MCP-1
(16.) MIP-1β
(17.) TNFα
Cytokine Production (pg/mL)
*
LPV Vest
LPV Vulv
Control Vest
Control Vulv
IL-1β
(500 pg/mL)
GSK225 + IL-1β
(250 pM + 500
pg/mL)
EC5026 + IL-1β
(5 nM + 500 pg/mL)

Figure S4. 5,6-EET acts on other calcium channels besides TRPV4 and TRPA1 in vulvar fibroblasts. Stimulation with 5,6-EET following overnight treatment with the TRPV4 selective inhibitor HC-067047 in combination with the TRPA1 selective inhibitor HC-030031 does not attenuate calcium flux in LPV and control fibroblasts. The data was collected in quadruplicates and are expressed as mean ± SEM; n=6.

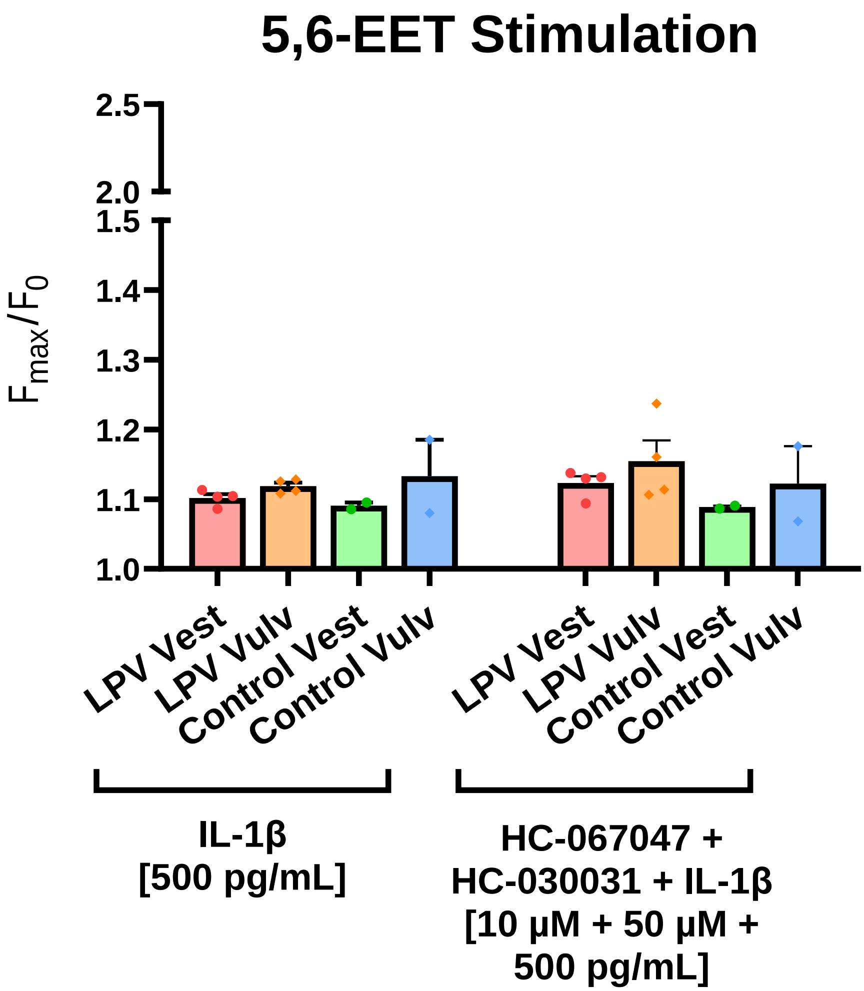

Table S1. *Patient Demographic Information.*

Strain ID	Sample Type	Race	Ethnicity	Age (years)	Vest Threshold	Vulv Threshold
LPV 004	Control	White	Not Hispanic	41	2.64	3.50
LPV 008	Control	White	Not Hispanic	26	1.95	3.87
LPV 009	Case	White	Not Hispanic	22	0.73	1.37
LPV 010	Control	White	Not Hispanic	36	1.80	4.00
LPV 013	Case	White	Not Hispanic	20	0.53	1.13
LPV 014	Case	White	Not Hispanic	61	0.63	1.15
LPV 017	Control	White	Not Hispanic	31	1.62	6.58
LPV 019	Case	White	Not Hispanic	31	0.92	6.82
LPV 020	Case	White	Not Hispanic	25	3.27	0.7
LPV 021	Control	White	Not Hispanic	50	2.50	5.00
LPV 022	Case	White	Not Hispanic	22	0.68	4.52
LPV 023	Control	White	Not Hispanic	20	4.50	4.50
LPV 228	Case	White	Not Hispanic	26	NA	NA
LPV 232	Control	White	Not Hispanic	45	1.97	1.23
LPV 233	Case	White	Not Hispanic	45	0.01	0.91
LPV 236	Control	White	Not Hispanic	34	1.99	4.87
LPV 237	Case	White	Not Hispanic	25	0.93	2.61
LPV 239	Case	White	Not Hispanic	27	0.47	2.08
LPV 246	Case	White	Not Hispanic	31	0.64	2.80

Vest = Vestibule; Vulv = External Vulva; Threshold is measured in grams force.

To view which strains were used for each experiment, please refer to the full dataset that was uploaded to Zenodo. DOI: 10.5281/zenodo.17296455

Table S2. Full Lipidomic Dataset

Lipid amounts in ng listed by patient, broken into treatments, and cases/controls. CO = control; CA = case

Lipid Name	CO 10 VEST IL-1β 1	CO 10 VEST IL-1β 2	CO 10 VEST IL-1β 3	CO 21 VEST IL-1β 1	CO 21 VEST IL-1β 2	CO 21 VEST IL-1β 3	CO 23 VEST IL-1β 1	CO 23 VEST IL-1β 2
13_14-dh-PGE1	0.0003	0.0003	0.0003	0.0008	0.0003	0.0003	0.0003	0.0003
13_14-dh-15k-PGE1	0.0055	0.0181	0.0003	0.0003	0.0003	0.0003	0.0003	0.0003
D17-PGE1	0.0106	0.0003	0.0163	0.0003	0.0003	0.0003	0.0003	0.0003
PGE1	0.0003	0.0003	0.0103	0.0003	0.0003	0.0003	0.0003	0.0003
15(R)-PGE1	0.2187	0.1625	0.1401	0.6518	0.6463	0.5302	2.6126	2.4525
15-keto PGE1	0.0085	0.0003	0.0003	0.0003	0.0076	0.0066	0.0003	0.0194
Bicyclo PGE1	0.0003	0.0003	0.0003	0.0003	0.0003	0.0003	0.0003	0.0003
19(R)-hydroxy PGE1	0.0003	0.0003	0.0003	0.0003	0.0003	0.0003	0.0003	0.0003
2_3-dinor PGE1	0.0003	0.0003	0.0003	0.0003	0.0003	0.0003	0.0003	0.0003
PGE2	20.4033	14.2427	13.9347	61.2211	61.5430	46.0213	156.6527	156.7618
15-keto PGE2	0.0003	0.0003	0.0003	0.0321	0.0372	0.0215	0.1233	0.1245
13_14-dh-15k-PGE2	0.0003	0.0284	0.0003	0.1074	0.0003	0.0767	0.4117	0.4057
Bicyclo PGE2	0.0003	0.0003	0.0498	0.0003	0.0003	0.0200	0.0003	0.0003
PGA2	0.0003	0.0003	0.0003	0.0003	0.0003	0.0003	0.0003	0.0003
19(R)-OH PGE2 & 20-OH PGE2	0.0003	0.0003	0.0003	0.0003	0.0012	0.0003	0.0003	0.0061
tetranor PGEM	0.0003	0.0003	0.0003	0.0003	0.0305	0.0003	0.0305	0.0003
PGE3	0.6651	0.4304	0.4136	1.6575	1.7427	1.3389	6.4792	5.6585
PGD2	18.5425	14.0015	13.3487	65.1495	57.0091	43.3809	152.8566	155.4893
PGJ2	0.0003	0.0003	0.0003	0.0003	0.0003	0.0003	0.0003	0.0003
D12-PGJ2	0.0003	0.0003	0.0003	0.0003	0.0003	0.0003	0.1365	0.0003
15d-D12_14-PGJ2	0.0003	0.0003	0.0003	0.0003	0.0003	0.0003	0.0003	0.0003
13_14-dh-15k-PGD2	0.0003	0.0003	0.0003	0.0003	0.0003	0.0003	0.0003	0.0003
PGD3	0.0003	0.0003	0.0003	0.0003	0.0003	0.0003	0.0003	0.0003
15d-D12_14-PGJ3	0.0003	0.0003	0.0003	0.0003	0.0003	0.0003	0.0003	0.0003
PGF1a	0.0003	0.0003	0.0003	0.0421	0.0407	0.0237	0.0003	0.1482
PGF2a	0.0357	0.0003	0.0280	0.0003	0.1074	0.0003	0.0003	0.0003
15-keto PGF2a	0.0003	0.0003	0.0003	0.0003	0.0003	0.0003	0.0003	0.0003
13_14-dh-15k-PGF2a	0.0003	0.0003	0.0003	0.0003	0.0003	0.0003	0.0003	0.0003
19(R)-OH PGF2a & 20-OH PGF2a	0.0003	0.0003	0.0003	0.0003	0.0003	0.0003	0.0003	0.0003
PGF3a	0.1116	0.0003	0.0003	0.0003	0.0003	0.0003	0.0212	0.0003
8-isoPGF2a & 11bPGF2a	0.0003	0.0003	0.0003	0.0003	0.0003	0.0003	0.0003	0.0003
iPF-VI	0.0003	0.0003	0.0003	0.0003	0.0003	0.0003	0.0003	0.0003
6-keto PGF1a	0.0003	0.0162	0.0003	0.0520	0.0661	0.0451	0.0003	0.0003
6-keto PGE1	0.0003	0.0003	0.0003	0.0003	0.0003	0.0003	0.0003	0.0104
6_15-diketo PGFa	0.0003	0.0003	0.0003	0.0003	0.0003	0.0003	0.0003	0.0003
TXB2	0.0003	0.0003	0.0003	0.0080	0.0003	0.0003	0.0003	0.0003
11-dh-TXB2	0.0038	0.0216	0.0003	0.0003	0.0003	0.0003	0.0003	0.0095
2_3-dinor TXB2	0.0300	0.0312	0.0490	0.0517	0.0391	0.0412	0.0560	0.0541
11-dh-2_3-dinor TXB2	0.0003	0.0076	0.0003	0.0003	0.0003	0.0003	0.0003	0.0003
TXB3	0.0003	0.0003	0.0003	0.0003	0.0003	0.0003	0.0003	0.0003
11-dh TXB3	0.0003	0.0080	0.0003	0.0032	0.0003	0.0003	0.0003	0.0057
LTB4	0.0104	0.0003	0.0053	0.0003	0.0003	0.0003	0.0003	0.0003
12-OxoLTB4	0.0003	0.0003	0.0003	0.0003	0.0003	0.0003	0.0003	0.0003
20-hydroxy LTB4	0.0231	0.0101	0.0087	0.0067	0.0051	0.0037	0.0107	0.0130
20-COOH LTB4	0.0079	0.0074	0.0113	0.0098	0.0082	0.0029	0.0056	0.0178
18-carboxy dinor LTB4	0.0003	0.0003	0.0003	0.0003	0.0003	0.0003	0.0003	0.0003
LTB5	0.0003	0.0003	0.0003	0.0003	0.0003	0.0003	0.0003	0.0003
5(S)_6(S)-DiHETE	0.0003	0.0003	0.0003	0.0003	0.0003	0.0003	0.0003	0.0003
5(S)_12(S)-DiHETE	0.0003	0.0033	0.0064	0.0003	0.0171	0.0003	0.0175	0.0003

5(S)_15(S)-DiHETE	0.0003	0.0003	0.0003	0.0003	0.0003	0.0003	0.0003	0.0003
8(S)_15(S)-DiHETE	0.0003	0.0003	0.0003	0.0003	0.0003	0.0003	0.0003	0.0003
5(S)_15(S)-DiHEPE	0.0003	0.0003	0.0003	0.0003	0.0003	0.0003	0.0003	0.0003
9-HODE	1.7437	0.0003	0.0003	1.7193	1.6405	1.2034	2.0705	1.9114
13-HODE	0.0003	0.0003	0.0003	0.0003	0.0003	0.0003	0.0003	0.0003
9-HOTrE	0.0003	0.0003	0.0003	0.0003	0.0003	0.0003	0.0003	0.0003
13-HOTrE	0.0003	0.0003	0.0003	0.0003	0.0003	0.0003	0.0003	0.0003
13-HOTrE(g)	0.0003	0.0003	0.0003	0.4190	0.3343	0.0003	0.2643	0.0003
11-HEDE	0.1500	0.0003	0.0003	0.0003	0.0161	0.0003	0.0465	0.0311
15-HEDE	0.0003	0.0003	0.0003	0.0003	0.0003	0.0003	0.0003	0.0003
8-HETrE	0.0003	0.0003	0.0003	0.0003	0.0003	0.0003	0.0003	0.0003
5-HETrE	0.0003	0.0003	0.0003	0.0003	0.0003	0.0003	0.0003	0.0003
5-HETE	0.0438	0.1165	0.0798	0.2494	0.2553	0.2075	0.3826	0.3433
8-HETE	0.0003	0.0003	0.0003	0.0003	0.0003	0.0003	0.0003	0.0003
9-HETE	0.1979	0.1426	0.0596	0.1568	0.1895	0.1195	0.5690	0.5065
11-HETE	0.0003	0.0003	0.0003	0.0003	0.0003	0.0003	0.0003	0.0003
12-HETE	0.1080	0.0735	0.1069	0.1611	0.2856	0.2573	0.4035	0.3794
15-HETE	0.0003	0.0003	0.0003	0.0003	0.0003	0.0003	0.0003	0.0003
20-HETE	0.0003	0.0524	0.0003	0.0003	0.0003	0.0003	0.0003	0.0003
tetranor 12-HETE	0.0003	0.0618	0.0003	0.0717	0.0797	0.0659	0.1699	0.1198
12-HHTrE	0.0003	0.0003	0.0003	0.0003	0.0003	0.0003	0.0003	0.0173
5-HEPE	0.0003	0.0524	0.0003	0.0003	0.0003	0.0003	0.0003	0.0003
8-HEPE	0.0003	0.0003	0.0003	0.0003	0.0003	0.0003	0.0003	0.0003
9-HEPE	0.0003	0.0003	0.0003	0.0003	0.0003	0.0249	0.0003	0.0003
11-HEPE	0.0003	0.0003	0.0003	0.0003	0.0003	0.0003	0.0003	0.0003
12-HEPE	0.0003	0.0003	0.0003	0.0003	0.0003	0.0003	0.0237	0.0003
15-HEPE	0.0003	0.0003	0.0003	0.0003	0.0003	0.0003	0.0003	0.0003
18-HEPE	0.0003	0.0003	0.0003	0.0003	0.0003	0.0003	0.0003	0.0003
4-HDoHE	0.0003	0.0003	0.0003	0.0003	0.0003	0.0003	0.0003	0.0003
7-HDoHE	0.0436	0.0003	0.0056	0.0144	0.0037	0.0003	0.0130	0.0091
8-HDoHE	0.0893	0.0321	0.0636	0.0003	0.0003	0.0806	0.1372	0.0003
10-HDoHE	0.0209	0.0003	0.0003	0.0003	0.0003	0.0003	0.0178	0.0003
11-HDoHE	0.0213	0.0003	0.0003	0.0003	0.0003	0.0003	0.0003	0.0003
13-HDoHE	0.0003	0.0003	0.0003	0.0003	0.0003	0.0003	0.0003	0.0003
14-HDoHE	0.0193	0.0073	0.0057	0.0066	0.0003	0.0003	0.0003	0.0003
16-HDoHE	0.1666	0.1046	0.1795	0.0742	0.1709	0.0939	0.1327	0.1459
17-HDoHE	0.0003	0.0003	0.0003	0.0003	0.0003	0.0003	0.0003	0.0003
20-HDoHE	0.3693	0.2156	0.3552	0.1849	0.2085	0.1507	0.2203	0.2165
9(10)-EpOME	0.0003	0.0003	0.0003	0.0586	0.0003	0.0345	0.0488	0.0003
12(13)-EpOME	1.1129	0.0003	0.0003	0.0003	0.4956	0.0003	0.6100	0.5630
5(6)-EpETrE	0.0003	0.0003	0.0025	0.0003	0.0003	0.0003	0.0003	0.0003
8(9)-EpETrE	0.0003	0.0003	0.0003	0.0592	0.0343	0.0300	0.0770	0.0765
11(12)-EpETrE	3.2913	3.0787	3.0412	9.4335	8.8169	7.6357	26.1205	23.3259
14(15)-EpETrE	0.6325	0.6208	0.5469	1.7694	1.9703	1.5017	5.8506	5.0174
8(9)-EpETE	0.0003	0.0003	0.0003	0.0003	0.0003	0.0003	0.0003	0.0003
11(12)-EpETE	0.2875	0.1962	0.2136	0.5728	0.6481	0.4870	1.4392	1.3546
14(15)-EpETE	0.0643	0.0533	0.0368	0.0996	0.1274	0.0726	0.2591	0.2792
17(18)-EpETE	0.0003	0.0003	0.0003	0.0003	0.0003	0.0003	0.0003	0.0003
7(8)-EpDPE	0.0003	0.0003	0.0003	0.0003	0.0003	0.0104	0.0003	0.0003
10(11)-EpDPE	0.0184	0.0003	0.0121	0.0003	0.0304	0.0003	0.0003	0.0003
13(14)-EpDPE	0.0003	0.0003	0.0003	0.0003	0.0003	0.0003	0.0003	0.0003
16(17)-EpDPE	0.0003	0.0003	0.0003	0.0003	0.0003	0.0003	0.0003	0.0003
19(20)-EpDPE	0.0553	0.0314	0.0416	0.0891	0.1203	0.0787	0.1653	0.1677

9_10-DiHOME	0.0003	0.0003	0.0003	0.0003	0.0003	0.0003	0.0003	0.0003
12_13-DiHOME	0.0003	0.0003	0.0003	0.0003	0.0003	0.0003	0.0003	0.0003
5_6-DiHETrE	0.0003	0.0003	0.0003	0.0003	0.0003	0.0003	0.0003	0.0003
8_9-DiHETrE	0.0003	0.0003	0.0003	0.0003	0.0003	0.0003	0.0003	0.0083
11_12-DiHETrE	0.0200	0.0003	0.0133	0.0254	0.0169	0.0208	0.0286	0.0153
14_15-DiHETrE	0.0236	0.0205	0.0126	0.0575	0.0563	0.0559	0.0628	0.0543
5_6-DiHETE(EPA)	0.0003	0.0003	0.0003	0.0003	0.0003	0.0003	0.0003	0.0003
19_20-DiHDoPE	0.0003	0.0003	0.0003	0.0003	0.0003	0.0003	0.0003	0.0003
9-OxoODE	0.0820	0.0586	0.0327	0.0003	0.0003	0.0003	0.0003	0.0283
13-OxoODE	0.0003	0.0003	0.0003	0.0092	0.0003	0.0003	0.0003	0.0003
9-OxoOTrE	0.0003	0.0003	0.0003	0.0003	0.0003	0.0003	0.0003	0.0003
15-OxoEDE	0.0003	0.0003	0.0132	0.0280	0.0240	0.0197	0.0321	0.0188
5-oxoETE	0.0003	0.0003	0.0003	0.0003	0.0003	0.0003	0.0003	0.0003
12-OxoETE	0.0003	0.0003	0.0003	0.0003	0.0003	0.0003	0.0003	0.0003
15-OxoETE	0.0003	0.0003	0.0003	0.0003	0.0003	0.0003	0.0466	0.0003
LXA4	0.0003	0.0003	0.0003	0.0003	0.0003	0.0003	0.0003	0.0003
15-epi LXA4	0.0003	0.0003	0.0003	0.0003	0.0003	0.0003	0.0003	0.0003
15-oxo LXA4	0.0003	0.0003	0.0003	0.0003	0.0003	0.0003	0.0003	0.0003
LXA5	0.0003	0.0003	0.0003	0.0003	0.0740	0.0003	0.0003	0.0003
LXB4	0.2025	0.1890	0.1876	0.8203	0.6513	0.5908	3.2696	3.0775
AT-LXB4	0.0003	0.0003	0.0003	0.0238	0.0003	0.0003	0.0419	0.0003
RvD1 & AT-RvD1	0.0340	0.0003	0.0444	0.0244	0.0003	0.0003	0.0003	0.0291
RvD2	0.1058	0.0003	0.0003	0.0003	0.0003	0.0003	0.0003	0.0003
RvD3	0.0003	0.0003	0.0003	0.0003	0.0003	0.0003	0.0003	0.0003
AT-RvD3	0.0003	0.0003	0.0003	0.0003	0.0003	0.0003	0.0003	0.0003
RvD4	0.0003	0.0003	0.0003	0.0003	0.0003	0.0003	0.0003	0.0003
AT-RvD4	0.0003	0.0003	0.0003	0.0003	0.0003	0.0003	0.0003	0.0003
RvD5	0.0003	0.0003	0.0003	0.0003	0.0680	0.0003	0.0003	0.0003
RvD6 (4_17-DiHDoHE)	0.0196	0.0168	0.0003	0.0003	0.0003	0.0003	0.0003	0.0323
AT-RvD6 (4_17-DiHDoHE)	0.0003	0.0003	0.0003	0.0003	0.0003	0.0003	0.0003	0.0003
8-oxoRvD1	0.0003	0.0003	0.0003	0.0003	0.0003	0.0003	0.0003	0.0003
17-oxoRvD1	0.0181	0.0321	0.0003	0.0003	0.0118	0.0003	0.0003	0.0129
RvD5(n-3_DPA) (7_17-DiHDoPE)	0.0233	0.0003	0.0003	0.0003	0.0003	0.0003	0.0392	0.0003
AT-RvD5(n-3_DPA) (7_17-DiHDoPE)	0.0003	0.0138	0.0167	0.0003	0.0003	0.0003	0.0003	0.0003
RvE1	0.0003	0.0003	0.0003	0.0003	0.0003	0.0003	0.0003	0.0003
RvE2	0.0003	0.0003	0.0003	0.0003	0.0003	0.0003	0.0145	0.0003
RvE3	0.0003	0.0003	0.0003	0.0003	0.0003	0.0003	0.0003	0.0003
PD1	0.0003	0.0003	0.0003	0.0003	0.0003	0.0003	0.0003	0.0003
AT-PD1	0.0003	0.0003	0.0003	0.0003	0.0003	0.0003	0.0003	0.0003
PDx(10S_17S-DiHDoHE)	0.0003	0.0328	0.0003	0.0058	0.0083	0.0003	0.0003	0.0003
PD1(n-3_DPA)	0.0100	0.0026	0.0003	0.0003	0.0003	0.0003	0.0003	0.0003
22-OH-PD1	0.0057	0.0003	0.0027	0.0003	0.0016	0.0030	0.0003	0.0003
Maresin1	0.0003	0.0003	0.0003	0.0003	0.0003	0.0003	0.0045	0.0065
Maresin2	0.0003	0.0003	0.0003	0.0003	0.0003	0.0003	0.0003	0.0003
MaR1(n-3_DPA)	0.1179	0.1599	0.1438	0.1751	0.1868	0.1558	0.1972	0.1695

CO 23 VEST IL-1β 3	CO 10 VULV IL-1β 1	CO 10 VULV IL-1β 2	CO 10 VULV IL-1β 3	CA 20 VULV IL-1β 1	CA 20 VULV IL-1β 2	CA 20 VULV IL-1β 3	CO 21 VULV IL-1β 1	CO 21 VULV IL-1β 2	CO 21 VULV IL-1β 3
0.0003	0.0003	0.0084	0.0003	0.0003	0.0003	0.0003	0.0003	0.0003	0.0003
0.0003	0.0003	0.0003	0.0096	0.0003	0.0003	0.0003	0.0003	0.0039	0.0003
0.0003	0.0003	0.0125	0.0003	0.0003	0.0003	0.0003	0.0003	0.0003	0.0003
0.3865	0.0096	0.0204	0.0039	0.0003	0.0003	0.0003	0.0003	0.0003	0.0003
2.4485	0.0795	0.0584	0.1497	0.2373	0.3778	0.3086	0.3788	0.3385	0.3952
0.0080	0.0118	0.0213	0.0003	0.0003	0.0044	0.0003	0.0003	0.0003	0.0091
0.0003	0.0003	0.0003	0.0003	0.0003	0.0003	0.0003	0.0003	0.0003	0.0003
0.0003	0.0003	0.0003	0.0003	0.0003	0.0003	0.0003	0.0003	0.0003	0.0003
0.0003	0.0003	0.0003	0.0003	0.0003	0.0003	0.0003	0.0003	0.0003	0.0003
135.8531	7.0864	5.8185	5.5866	50.7996	34.2916	32.6023	36.1707	31.6139	37.9984
0.1150	0.0003	0.0003	0.0003	0.0400	0.0339	0.0003	0.0222	0.0236	0.0346
0.3927	0.0137	0.0217	0.0003	0.0003	0.0003	0.0526	0.0003	0.0003	0.0518
0.0003	0.0003	0.0003	0.0003	0.0003	0.0003	0.0003	0.0471	0.0003	0.0003
0.0003	0.0003	0.0003	0.0003	0.0003	0.0003	0.0003	0.0003	0.0003	0.0003
0.0003	0.0003	0.0003	0.0003	0.0003	0.0003	0.0003	0.0026	0.0015	0.0036
0.0003	0.0003	0.0003	0.0003	0.0003	0.0173	0.0003	0.0003	0.0003	0.0003
5.2962	0.0924	0.0870	0.0814	1.3383	0.9991	0.8200	1.3714	1.1233	1.6168
132.1566	6.1897	6.1639	5.7309	49.4464	33.9235	28.8007	35.5035	32.2200	38.3253
0.0003	0.0003	0.0003	0.0003	0.0003	0.0003	0.0003	0.0003	0.0003	0.0003
0.0003	0.0003	0.0003	0.0003	0.0003	0.0254	0.0189	0.0003	0.0003	0.0003
0.0003	0.0003	0.0003	0.0003	0.0003	0.0003	0.0003	0.0003	0.0003	0.0003
0.0003	0.0003	0.0003	0.0003	0.0003	0.0003	0.0003	0.0003	0.0003	0.0003
0.0003	0.0003	0.0003	0.0003	0.0003	0.0003	0.0003	0.0003	0.0003	0.0003
0.0003	0.0003	0.0003	0.0003	0.0003	0.0003	0.0003	0.0003	0.0003	0.0003
0.1306	0.0003	0.0003	0.0003	0.0292	0.0177	0.0232	0.0245	0.0003	0.0223
0.0003	0.0233	0.0003	0.0003	0.0003	0.0003	0.0003	0.0003	0.0003	0.0003
0.4780	0.0003	0.0003	0.0075	0.0003	0.0003	0.0003	0.0003	0.0610	0.0003
0.0003	0.0003	0.0003	0.0003	0.0003	0.0003	0.0003	0.0003	0.0003	0.0003
0.0003	0.0003	0.0003	0.0003	0.0003	0.0003	0.0003	0.0003	0.0003	0.0003
0.0243	0.0421	0.0003	0.0003	0.0003	0.0003	0.0003	0.0003	0.0003	0.0003
0.0003	0.0054	0.0003	0.0003	0.0003	0.0003	0.0003	0.0037	0.0003	0.0056
0.0342	0.0003	0.0003	0.0003	0.0104	0.0003	0.0003	0.0003	0.0003	0.0003
0.0716	0.0445	0.0003	0.0503	0.4628	0.0003	0.0003	0.0003	0.0003	0.4289
0.0003	0.0003	0.0003	0.0003	0.0003	0.0075	0.0003	0.0003	0.0003	0.0003
0.0003	0.0003	0.0003	0.0003	0.0003	0.0003	0.0003	0.0003	0.0031	0.0030
0.0003	0.0003	0.0003	0.0003	0.0003	0.0098	0.0003	0.0003	0.0003	0.0003
0.0003	0.0003	0.0003	0.0003	0.0003	0.0070	0.0003	0.0042	0.0036	0.0003
0.0609	0.0302	0.0418	0.0320	0.0566	0.0352	0.0003	0.0607	0.0493	0.0003
0.0003	0.0037	0.0003	0.0003	0.0003	0.0003	0.0003	0.0003	0.0003	0.0003
0.0003	0.0003	0.0003	0.0003	0.0003	0.0003	0.0003	0.0003	0.0003	0.0003
0.0003	0.0003	0.0003	0.0003	0.0003	0.0003	0.0003	0.0003	0.0003	0.0003
0.0088	0.0090	0.0003	0.0003	0.0059	0.0082	0.0075	0.0003	0.0003	0.0060
0.0003	0.0003	0.0003	0.0003	0.0003	0.0003	0.0003	0.0003	0.0003	0.0003
0.0059	0.0132	0.0008	0.0134	0.0077	0.0156	0.0074	0.0058	0.0078	0.0090
0.0135	0.0105	0.0003	0.0112	0.0097	0.0113	0.0081	0.0164	0.0126	0.0137
0.0003	0.0003	0.0003	0.0003	0.0003	0.0003	0.0003	0.0003	0.0003	0.0003
0.0003	0.0003	0.0003	0.0003	0.0003	0.0003	0.0003	0.0003	0.0003	0.0003
0.0003	0.0003	0.0003	0.0003	0.0003	0.0003	0.0003	0.0003	0.0003	0.0003
0.0003	0.0102	0.0082	0.0099	0.0003	0.0077	0.0003	0.0047	0.0197	0.0035

0.0003	0.0003	0.0003	0.0003	0.0003	0.0003	0.0003	0.0003	0.0003	0.0003
0.0003	0.0003	0.0003	0.0003	0.0003	0.0003	0.0356	0.0003	0.0003	0.0003
0.0003	0.0003	0.0003	0.0003	0.0003	0.0003	0.0003	0.0003	0.0003	0.0003
1.4445	0.0003	0.0003	0.9646	2.8275	2.3511	1.3230	1.2150	0.9986	1.3730
0.0003	0.0003	0.0003	0.0003	0.0003	0.0003	0.0003	0.0003	0.0003	0.0003
0.0003	0.0003	0.0003	0.0003	0.0003	0.0003	0.0003	0.0003	0.0003	0.0003
0.0003	0.0591	0.0003	0.0003	0.0003	0.0003	0.0003	0.0003	0.0003	0.0003
0.0003	0.0003	0.0003	0.0003	0.0003	0.0003	0.0003	0.6588	0.5300	0.4264
0.0003	0.1091	0.0003	0.0003	0.0003	0.0003	0.0003	0.0003	0.0229	0.0003
0.0003	0.0003	0.0003	0.0003	0.0003	0.0003	0.0003	0.0003	0.0003	0.0003
0.0003	0.0003	0.0003	0.0003	0.0003	0.0003	0.0003	0.0003	0.0003	0.0003
0.0003	0.0003	0.0003	0.0003	0.0003	0.0003	0.0003	0.0003	0.0003	0.0003
0.2910	0.0924	0.0839	0.0950	0.0962	0.0921	0.1154	0.1867	0.1436	0.2082
0.0270	0.0003	0.0003	0.0003	0.0003	0.0003	0.0003	0.0003	0.0003	0.0003
0.3952	0.1717	0.0393	0.0232	0.1374	0.1938	0.1122	0.1526	0.0982	0.1426
0.0003	0.0003	0.0003	0.0003	0.0003	0.0003	0.0003	0.0003	0.0003	0.0003
0.3360	0.0958	0.0912	0.0795	0.1342	0.0817	0.1154	0.2371	0.2375	0.1907
0.0003	0.0003	0.0003	0.0003	0.0003	0.0003	0.0003	0.0003	0.0003	0.0003
0.0003	0.0003	0.0003	0.0003	0.0003	0.0003	0.0003	0.0003	0.0003	0.0003
0.1349	0.0003	0.0003	0.0003	0.0345	0.0378	0.0396	0.0592	0.0537	0.0648
0.0003	0.0003	0.0003	0.0003	0.0003	0.0003	0.0003	0.0003	0.0003	0.0183
0.0003	0.0003	0.0003	0.0003	0.0003	0.0003	0.0003	0.0003	0.0003	0.0003
0.0003	0.0003	0.0003	0.0003	0.0003	0.0003	0.0338	0.0003	0.0003	0.0003
0.0305	0.0003	0.0003	0.0003	0.0003	0.0003	0.0003	0.0003	0.0003	0.0003
0.0003	0.0003	0.0003	0.0003	0.0003	0.0003	0.0003	0.0003	0.0003	0.0003
0.0003	0.0003	0.0003	0.0003	0.0003	0.0003	0.0003	0.0003	0.0003	0.0003
0.0003	0.0003	0.0003	0.0003	0.0003	0.0003	0.0003	0.0003	0.0003	0.0003
0.0003	0.0003	0.0003	0.0003	0.0003	0.0003	0.0003	0.0003	0.0003	0.0003
0.0003	0.0003	0.0003	0.0003	0.0003	0.0003	0.0003	0.0003	0.0003	0.0003
0.0210	0.0003	0.0003	0.0003	0.0189	0.0003	0.0003	0.0176	0.0003	0.0003
0.0003	0.0003	0.0003	0.0003	0.0544	0.0003	0.0222	0.0964	0.0333	0.0003
0.0221	0.0003	0.0268	0.0254	0.0003	0.0003	0.0003	0.0003	0.0133	0.0003
0.0003	0.0003	0.0003	0.0003	0.0003	0.0003	0.0003	0.0003	0.0003	0.0003
0.0003	0.0003	0.0003	0.0003	0.0003	0.0003	0.0003	0.0003	0.0003	0.0003
0.0003	0.0071	0.0003	0.0003	0.0003	0.0022	0.0003	0.0003	0.0003	0.0003
0.1844	0.1427	0.1513	0.2416	0.1452	0.1402	0.2067	0.2066	0.1613	0.1343
0.0003	0.0003	0.0003	0.0003	0.0003	0.0003	0.0003	0.0003	0.0003	0.0003
0.2201	0.2424	0.2374	0.3367	0.1647	0.2439	0.3494	0.2284	0.1820	0.2263
0.0003	0.0003	0.0003	0.0003	0.0003	0.0620	0.0515	0.0003	0.0003	0.0003
0.0003	0.0003	0.0003	0.0003	0.0003	1.0805	0.0003	0.0003	0.0003	0.0003
0.0003	0.0003	0.0003	0.0003	0.0003	0.0003	0.0003	0.0003	0.0003	0.0003
0.0571	0.0164	0.0166	0.0119	0.0140	0.0189	0.0305	0.0301	0.0297	0.0309
21.7979	2.6061	2.2983	2.3306	6.3974	5.0142	3.5888	6.3167	6.3030	8.0599
4.8997	0.4262	0.3671	0.3760	1.2644	1.0434	0.8971	1.6340	1.2211	1.5820
0.0003	0.0003	0.0003	0.0003	0.0003	0.0003	0.0003	0.0003	0.0003	0.0003
1.2705	0.0488	0.0355	0.0593	0.5197	0.4134	0.2637	0.4681	0.4519	0.6293
0.1907	0.0103	0.0003	0.0078	0.0711	0.0745	0.0588	0.0813	0.1047	0.1021
0.0003	0.0003	0.0003	0.0003	0.0003	0.0003	0.0003	0.0003	0.0003	0.0003
0.0003	0.0003	0.0003	0.0003	0.0003	0.0003	0.0003	0.0003	0.0003	0.0003
0.0003	0.0003	0.0126	0.0003	0.0003	0.0108	0.0064	0.0003	0.0003	0.0003
0.0003	0.0003	0.0003	0.0003	0.0003	0.0003	0.0003	0.0003	0.0003	0.0003
0.0003	0.0003	0.0003	0.0003	0.0003	0.0003	0.0003	0.0003	0.0003	0.0003
0.1453	0.0275	0.0222	0.0242	0.0408	0.0347	0.0604	0.0778	0.0566	0.0788

0.0003	0.0003	0.0003	0.0003	0.0003	0.0003	0.0003	0.0003	0.0003	0.0003
0.0003	0.0003	0.0003	0.0003	0.0003	0.0003	0.0003	0.0003	0.0003	0.0003
0.0003	0.0003	0.0003	0.0003	0.0003	0.0003	0.0003	0.0003	0.0003	0.0003
0.0053	0.0003	0.0003	0.0003	0.0003	0.0003	0.0003	0.0003	0.0003	0.0003
0.0227	0.0003	0.0003	0.0003	0.0003	0.0003	0.0140	0.0338	0.0124	0.0363
0.0576	0.0113	0.0185	0.0165	0.0390	0.0252	0.0326	0.0608	0.0503	0.0590
0.0003	0.0003	0.0003	0.0003	0.0003	0.0003	0.0003	0.0003	0.0003	0.0003
0.0003	0.0003	0.0003	0.0077	0.0003	0.0003	0.0058	0.0003	0.0003	0.0003
0.0003	0.0003	0.0003	0.0402	0.0003	0.0003	0.0003	0.0399	0.0003	0.0183
0.0003	0.0251	0.0003	0.0003	0.0003	0.0003	0.0003	0.0003	0.0081	0.0003
0.0003	0.0003	0.0003	0.0003	0.0003	0.0003	0.0003	0.0003	0.0003	0.0003
0.0218	0.0101	0.0003	0.0003	0.0117	0.0159	0.0003	0.0186	0.0100	0.0115
0.0003	0.0003	0.0003	0.0003	0.0003	0.0003	0.0003	0.0003	0.0003	0.0003
0.0003	0.0003	0.0003	0.0003	0.0003	0.0003	0.0003	0.0003	0.0003	0.0003
0.0003	0.0003	0.0003	0.0003	0.0003	0.0003	0.0003	0.0003	0.0003	0.0003
0.0003	0.0003	0.0003	0.0003	0.0003	0.0003	0.0003	0.0003	0.0290	0.0003
0.0003	0.0003	0.0273	0.0106	0.0003	0.0003	0.0003	0.0196	0.0003	0.0074
0.0003	0.0003	0.0003	0.0003	0.0003	0.0003	0.0003	0.0003	0.0003	0.0003
0.0003	0.0003	0.0003	0.0003	0.0003	0.0003	0.0003	0.0003	0.0003	0.0003
2.4718	0.1509	0.0902	0.0969	0.6288	0.4663	0.4732	0.4988	0.3970	0.4902
0.0266	0.0003	0.0003	0.0003	0.0003	0.0003	0.0003	0.0003	0.0003	0.0003
0.0302	0.0134	0.0509	0.0158	0.0003	0.0003	0.0201	0.0369	0.0003	0.0003
0.0003	0.1084	0.3765	0.0003	0.0003	0.0003	0.0314	0.0003	0.0003	0.0003
0.0003	0.0003	0.0003	0.0003	0.0003	0.0003	0.0003	0.0003	0.0003	0.0003
0.0003	0.0003	0.0003	0.0003	0.0003	0.0003	0.0003	0.0003	0.0003	0.0003
0.0003	0.0003	0.0003	0.0003	0.0003	0.0003	0.0003	0.0003	0.0003	0.0003
0.0003	0.0003	0.0003	0.0003	0.0003	0.0003	0.0453	0.0003	0.0003	0.0003
0.0003	0.0003	0.0003	0.0003	0.0003	0.0003	0.0003	0.0579	0.0003	0.0003
0.0003	0.0267	0.0003	0.0003	0.0003	0.0003	0.0137	0.0185	0.0170	0.0003
0.0003	0.0003	0.0003	0.0003	0.0003	0.0003	0.0003	0.0003	0.0003	0.0003
0.0003	0.0003	0.0003	0.0003	0.0003	0.0003	0.0003	0.0003	0.0003	0.0003
0.0003	0.0003	0.0150	0.0069	0.0003	0.0127	0.0003	0.0003	0.0003	0.0054
0.0003	0.0003	0.0003	0.0003	0.0003	0.0003	0.0003	0.0003	0.0003	0.0003
0.0003	0.0231	0.0003	0.0172	0.0003	0.0003	0.0003	0.0003	0.0003	0.0003
0.0003	0.0003	0.0003	0.0003	0.0003	0.0003	0.0003	0.0003	0.0003	0.0003
0.0079	0.0003	0.0003	0.0003	0.0003	0.0003	0.0003	0.0003	0.0003	0.0003
0.0003	0.0003	0.0003	0.0003	0.0003	0.0003	0.0003	0.0003	0.0003	0.0003
0.0077	0.0003	0.0003	0.0003	0.0003	0.0003	0.0014	0.0003	0.0003	0.0003
0.0003	0.0003	0.0003	0.0003	0.0003	0.0003	0.0003	0.0003	0.0003	0.0003
0.0338	0.0077	0.0003	0.0003	0.0003	0.0003	0.0003	0.0003	0.0031	0.0013
0.0003	0.0026	0.0003	0.0003	0.0028	0.0032	0.0040	0.0003	0.0003	0.0003
0.0003	0.0024	0.0058	0.0003	0.0029	0.0023	0.0034	0.0016	0.0003	0.0019
0.0061	0.0003	0.0003	0.0003	0.0003	0.0003	0.0003	0.0003	0.0003	0.0003
0.0003	0.0003	0.0003	0.0003	0.0003	0.0003	0.0003	0.0003	0.0003	0.0003
0.1545	0.1493	0.2310	0.1927	0.1658	0.1589	0.1833	0.2088	0.1595	0.1767

CA 13 VEST IL-1β 1	CA 13 VEST IL-1β 2	CA 13 VEST IL-1β 3	CA 14 VEST IL-1β 1	CA 14 VEST IL-1β 2	CA 14 VEST IL-1β 3	CA 20 VEST IL-1β 1	CA 20 VEST IL-1β 2	CA 20 VEST IL-1β 3	CA 13 VULV IL-1β 1
0.0003	0.0003	0.0003	0.0023	0.0003	0.0025	0.0003	0.0003	0.0003	0.0003
0.0090	0.0003	0.0003	0.0003	0.0003	0.0003	0.0136	0.0003	0.0003	0.0003
0.0003	0.0003	0.0003	0.0003	0.0003	0.0003	0.0003	0.0003	0.0003	0.0003
0.0003	0.0003	0.0003	0.0003	0.0003	0.0003	0.0003	0.0003	0.0003	0.0003
0.0003	0.4231	0.4679	0.1174	0.1040	0.0003	0.9229	0.7852	0.6800	0.1597
0.0059	0.0003	0.0036	0.0003	0.0003	0.0003	0.0068	0.0003	0.0074	0.0003
0.0003	0.0003	0.0003	0.0003	0.0003	0.0003	0.0003	0.0003	0.0003	0.0003
0.0003	0.0003	0.0003	0.0003	0.0003	0.0003	0.0003	0.0003	0.0003	0.0003
0.0003	0.0003	0.0003	0.0003	0.0003	0.0003	0.0003	0.0003	0.0003	0.0003
33.3878	31.2915	45.5497	9.8210	8.5857	9.8066	73.7265	66.9768	62.8840	15.6239
0.0407	0.0191	0.0443	0.0003	0.0003	0.0003	0.0263	0.0283	0.0003	0.0131
0.0003	0.0003	0.0003	0.0003	0.0003	0.0003	0.0003	0.1081	0.0777	0.0003
0.0003	0.0003	0.0003	0.0003	0.0003	0.0003	0.0003	0.0003	0.0003	0.0003
0.0003	0.0003	0.0003	0.0003	0.0003	0.0003	0.0003	0.0003	0.0003	0.0003
0.0003	0.0003	0.0003	0.0003	0.0003	0.0003	0.0003	0.0003	0.0003	0.0003
0.0003	0.0350	0.0775	0.0003	0.0003	0.0182	0.0003	0.0432	0.0182	0.0441
1.3279	1.1821	1.4660	0.3739	0.3362	0.3804	1.7899	1.6499	1.6073	0.3752
33.6319	28.5080	42.3914	9.8645	8.8990	9.5137	73.8121	65.0811	59.0334	17.2875
0.0003	0.0003	0.0003	0.0003	0.0003	0.0003	0.0003	0.0003	0.0003	0.0003
0.0003	0.0003	0.0003	0.0003	0.0003	0.0003	0.0390	0.0003	0.0405	0.0003
0.0003	0.0003	0.0003	0.0003	0.0003	0.0003	0.0003	0.0003	0.0003	0.0003
0.0003	0.0003	0.0003	0.0314	0.0003	0.0003	0.0003	0.0003	0.0003	0.0003
0.0003	0.0003	0.0003	0.0003	0.0003	0.0003	0.0003	0.0003	0.0003	0.0003
0.0003	0.0003	0.0003	0.0003	0.0003	0.0003	0.0003	0.0003	0.0003	0.0003
0.0441	0.0003	0.0698	0.0003	0.0003	0.0003	0.0599	0.0493	0.0359	0.0003
0.0003	0.0003	0.0003	0.0003	0.0214	0.0231	0.0003	0.0003	0.0003	0.0286
0.0003	0.0003	0.0003	0.0003	0.0003	0.0003	0.0003	0.0003	0.1355	0.0003
0.0003	0.0003	0.0003	0.0003	0.0003	0.0003	0.0003	0.0003	0.0003	0.0003
0.0003	0.0003	0.0116	0.0003	0.0003	0.0003	0.0003	0.0003	0.0003	0.0003
0.0003	0.0003	0.0003	0.0003	0.0003	0.0003	0.0003	0.0460	0.0003	0.0215
0.0003	0.0057	0.0003	0.0003	0.0003	0.0003	0.0070	0.0003	0.0003	0.0073
0.0003	0.0003	0.0003	0.0003	0.0003	0.0003	0.0003	0.0003	0.0003	0.0003
0.0003	0.0003	0.0003	0.1051	0.0003	0.0296	0.0003	0.0537	0.0003	0.0003
0.0003	0.0003	0.0070	0.0113	0.0087	0.0028	0.0003	0.0003	0.0003	0.0003
0.0003	0.0003	0.0003	0.0003	0.0003	0.0003	0.0003	0.0003	0.0003	0.0003
0.0003	0.0003	0.0003	0.0003	0.0003	0.0003	0.0003	0.0003	0.0003	0.0003
0.0003	0.0003	0.0003	0.0003	0.0003	0.0045	0.0003	0.0003	0.0003	0.0003
0.0469	0.0644	0.0659	0.0497	0.0459	0.0003	0.0552	0.0483	0.0328	0.0546
0.0003	0.0003	0.0003	0.0003	0.0003	0.0003	0.0003	0.0003	0.0003	0.0003
0.0003	0.0003	0.0003	0.0003	0.0003	0.0003	0.0003	0.0003	0.0003	0.0003
0.0257	0.0003	0.0003	0.0041	0.0080	0.0003	0.0003	0.0003	0.0003	0.0003
0.0132	0.0360	0.0439	0.0042	0.0003	0.0111	0.0003	0.0003	0.0085	0.0288
0.0003	0.0003	0.0003	0.0003	0.0003	0.0003	0.0003	0.0003	0.0003	0.0003
0.0035	0.0068	0.0054	0.0044	0.0041	0.0053	0.0073	0.0047	0.0109	0.0040
0.0113	0.0027	0.0110	0.0063	0.0204	0.0139	0.0101	0.0003	0.0047	0.0140
0.0003	0.0003	0.0003	0.0003	0.0003	0.0003	0.0003	0.0003	0.0003	0.0003
0.0003	0.0003	0.0003	0.0003	0.0003	0.0003	0.0057	0.0003	0.0003	0.0003
0.0003	0.0003	0.0003	0.0003	0.0003	0.0003	0.0003	0.0003	0.0003	0.0003
0.0040	0.0003	0.0003	0.0003	0.0026	0.0052	0.0107	0.0003	0.0057	0.0003

0.0003	0.0003	0.0003	0.0003	0.0003	0.0003	0.0003	0.0003	0.0003	0.0003
0.0003	0.0003	0.0003	0.0003	0.0003	0.0003	0.0003	0.0003	0.0003	0.0003
0.0003	0.0003	0.0003	0.0003	0.0003	0.0003	0.0003	0.0003	0.0003	0.0003
0.9674	1.2398	6.4043	3.1968	1.6359	1.7711	1.1428	2.1819	1.9474	3.5599
0.0003	0.0003	0.0003	0.0003	0.0003	0.0003	0.0003	0.0003	0.0003	0.0003
0.0003	0.0003	0.0003	0.0003	0.0003	0.0003	0.0003	0.0003	0.0003	0.0003
0.0003	0.0003	0.0003	0.0003	0.0003	0.0003	0.0003	0.0003	0.0003	0.0003
0.0003	0.0003	0.0003	0.0003	0.0003	0.0003	0.0003	0.2953	0.1593	0.4633
0.0003	0.0003	0.0003	0.0003	0.0003	0.0003	0.0003	0.0003	0.0003	0.0003
0.0003	0.0003	0.0003	0.0003	0.0003	0.0003	0.0003	0.0003	0.0003	0.0003
0.0003	0.0003	0.0003	0.0003	0.0003	0.0003	0.0003	0.0003	0.0003	0.0003
0.0003	0.0003	0.0003	0.0003	0.0003	0.0003	0.0003	0.0003	0.0003	0.0003
0.1225	0.1198	0.1324	0.0968	0.0745	0.0618	0.0909	0.0992	0.1003	0.0728
0.0003	0.0003	0.0003	0.0003	0.0003	0.0003	0.0003	0.0003	0.0003	0.0003
0.2600	0.1737	0.1720	0.0446	0.0488	0.1155	0.2497	0.1206	0.2568	0.0555
0.0003	0.0003	0.0003	0.0003	0.0003	0.0003	0.0003	0.0003	0.0003	0.0003
0.1352	0.1436	0.2478	0.1512	0.0868	0.1511	0.1161	0.1189	0.1465	0.1453
0.0003	0.0003	0.0003	0.0003	0.0003	0.0003	0.0003	0.0003	0.0003	0.0003
0.0003	0.0003	0.0003	0.0003	0.0003	0.0003	0.0003	0.0003	0.0003	0.0003
0.0003	0.0003	0.0003	0.0003	0.0462	0.0330	0.0586	0.0611	0.0293	0.0003
0.0003	0.0003	0.0003	0.0003	0.0003	0.0003	0.0003	0.0003	0.0003	0.0003
0.0003	0.0003	0.0003	0.0003	0.0003	0.0003	0.0003	0.0003	0.0003	0.0003
0.0003	0.0003	0.0003	0.0003	0.0003	0.0003	0.0003	0.0003	0.0003	0.0003
0.0003	0.0003	0.0003	0.0003	0.0003	0.0003	0.0003	0.0003	0.0003	0.0003
0.0003	0.0003	0.0003	0.0003	0.0003	0.0003	0.0003	0.0003	0.0003	0.0003
0.0003	0.0003	0.0003	0.0003	0.0003	0.0003	0.0003	0.0003	0.0003	0.0003
0.0003	0.0003	0.0003	0.0003	0.0003	0.0003	0.0003	0.0003	0.0003	0.0003
0.0003	0.0003	0.0003	0.0003	0.0003	0.0003	0.0003	0.0003	0.0003	0.0003
0.0003	0.0003	0.0003	0.0003	0.0003	0.0003	0.0003	0.0003	0.0003	0.0003
0.0029	0.0003	0.0003	0.0003	0.0003	0.0003	0.0003	0.0003	0.0268	0.0003
0.0003	0.0294	0.0003	0.0298	0.0611	0.0140	0.0825	0.0394	0.0453	0.0135
0.0166	0.0003	0.0003	0.0003	0.0122	0.0003	0.0106	0.0003	0.0003	0.0327
0.0003	0.0003	0.0003	0.0003	0.0003	0.0003	0.0003	0.0003	0.0003	0.0003
0.0003	0.0003	0.0003	0.0003	0.0003	0.0003	0.0003	0.0003	0.0003	0.0003
0.0078	0.0003	0.0080	0.0003	0.0064	0.0003	0.0003	0.0003	0.0003	0.0003
0.1093	0.2221	0.2523	0.1354	0.1557	0.1360	0.1431	0.1084	0.1608	0.2717
0.0003	0.0003	0.0003	0.0003	0.0003	0.0003	0.0003	0.0003	0.0003	0.0003
0.2242	0.2985	0.3598	0.0989	0.2030	0.2174	0.2859	0.1900	0.2759	0.3188
0.0003	0.0003	0.0003	0.0003	0.0003	0.0703	0.0003	0.0003	0.0003	0.2247
0.5114	0.5913	4.3963	2.1925	0.7295	0.0003	0.5807	0.0003	1.0287	2.3813
0.0041	0.0003	0.0030	0.0003	0.0011	0.0019	0.0003	0.0003	0.0003	0.0003
0.0417	0.0319	0.0428	0.0003	0.0122	0.0225	0.0168	0.0277	0.0240	0.0347
5.0238	4.6379	6.6306	3.3696	2.8234	3.3452	5.7427	5.9177	5.2332	3.3133
1.2263	1.0954	1.5009	0.5518	0.4714	0.5700	1.2363	1.4422	1.1643	0.7341
0.0003	0.0003	0.0003	0.0003	0.0003	0.0003	0.0003	0.0003	0.0003	0.0003
0.3092	0.2954	0.3497	0.2388	0.2214	0.2544	0.3673	0.3877	0.4197	0.1780
0.0607	0.0629	0.0649	0.0461	0.0477	0.0619	0.1132	0.1050	0.0790	0.0315
0.0003	0.0003	0.0003	0.0003	0.0003	0.0003	0.0003	0.0003	0.0003	0.0003
0.0003	0.0003	0.0003	0.0003	0.0003	0.0003	0.0003	0.0003	0.0003	0.0003
0.0003	0.0003	0.0109	0.0003	0.0003	0.0003	0.0070	0.0003	0.0146	0.0003
0.0003	0.0003	0.0003	0.0003	0.0003	0.0003	0.0003	0.0003	0.0003	0.0003
0.0003	0.0003	0.0003	0.0003	0.0003	0.0003	0.0003	0.0003	0.0003	0.0003
0.0569	0.0516	0.0369	0.0385	0.0257	0.0588	0.0474	0.0474	0.0396	0.0363

0.0003	0.0003	0.1198	0.0003	0.0003	0.0003	0.0003	0.0003	0.0003	0.0449
0.0003	0.0003	0.0567	0.0003	0.0003	0.0003	0.0003	0.0003	0.0003	0.0177
0.0003	0.0003	0.0003	0.0003	0.0003	0.0003	0.0003	0.0003	0.0003	0.0003
0.0003	0.0003	0.0003	0.0003	0.0003	0.0003	0.0003	0.0003	0.0103	0.0003
0.0003	0.0003	0.0003	0.0194	0.0110	0.0167	0.0003	0.0117	0.0003	0.0003
0.0132	0.0129	0.0156	0.0178	0.0135	0.0183	0.0240	0.0253	0.0242	0.0120
0.0003	0.0003	0.0003	0.0003	0.0003	0.0003	0.0003	0.0003	0.0003	0.0003
0.0003	0.0003	0.0003	0.0003	0.0003	0.0003	0.0003	0.0003	0.0003	0.0003
0.0535	1.4196	0.0003	0.0003	0.0003	0.0003	0.0581	0.0003	0.0326	0.2060
0.0003	0.0003	0.0003	0.0003	0.0003	0.0003	0.0003	0.0003	0.0003	0.0003
0.0003	0.0003	0.0003	0.0003	0.0003	0.0003	0.0003	0.0003	0.0003	0.0003
0.0133	0.0003	0.0265	0.0084	0.0003	0.0092	0.0253	0.0200	0.0218	0.0129
0.0003	0.0003	0.0003	0.0003	0.0003	0.0003	0.0003	0.0003	0.0003	0.0003
0.0003	0.0003	0.0003	0.0003	0.0003	0.0003	0.0003	0.0466	0.0003	0.0003
0.0003	0.0003	0.0003	0.0003	0.0003	0.0003	0.0003	0.0003	0.0003	0.0003
0.0003	0.0003	0.0003	0.0003	0.0003	0.0003	0.0003	0.0003	0.0360	0.0361
0.0003	0.0003	0.0003	0.0003	0.0003	0.0003	0.0003	0.0003	0.0003	0.0003
0.0003	0.0003	0.0003	0.0003	0.0003	0.0003	0.0003	0.0003	0.0003	0.0003
0.0003	0.0003	0.0003	0.0003	0.0003	0.0003	0.0003	0.0003	0.0003	0.0003
0.3219	0.3036	0.4746	0.0813	0.0931	0.1079	0.8718	0.8292	0.6509	0.1714
0.0003	0.0003	0.0003	0.0003	0.0003	0.0003	0.0003	0.0003	0.0003	0.0003
0.0003	0.0003	0.0003	0.0003	0.0416	0.0198	0.0263	0.0163	0.0486	0.0003
0.0003	0.0003	0.0003	0.0003	0.0288	0.0594	0.0003	0.0240	0.0003	0.0477
0.0003	0.0003	0.0003	0.0003	0.0003	0.0003	0.0003	0.0003	0.0003	0.0003
0.0003	0.0003	0.0003	0.0003	0.0003	0.0003	0.0003	0.0003	0.0003	0.0003
0.0003	0.0003	0.0003	0.0003	0.0003	0.0003	0.0003	0.0003	0.0003	0.0003
0.0003	0.0003	0.0003	0.0003	0.0003	0.0003	0.0003	0.0003	0.0003	0.0003
0.0003	0.0003	0.0003	0.0003	0.0003	0.0003	0.0003	0.0003	0.0003	0.0003
0.0100	0.0143	0.0003	0.0081	0.0003	0.0003	0.0003	0.0003	0.0264	0.0104
0.0003	0.0003	0.0003	0.0003	0.0003	0.0003	0.0003	0.0003	0.0003	0.0003
0.0003	0.0003	0.0003	0.0003	0.0003	0.0003	0.0003	0.0003	0.0003	0.0003
0.0003	0.0032	0.0174	0.0003	0.0003	0.0014	0.0199	0.0061	0.0003	0.0003
0.0003	0.0003	0.0003	0.0003	0.0003	0.0003	0.0003	0.0003	0.0003	0.0176
0.0003	0.0003	0.0003	0.0003	0.0003	0.0003	0.0093	0.0003	0.0003	0.0003
0.0003	0.0003	0.0003	0.0003	0.0003	0.0003	0.0003	0.0003	0.0003	0.0003
0.0003	0.0003	0.0003	0.0003	0.0003	0.0003	0.0003	0.0003	0.0003	0.0003
0.0003	0.0003	0.0003	0.0003	0.0003	0.0003	0.0003	0.0003	0.0003	0.0003
0.0003	0.0003	0.0003	0.0003	0.0003	0.0003	0.0003	0.0003	0.0003	0.0003
0.0003	0.0003	0.0003	0.0003	0.0003	0.0003	0.0003	0.0003	0.0003	0.0003
0.0003	0.0210	0.0311	0.0250	0.0003	0.0003	0.0032	0.0003	0.0003	0.0179
0.0003	0.0003	0.0046	0.0055	0.0077	0.0066	0.0003	0.0003	0.0035	0.0041
0.0003	0.0003	0.0003	0.0025	0.0028	0.0003	0.0012	0.0003	0.0013	0.0003
0.0003	0.0003	0.0003	0.0003	0.0003	0.0003	0.0003	0.0003	0.0003	0.0003
0.0003	0.0003	0.0003	0.0003	0.0003	0.0003	0.0003	0.0003	0.0003	0.0003
0.2762	0.2002	0.2568	0.2088	0.1836	0.1614	0.1613	0.1948	0.1360	0.2206

CA 13 VULV IL-1β 2	CA 13 VULV IL-1β 3	CA 14 VULV IL-1β 1	CA 14 VULV IL-1β 2	CA 14 VULV IL-1β 3	CA 20 VULV IL-1β 1	CA 20 VULV IL-1β 2	CA 20 VULV IL-1β 3	CO 10 VEST TPPU + IL-1β 1 **13**
0.0003	0.0003	0.0003	0.0003	0.0003	0.0003	0.0003	0.0003	0.0003
0.0003	0.0100	0.0003	0.0003	0.0003	0.0003	0.0003	0.0003	0.0003
0.0003	0.0003	0.0003	0.0003	0.0003	0.0003	0.0003	0.0003	0.0003
0.0003	0.0003	0.0003	0.0003	0.0003	0.0003	0.0003	0.0003	0.0003
0.2074	0.1912	0.4480	0.4197	0.4283	0.2373	0.3778	0.3086	0.0992
0.0003	0.0003	0.0003	0.0003	0.0003	0.0003	0.0044	0.0003	0.0074
0.0003	0.0003	0.0003	0.0003	0.0003	0.0003	0.0003	0.0003	0.0003
0.0003	0.0003	0.0003	0.0003	0.0003	0.0003	0.0003	0.0003	0.0003
0.0003	0.0003	0.0003	0.0003	0.0003	0.0003	0.0003	0.0003	0.0003
19.2009	18.0995	42.1325	37.2994	41.3921	50.7996	34.2916	32.6023	21.5480
0.0003	0.0175	0.0223	0.0244	0.0189	0.0400	0.0339	0.0003	0.0003
0.0003	0.0303	0.0509	0.0491	0.0769	0.0003	0.0003	0.0526	0.0003
0.0003	0.0003	0.0767	0.0003	0.0003	0.0003	0.0003	0.0003	0.0003
0.0003	0.0003	0.0003	0.0003	0.0003	0.0003	0.0003	0.0003	0.0003
0.0003	0.0003	0.0003	0.0003	0.0003	0.0003	0.0003	0.0003	0.0003
0.0936	0.0003	0.0003	0.0003	0.0350	0.0003	0.0173	0.0003	0.0003
0.3902	0.3617	1.1880	1.1034	1.3214	1.3383	0.9991	0.8200	0.6246
17.9544	18.7940	41.2182	37.6754	40.6218	49.4464	33.9235	28.8007	20.5069
0.0003	0.0003	0.0003	0.0003	0.0003	0.0003	0.0003	0.0003	0.0003
0.0003	0.0003	0.0003	0.0003	0.0003	0.0003	0.0254	0.0189	0.0003
0.0003	0.0003	0.0003	0.0003	0.0003	0.0003	0.0003	0.0003	0.0003
0.0003	0.0003	0.0003	0.0324	0.0003	0.0003	0.0003	0.0003	0.0003
0.0003	0.0003	0.0003	0.0003	0.0003	0.0003	0.0003	0.0003	0.0003
0.0003	0.0003	0.0003	0.0003	0.0003	0.0003	0.0003	0.0003	0.0003
0.0003	0.0003	0.0003	0.0003	0.0003	0.0292	0.0177	0.0232	0.0003
0.0003	0.0392	0.0383	0.0722	0.0003	0.0003	0.0003	0.0003	0.0003
0.0247	0.0003	0.0003	0.0594	0.0003	0.0003	0.0003	0.0003	0.0003
0.0003	0.0003	0.0003	0.0003	0.0003	0.0003	0.0003	0.0003	0.0003
0.0173	0.0003	0.0003	0.0003	0.0003	0.0003	0.0003	0.0003	0.0003
0.0003	0.0003	0.0003	0.0003	0.0166	0.0003	0.0003	0.0003	0.0003
0.0003	0.0003	0.0047	0.0003	0.0003	0.0003	0.0003	0.0003	0.0003
0.0003	0.0003	0.0003	0.0003	0.0003	0.0104	0.0003	0.0003	0.0003
0.0003	0.0619	0.0689	0.0003	0.0003	0.4628	0.0003	0.0003	0.0003
0.0003	0.0074	0.0003	0.0053	0.0047	0.0003	0.0075	0.0003	0.0003
0.0003	0.0003	0.0003	0.0003	0.0003	0.0003	0.0003	0.0003	0.0003
0.0132	0.0287	0.0003	0.0003	0.0126	0.0003	0.0098	0.0003	0.0003
0.0003	0.0003	0.0003	0.0003	0.0003	0.0003	0.0070	0.0003	0.0003
0.0555	0.0716	0.0516	0.0558	0.0350	0.0566	0.0352	0.0003	0.0418
0.0003	0.0003	0.0003	0.0003	0.0003	0.0003	0.0003	0.0003	0.0003
0.0003	0.0003	0.0003	0.0003	0.0003	0.0003	0.0003	0.0003	0.0003
0.0042	0.0003	0.0003	0.0003	0.0003	0.0003	0.0003	0.0003	0.0003
0.0385	0.0136	0.0127	0.0003	0.0003	0.0059	0.0082	0.0075	0.0169
0.0003	0.0003	0.0003	0.0003	0.0003	0.0003	0.0003	0.0003	0.0003
0.0041	0.0024	0.0033	0.0038	0.0157	0.0077	0.0156	0.0074	0.0058
0.0183	0.0230	0.0116	0.0043	0.0087	0.0097	0.0113	0.0081	0.0173
0.0003	0.0003	0.0003	0.0003	0.0003	0.0003	0.0003	0.0003	0.0003
0.0003	0.0003	0.0003	0.0003	0.0003	0.0003	0.0003	0.0003	0.0003
0.0003	0.0003	0.0003	0.0003	0.0003	0.0003	0.0003	0.0003	0.0003
0.0003	0.0038	0.0003	0.0003	0.0003	0.0003	0.0077	0.0003	0.0080

0.0003	0.0003	0.0003	0.0003	0.0003	0.0003	0.0003	0.0003	0.0003
0.0003	0.0003	0.0003	0.0003	0.0003	0.0003	0.0003	0.0356	0.0003
0.0003	0.0003	0.0003	0.0003	0.0040	0.0003	0.0003	0.0003	0.0003
4.7680	1.6108	1.4004	1.7051	2.3327	2.8275	2.3511	1.3230	1.0121
0.0003	0.0003	0.0003	0.0003	0.0003	0.0003	0.0003	0.0003	0.0003
0.0003	0.0003	0.0003	0.0003	0.0003	0.0003	0.0003	0.0003	0.0003
0.0003	0.0003	0.0003	0.0003	0.0003	0.0003	0.0003	0.0003	0.0003
0.0003	0.0003	0.0003	0.0003	0.0003	0.0003	0.0003	0.0003	0.0003
0.0003	0.0003	0.0003	0.0003	0.0003	0.0003	0.0003	0.0003	0.0003
0.0003	0.0003	0.0003	0.0003	0.0003	0.0003	0.0003	0.0003	0.0003
0.0003	0.0003	0.0003	0.0003	0.0003	0.0003	0.0003	0.0003	0.0003
0.0003	0.0003	0.0003	0.0003	0.0003	0.0003	0.0003	0.0003	0.0003
0.1211	0.0784	0.1149	0.1376	0.1495	0.0962	0.0921	0.1154	0.0515
0.0003	0.0003	0.0003	0.0003	0.0003	0.0003	0.0003	0.0003	0.0003
0.1024	0.1125	0.1571	0.2962	0.1573	0.1374	0.1938	0.1122	0.0671
0.0003	0.0003	0.0003	0.0003	0.0003	0.0003	0.0003	0.0003	0.0003
0.1085	0.0960	0.1834	0.1285	0.1900	0.1342	0.0817	0.1154	0.0939
0.0003	0.0003	0.0003	0.0003	0.0003	0.0003	0.0003	0.0003	0.0003
0.0003	0.0003	0.0003	0.0003	0.0003	0.0003	0.0003	0.0003	0.0003
0.0003	0.0003	0.0406	0.0393	0.0390	0.0345	0.0378	0.0396	0.0519
0.0003	0.0003	0.0003	0.0003	0.0003	0.0003	0.0003	0.0003	0.0003
0.0003	0.0003	0.0003	0.0003	0.0003	0.0003	0.0003	0.0003	0.0003
0.0003	0.0003	0.0003	0.0003	0.0003	0.0003	0.0003	0.0338	0.0003
0.0003	0.0003	0.0003	0.0322	0.0003	0.0003	0.0003	0.0003	0.0003
0.0127	0.0003	0.0003	0.0003	0.0003	0.0003	0.0003	0.0003	0.0003
0.0003	0.0003	0.0003	0.0003	0.0003	0.0003	0.0003	0.0003	0.0003
0.0003	0.0003	0.0003	0.0003	0.0003	0.0003	0.0003	0.0003	0.0003
0.0003	0.0003	0.0003	0.0003	0.0003	0.0003	0.0003	0.0003	0.0003
0.0003	0.0003	0.0003	0.0003	0.0003	0.0003	0.0003	0.0003	0.0003
0.0003	0.0087	0.0003	0.0003	0.0003	0.0189	0.0003	0.0003	0.0003
0.0003	0.0148	0.0003	0.0360	0.0431	0.0544	0.0003	0.0222	0.0003
0.0220	0.0003	0.0003	0.0146	0.0121	0.0003	0.0003	0.0003	0.0003
0.0003	0.0003	0.0003	0.0003	0.0003	0.0003	0.0003	0.0003	0.0003
0.0003	0.0003	0.0003	0.0003	0.0003	0.0003	0.0003	0.0003	0.0003
0.0003	0.0003	0.0043	0.0003	0.0003	0.0003	0.0022	0.0003	0.0096
0.3607	0.2061	0.3028	0.1869	0.1449	0.1452	0.1402	0.2067	0.1319
0.0003	0.0003	0.0003	0.0003	0.0003	0.0003	0.0003	0.0003	0.0003
0.4591	0.3530	0.2701	0.0003	0.2298	0.1647	0.2439	0.3494	0.3176
0.3777	0.0003	0.0003	0.0003	0.0003	0.0003	0.0620	0.0515	0.0003
3.4057	0.0003	0.6300	0.8291	2.7012	0.0003	1.0805	0.0003	0.0003
0.0003	0.0003	0.0003	0.0003	0.0003	0.0003	0.0003	0.0003	0.0019
0.0234	0.0184	0.0308	0.0219	0.0314	0.0140	0.0189	0.0305	0.0145
3.9957	2.4965	8.6024	7.4777	9.6934	6.3974	5.0142	3.5888	4.5319
0.9649	0.5663	1.6469	1.5443	1.8557	1.2644	1.0434	0.8971	0.9307
0.0003	0.0003	0.0003	0.0003	0.0003	0.0003	0.0003	0.0003	0.0003
0.2153	0.1512	0.4244	0.3407	0.4555	0.5197	0.4134	0.2637	0.2896
0.0370	0.0182	0.0772	0.0935	0.0693	0.0711	0.0745	0.0588	0.0515
0.0003	0.0003	0.0003	0.0003	0.0003	0.0003	0.0003	0.0003	0.0003
0.0003	0.0003	0.0003	0.0003	0.0003	0.0003	0.0003	0.0003	0.0003
0.0003	0.0043	0.0003	0.0003	0.0003	0.0003	0.0108	0.0064	0.0103
0.0003	0.0003	0.0003	0.0003	0.0003	0.0003	0.0003	0.0003	0.0003
0.0003	0.0003	0.0003	0.0003	0.0003	0.0003	0.0003	0.0003	0.0003
0.0227	0.0343	0.0663	0.0487	0.0510	0.0408	0.0347	0.0604	0.0342

0.0734	0.0003	0.0003	0.0003	0.0003	0.0003	0.0003	0.0003	0.0003
0.0444	0.0003	0.0003	0.0003	0.0003	0.0003	0.0003	0.0003	0.0003
0.0003	0.0003	0.0003	0.0003	0.0003	0.0003	0.0003	0.0003	0.0003
0.0003	0.0003	0.0003	0.0003	0.0003	0.0003	0.0003	0.0003	0.0003
0.0003	0.0003	0.0116	0.0130	0.0196	0.0003	0.0003	0.0140	0.0003
0.0132	0.0136	0.0301	0.0317	0.0302	0.0390	0.0252	0.0326	0.0152
0.0003	0.0003	0.0003	0.0003	0.0003	0.0003	0.0003	0.0003	0.0003
0.0003	0.0003	0.0003	0.0003	0.0003	0.0003	0.0003	0.0058	0.0003
0.0003	0.4099	0.0431	0.0003	0.0003	0.0003	0.0003	0.0003	0.0003
0.0003	0.0003	0.0003	0.0003	0.0003	0.0003	0.0003	0.0003	0.0202
0.0003	0.0003	0.0003	0.0003	0.0003	0.0003	0.0003	0.0003	0.0003
0.0003	0.0003	0.0147	0.0086	0.0122	0.0117	0.0159	0.0003	0.0126
0.0003	0.0003	0.0003	0.0003	0.0003	0.0003	0.0003	0.0003	0.0003
0.0003	0.0003	0.0003	0.0003	0.0003	0.0003	0.0003	0.0003	0.0003
0.0003	0.0003	0.0003	0.0003	0.0003	0.0003	0.0003	0.0003	0.0003
0.0003	0.0003	0.0003	0.0003	0.0003	0.0003	0.0003	0.0003	0.0003
0.0003	0.0003	0.0003	0.0003	0.0142	0.0003	0.0003	0.0003	0.0003
0.0003	0.0003	0.0210	0.0003	0.0003	0.0003	0.0003	0.0003	0.0003
0.0003	0.0003	0.0003	0.0003	0.0003	0.0003	0.0003	0.0003	0.0003
0.2578	0.2294	0.4914	0.4450	0.4389	0.6288	0.4663	0.4732	0.2307
0.0003	0.0003	0.0003	0.0003	0.0003	0.0003	0.0003	0.0003	0.0003
0.0102	0.0003	0.0003	0.0253	0.0003	0.0003	0.0003	0.0201	0.0127
0.0003	0.0344	0.0003	0.0003	0.0003	0.0003	0.0003	0.0314	0.0003
0.0003	0.0003	0.0003	0.0003	0.0003	0.0003	0.0003	0.0003	0.0003
0.0003	0.0003	0.0003	0.0003	0.0003	0.0003	0.0003	0.0003	0.0003
0.0003	0.0003	0.0003	0.0003	0.0003	0.0003	0.0003	0.0003	0.0003
0.0003	0.0003	0.0003	0.0003	0.0003	0.0003	0.0003	0.0453	0.0003
0.0003	0.0003	0.0003	0.0003	0.0003	0.0003	0.0003	0.0003	0.0475
0.0003	0.0003	0.0003	0.0077	0.0003	0.0003	0.0003	0.0137	0.0003
0.0003	0.0003	0.0003	0.0003	0.0003	0.0003	0.0003	0.0003	0.0003
0.0003	0.0003	0.0003	0.0003	0.0003	0.0003	0.0003	0.0003	0.0003
0.0003	0.0196	0.0003	0.0003	0.0092	0.0003	0.0127	0.0003	0.0261
0.0175	0.0003	0.0235	0.0003	0.0109	0.0003	0.0003	0.0003	0.0074
0.0003	0.0003	0.0003	0.0003	0.0101	0.0003	0.0003	0.0003	0.0003
0.0003	0.0003	0.0003	0.0003	0.0003	0.0003	0.0003	0.0003	0.0003
0.0003	0.0003	0.0003	0.0003	0.0003	0.0003	0.0003	0.0003	0.0003
0.0003	0.0003	0.0003	0.0003	0.0003	0.0003	0.0003	0.0003	0.0003
0.0003	0.0003	0.0003	0.0003	0.0003	0.0003	0.0003	0.0014	0.0003
0.0003	0.0003	0.0003	0.0003	0.0003	0.0003	0.0003	0.0003	0.0003
0.0003	0.0398	0.0249	0.0301	0.0029	0.0003	0.0003	0.0003	0.0118
0.0003	0.0003	0.0003	0.0070	0.0039	0.0028	0.0032	0.0040	0.0003
0.0003	0.0003	0.0003	0.0003	0.0003	0.0029	0.0023	0.0034	0.0003
0.0003	0.0003	0.0003	0.0003	0.0003	0.0003	0.0003	0.0003	0.0003
0.0003	0.0003	0.0003	0.0003	0.0003	0.0003	0.0003	0.0003	0.0003
0.2577	0.2367	0.2280	0.2120	0.1608	0.1658	0.1589	0.1833	0.2126

CO 10 VEST TPPU + IL-1β 2 **14**	CO 10 VEST TPPU + IL-1β 3 **15**	CO 21 VEST TPPU + IL-1β 1 **157**	CO 21 VEST TPPU + IL-1β 2 **158**	CO 21 VEST TPPU + IL-1β 3 **159**	CO 23 VEST TPPU + IL-1β 1 **193**
0.0003	0.0003	0.0022	0.0003	0.0029	0.0003
0.0003	0.0003	0.0003	0.0003	0.0003	0.0003
0.0003	0.0003	0.0003	0.0003	0.0003	0.0003
0.0003	0.0003	0.0003	0.0003	0.0003	0.0003
0.1600	0.1810	0.5116	0.5936	0.4217	2.1626
0.0003	0.0003	0.0003	0.0003	0.0003	0.0095
0.0003	0.0003	0.0003	0.0003	0.0003	0.0107
0.0003	0.0003	0.0003	0.0003	0.0003	0.0033
0.0003	0.0003	0.0003	0.0003	0.0003	0.0003
18.7212	18.8258	46.3112	53.2163	39.4606	143.6492
0.0003	0.0003	0.0247	0.0313	0.0178	0.1059
0.0003	0.0003	0.0003	0.0003	0.0456	0.3368
0.0003	0.0003	0.0003	0.0003	0.0003	0.0003
0.0003	0.0003	0.0003	0.0003	0.0003	0.0003
0.0003	0.0003	0.0003	0.0003	0.0003	0.0003
0.0003	0.0003	0.0003	0.0003	0.0003	0.0003
0.5937	0.5937	1.7484	1.7134	1.2255	4.7506
17.6974	18.2265	47.8467	52.8739	40.2099	135.0037
0.0003	0.0003	0.0003	0.0003	0.0003	0.0003
0.0003	0.0003	0.0003	0.0003	0.0003	0.1278
0.0003	0.0003	0.0003	0.0003	0.0003	0.0003
0.0003	0.0003	0.0003	0.0003	0.0003	0.0003
0.0003	0.0003	0.0003	0.0003	0.0003	0.0003
0.0003	0.0003	0.0003	0.0003	0.0003	0.0003
0.0003	0.0003	0.0179	0.0280	0.0135	0.1336
0.0003	0.0134	0.0003	0.0696	0.0003	0.0003
0.0003	0.0003	0.0003	0.0003	0.0653	0.0003
0.0003	0.0003	0.0003	0.0003	0.0003	0.0003
0.0003	0.0003	0.0003	0.0003	0.0003	0.0003
0.0003	0.0003	0.0003	0.0130	0.0003	0.0242
0.0003	0.0003	0.0072	0.0003	0.0039	0.0003
0.0003	0.0003	0.0003	0.0003	0.0003	0.0408
0.0003	0.0347	0.0003	0.0816	0.0418	0.0003
0.0003	0.0003	0.0022	0.0003	0.0003	0.0003
0.0003	0.0003	0.0003	0.0003	0.0003	0.0003
0.0003	0.0003	0.0003	0.0003	0.0003	0.0003
0.0097	0.0185	0.0003	0.0003	0.0003	0.0003
0.0352	0.0645	0.0482	0.0430	0.0641	0.0673
0.0003	0.0003	0.0003	0.0003	0.0003	0.0003
0.0003	0.0003	0.0003	0.0003	0.0003	0.0003
0.0003	0.0003	0.0003	0.0003	0.0003	0.0036
0.0074	0.0078	0.0003	0.0003	0.0061	0.0003
0.0003	0.0003	0.0003	0.0003	0.0003	0.0003
0.0073	0.0023	0.0056	0.0069	0.0063	0.0025
0.0176	0.0110	0.0124	0.0121	0.0094	0.0003
0.0003	0.0003	0.0003	0.0003	0.0003	0.0003
0.0003	0.0003	0.0003	0.0003	0.0003	0.0003
0.0003	0.0003	0.0003	0.0003	0.0003	0.0003
0.0073	0.0048	0.0003	0.0096	0.0003	0.0117

0.0003	0.0003	0.0003	0.0003	0.0003	0.0003
0.0003	0.0003	0.0003	0.0003	0.0003	0.0055
0.0003	0.0003	0.0003	0.0003	0.0003	0.0003
0.0003	0.0003	1.6889	1.6853	1.4566	1.4263
0.0003	0.0003	0.0003	0.0003	0.0003	0.0003
0.0003	0.0003	0.0003	0.0003	0.0003	0.0003
0.0003	0.0003	0.0003	0.0003	0.0003	0.0003
0.0003	0.0003	0.0003	0.4113	0.4694	0.0003
0.0003	0.1083	0.0003	0.0003	0.0003	0.0189
0.0003	0.0003	0.0003	0.0003	0.0003	0.0003
0.0003	0.0003	0.0003	0.0003	0.0003	0.0003
0.0003	0.0003	0.0003	0.0003	0.0003	0.0003
0.0774	0.0745	0.2030	0.2457	0.1883	0.2350
0.0003	0.0003	0.0241	0.0003	0.0237	0.0003
0.0910	0.2258	0.1394	0.1306	0.1524	0.4919
0.0003	0.0003	0.0003	0.0003	0.0003	0.0003
0.0982	0.0820	0.1498	0.2115	0.1796	0.2035
0.0003	0.0003	0.0003	0.0003	0.0003	0.0003
0.0003	0.0003	0.0003	0.0003	0.0003	0.0003
0.0525	0.0003	0.0935	0.0782	0.0824	0.0821
0.0003	0.0003	0.0003	0.0003	0.0003	0.0003
0.0003	0.0282	0.0003	0.0003	0.0003	0.0164
0.0003	0.0003	0.0003	0.0003	0.0003	0.0003
0.0003	0.0003	0.0003	0.0003	0.0003	0.0003
0.0003	0.0003	0.0003	0.0003	0.0003	0.0003
0.0003	0.0003	0.0003	0.0003	0.0003	0.0003
0.0003	0.0003	0.0003	0.0003	0.0003	0.0003
0.0003	0.0003	0.0003	0.0003	0.0003	0.0003
0.0003	0.0003	0.0003	0.0003	0.0003	0.0003
0.0003	0.0003	0.0003	0.0003	0.0293	0.0003
0.0003	0.0901	0.1242	0.0003	0.1059	0.1565
0.0003	0.0231	0.0071	0.0003	0.0003	0.0003
0.0003	0.0003	0.0003	0.0003	0.0003	0.0565
0.0003	0.0003	0.0003	0.0003	0.0003	0.0003
0.0003	0.0003	0.0003	0.0003	0.0003	0.0003
0.1268	0.1603	0.1376	0.1641	0.1263	0.1470
0.0003	0.0003	0.0003	0.0003	0.0003	0.0003
0.3196	0.2939	0.2414	0.2863	0.2317	0.1486
0.0003	0.0003	0.0003	0.0003	0.0003	0.0003
0.0003	0.0003	0.0003	0.5345	0.3439	0.4145
0.0003	0.0029	0.0003	0.0003	0.0003	0.0003
0.0003	0.0176	0.0333	0.0336	0.0502	0.0363
4.1381	4.5150	8.4782	6.8795	7.1940	19.5618
0.8332	0.9801	1.7249	1.6200	1.6350	4.6613
0.0003	0.0003	0.0003	0.0003	0.0003	0.0003
0.2409	0.2733	0.6467	0.6287	0.5497	1.1605
0.0854	0.0003	0.1018	0.1164	0.1058	0.1952
0.0003	0.0003	0.0003	0.0003	0.0003	0.0003
0.0003	0.0003	0.0003	0.0003	0.0003	0.0113
0.0003	0.0003	0.0003	0.0198	0.0003	0.0283
0.0003	0.0003	0.0003	0.0003	0.0003	0.0003
0.0003	0.0003	0.0003	0.0003	0.0003	0.0003
0.0281	0.0620	0.0983	0.1043	0.0821	0.1264

0.0003	0.0003	0.0003	0.0003	0.0003	0.0003
0.0003	0.0003	0.0003	0.0003	0.0003	0.0003
0.0003	0.0003	0.0003	0.0003	0.0003	0.0003
0.0003	0.0003	0.0003	0.0003	0.0003	0.0003
0.0003	0.0130	0.0219	0.0003	0.0167	0.0284
0.0125	0.0132	0.0326	0.0281	0.0276	0.0235
0.0003	0.0003	0.0003	0.0003	0.0003	0.0003
0.0060	0.0003	0.0003	0.0003	0.0003	0.0003
0.0003	0.0003	0.0003	0.0003	0.0229	0.0412
0.0003	0.0003	0.0003	0.0003	0.0003	0.0003
0.0003	0.0003	0.0003	0.0003	0.0003	0.0003
0.0003	0.0126	0.0220	0.0003	0.0151	0.0450
0.0003	0.0003	0.0003	0.0003	0.0003	0.0003
0.0003	0.0003	0.0003	0.0003	0.0003	0.0003
0.0003	0.0003	0.0003	0.0003	0.0003	0.0649
0.0003	0.0302	0.0003	0.0003	0.0003	0.0003
0.0003	0.0003	0.0003	0.0003	0.0003	0.0287
0.0003	0.0003	0.0003	0.0003	0.0432	0.0003
0.0003	0.0003	0.0003	0.0003	0.0003	0.0003
0.1942	0.1807	0.6005	0.6184	0.5061	2.4745
0.0003	0.0003	0.0003	0.0003	0.0003	0.0003
0.0210	0.0003	0.0398	0.0003	0.0003	0.0623
0.0003	0.0003	0.0003	0.0506	0.0003	0.0919
0.0003	0.0003	0.0003	0.0003	0.0003	0.0003
0.0003	0.0003	0.0003	0.0003	0.0003	0.0003
0.0003	0.0003	0.0003	0.0003	0.0003	0.0003
0.0003	0.0003	0.0003	0.0003	0.0003	0.0003
0.0003	0.0003	0.0003	0.0003	0.0888	0.0003
0.0102	0.0097	0.0257	0.0048	0.0003	0.0003
0.0003	0.0003	0.0003	0.0003	0.0003	0.0003
0.0003	0.0003	0.0003	0.0003	0.0003	0.0003
0.0107	0.0195	0.0089	0.0118	0.0063	0.0085
0.0003	0.0003	0.0003	0.0003	0.0003	0.0003
0.0003	0.0003	0.0003	0.0003	0.0003	0.0079
0.0003	0.0003	0.0003	0.0003	0.0003	0.0003
0.0003	0.0003	0.0003	0.0003	0.0003	0.0123
0.0003	0.0003	0.0003	0.0003	0.0003	0.0003
0.0003	0.0003	0.0003	0.0003	0.0003	0.0031
0.0003	0.0003	0.0003	0.0003	0.0003	0.0003
0.0003	0.0132	0.0036	0.0003	0.0063	0.0003
0.0012	0.0003	0.0031	0.0003	0.0003	0.0003
0.0003	0.0017	0.0003	0.0003	0.0003	0.0003
0.0003	0.0003	0.0003	0.0003	0.0003	0.0003
0.0003	0.0003	0.0003	0.0003	0.0003	0.0003
0.1755	0.2369	0.1526	0.1509	0.1467	0.1478

CO 23 VEST TPPU + IL-1β 2 **194**	CO 23 VEST TPPU + IL-1β 3 **195**	CO 10 VULV TPPU + IL-1β 1 **16**	CO 10 VULV TPPU + IL-1β 2 **17**	CO 10 VULV TPPU + IL-1β 3 **18**	CO 21 VULV TPPU + IL-1β 1 **160**
0.0003	0.0003	0.0003	0.0003	0.0003	0.0003
0.0003	0.0003	0.0150	0.0003	0.0003	0.0003
0.0003	0.0003	0.0003	0.0003	0.0003	0.0003
0.0003	0.0003	0.0110	0.0105	0.0003	0.0003
2.7803	2.7140	0.0790	0.1025	0.0707	0.3049
0.0118	0.0063	0.0003	0.0003	0.0003	0.0003
0.0096	0.0003	0.0003	0.0003	0.0003	0.0003
0.0003	0.0003	0.0003	0.0003	0.0003	0.0003
0.0003	0.0003	0.0003	0.0003	0.0003	0.0003
154.9237	148.5972	5.6100	8.1528	6.3981	61.4722
0.1022	0.1114	0.0003	0.0003	0.0003	0.0612
0.3851	0.3524	0.0123	0.0139	0.0072	0.0003
0.0003	0.0003	0.0394	0.0003	0.0003	0.0003
0.0003	0.0003	0.0003	0.0003	0.0003	0.0003
0.0027	0.0024	0.0003	0.0003	0.0003	0.0003
0.0003	0.0003	0.0003	0.0003	0.0003	0.0316
5.4594	5.9736	0.0872	0.0967	0.0928	2.4395
152.8736	144.8578	5.3786	8.6927	6.1464	59.7470
0.0003	0.0003	0.0003	0.0003	0.0003	0.0003
0.0003	0.1364	0.0003	0.0244	0.0003	0.0003
0.0003	0.0003	0.0003	0.0003	0.0003	0.0003
0.0003	0.0003	0.0003	0.0003	0.0003	0.0003
0.0003	0.0003	0.0003	0.0003	0.0003	0.0003
0.0003	0.0003	0.0003	0.0003	0.0003	0.0003
0.1704	0.1456	0.0003	0.0003	0.0003	0.0551
0.0003	0.4352	0.0179	0.0003	0.0003	0.0003
0.0003	0.4892	0.0003	0.0058	0.0003	0.1113
0.0003	0.0003	0.0003	0.0003	0.0003	0.0003
0.0003	0.0003	0.0003	0.0003	0.0003	0.0003
0.0172	0.0337	0.0003	0.0003	0.0003	0.0003
0.0003	0.0003	0.0003	0.0069	0.0003	0.0003
0.0003	0.0515	0.0003	0.0003	0.0003	0.0088
0.0003	0.0003	0.0003	0.0003	0.0592	0.3387
0.0003	0.0003	0.0003	0.0112	0.0106	0.0093
0.0003	0.0003	0.0003	0.0003	0.0003	0.0003
0.0003	0.0003	0.0003	0.0003	0.0003	0.0003
0.0003	0.0003	0.0183	0.0003	0.0100	0.0003
0.0692	0.0681	0.0363	0.0719	0.0594	0.0648
0.0003	0.0003	0.0003	0.0003	0.0003	0.0003
0.0003	0.0003	0.0003	0.0003	0.0003	0.0003
0.0003	0.0003	0.0003	0.0003	0.0003	0.0072
0.0003	0.0003	0.0003	0.0003	0.0116	0.0150
0.0003	0.0003	0.0003	0.0003	0.0003	0.0003
0.0031	0.0040	0.0077	0.0022	0.0110	0.0067
0.0033	0.0134	0.0003	0.0032	0.0137	0.0217
0.0003	0.0003	0.0003	0.0003	0.0003	0.0003
0.0003	0.0003	0.0003	0.0003	0.0003	0.0003
0.0003	0.0003	0.0003	0.0003	0.0003	0.0003
0.0003	0.0003	0.0046	0.0003	0.0003	0.0003

0.0003	0.0003	0.0003	0.0003	0.0003	0.0003
0.0003	0.0003	0.0003	0.0003	0.0003	0.0003
0.0003	0.0003	0.0003	0.0003	0.0003	0.0003
1.4734	1.2841	0.7804	0.9797	0.7438	1.8457
0.0003	0.0003	0.0003	0.0003	0.0003	0.0003
0.0003	0.0003	0.0003	0.0003	0.0003	0.0003
0.0003	0.0003	0.0003	0.0003	0.0003	0.0003
0.0003	0.0003	0.0003	0.0003	0.0003	0.4366
0.0003	0.0003	0.0003	0.0583	0.0409	0.0003
0.0003	0.0003	0.0003	0.0003	0.0003	0.0003
0.0003	0.0003	0.0003	0.0003	0.0003	0.0003
0.0003	0.0003	0.0003	0.0003	0.0003	0.0003
0.3180	0.2753	0.0734	0.0985	0.1227	0.2174
0.0531	0.0003	0.0003	0.0003	0.0003	0.0003
0.5181	1.0886	0.0416	0.0003	0.0372	0.1765
0.0003	0.0003	0.0003	0.0003	0.0003	0.0003
0.2625	0.3054	0.0915	0.0003	0.1342	0.2538
0.0003	0.0003	0.0003	0.0003	0.0003	0.0003
0.0003	0.0003	0.0003	0.0003	0.0003	0.0003
0.1190	0.0972	0.0003	0.0257	0.0003	0.0650
0.0219	0.0269	0.0003	0.0003	0.0003	0.0003
0.0003	0.0003	0.0003	0.0003	0.0003	0.0003
0.0003	0.0003	0.0003	0.0003	0.0003	0.0003
0.0003	0.0003	0.0003	0.0003	0.0003	0.0003
0.0003	0.0003	0.0003	0.0003	0.0003	0.0003
0.0003	0.0003	0.0003	0.0003	0.0003	0.0003
0.0003	0.0003	0.0003	0.0003	0.0003	0.0003
0.0003	0.0003	0.0003	0.0003	0.0003	0.0003
0.0003	0.0003	0.0003	0.0003	0.0003	0.0003
0.0170	0.0191	0.0003	0.0003	0.0003	0.0178
0.0003	0.0003	0.0233	0.0003	0.0131	0.1238
0.0003	0.0003	0.0003	0.0124	0.0003	0.0112
0.0003	0.0003	0.0127	0.0003	0.0003	0.0003
0.0003	0.0003	0.0003	0.0003	0.0003	0.0003
0.0003	0.0003	0.0003	0.0003	0.0076	0.0003
0.1139	0.1389	0.1674	0.2112	0.2135	0.2078
0.0003	0.0003	0.0003	0.0003	0.0003	0.0003
0.1716	0.1970	0.1977	0.2598	0.3511	0.2547
0.0003	0.0003	0.0003	0.0003	0.0003	0.0003
0.0003	0.4417	0.0003	0.0003	0.0003	0.6670
0.0003	0.0003	0.0003	0.0003	0.0003	0.0003
0.0575	0.0466	0.0003	0.0003	0.0003	0.0188
23.6443	22.1550	1.9193	3.2022	2.1820	9.1220
5.7592	5.0020	0.3154	0.5036	0.3596	1.6913
0.0003	0.0003	0.0003	0.0003	0.0003	0.0003
1.4936	1.4292	0.0434	0.0504	0.0502	0.7666
0.2653	0.1986	0.0003	0.0144	0.0106	0.1525
0.0003	0.0003	0.0003	0.0003	0.0003	0.0003
0.0117	0.0146	0.0003	0.0003	0.0003	0.0003
0.0003	0.0003	0.0076	0.0003	0.0003	0.0003
0.0003	0.0003	0.0003	0.0003	0.0003	0.0003
0.0003	0.0003	0.0003	0.0003	0.0003	0.0003
0.1287	0.1335	0.0303	0.0308	0.0253	0.0722

0.0003	0.0003	0.0003	0.0003	0.0003	0.0003
0.0003	0.0003	0.0003	0.0003	0.0003	0.0003
0.0003	0.0003	0.0003	0.0003	0.0003	0.0003
0.0057	0.0003	0.0003	0.0003	0.0003	0.0003
0.0152	0.0152	0.0003	0.0003	0.0003	0.0206
0.0290	0.0245	0.0074	0.0098	0.0107	0.0360
0.0003	0.0003	0.0003	0.0003	0.0003	0.0003
0.0003	0.0003	0.0003	0.0003	0.0003	0.0003
0.0401	0.0003	0.0003	0.0003	0.1474	0.0003
0.0003	0.0003	0.0003	0.0003	0.0275	0.0003
0.0003	0.0003	0.0003	0.0003	0.0003	0.0003
0.0286	0.0369	0.0003	0.0003	0.0122	0.0203
0.0003	0.0003	0.0003	0.0003	0.0003	0.0003
0.0003	0.0003	0.0003	0.0003	0.0003	0.0003
0.0003	0.0310	0.0003	0.0003	0.0003	0.0003
0.0003	0.0003	0.0003	0.0003	0.0003	0.0003
0.0003	0.0003	0.0003	0.0208	0.0003	0.0003
0.0003	0.0003	0.0003	0.0003	0.0003	0.0003
0.0003	0.0003	0.0003	0.0003	0.0003	0.0003
2.9102	2.6782	0.0907	0.0975	0.0791	0.6895
0.0380	0.0003	0.0003	0.0003	0.0003	0.0003
0.0279	0.0003	0.0003	0.0181	0.0003	0.0003
0.0003	0.0003	0.0530	0.1517	0.0003	0.0003
0.0304	0.0003	0.0003	0.0003	0.0003	0.0003
0.0003	0.0003	0.0003	0.0003	0.0003	0.0003
0.0003	0.0003	0.0003	0.0003	0.0003	0.0003
0.0003	0.0003	0.0003	0.0003	0.0003	0.0003
0.0003	0.0003	0.0003	0.0003	0.0003	0.0480
0.0095	0.0003	0.0081	0.0085	0.0003	0.0180
0.0003	0.0003	0.0003	0.0003	0.0003	0.0003
0.0003	0.0003	0.0003	0.0003	0.0003	0.0003
0.0041	0.0003	0.0135	0.0003	0.0003	0.0082
0.0003	0.0003	0.0003	0.0003	0.0003	0.0003
0.0003	0.0158	0.0003	0.0003	0.0058	0.0003
0.0003	0.0003	0.0003	0.0003	0.0003	0.0003
0.0102	0.0102	0.0003	0.0003	0.0003	0.0003
0.0003	0.0003	0.0003	0.0003	0.0003	0.0003
0.0049	0.0003	0.0003	0.0003	0.0003	0.0003
0.0003	0.0003	0.0003	0.0003	0.0003	0.0003
0.0003	0.0003	0.0324	0.0112	0.0173	0.0003
0.0003	0.0003	0.0024	0.0003	0.0003	0.0003
0.0003	0.0003	0.0028	0.0003	0.0003	0.0003
0.0003	0.0003	0.0003	0.0003	0.0003	0.0003
0.0003	0.0003	0.0003	0.0003	0.0003	0.0003
0.1507	0.1670	0.2578	0.2363	0.1572	0.2044

CO 21 VULV TPPU + IL-1β 2 **161**	CO 21 VULV TPPU + IL-1β 3 **162**	CO 23 VULV TPPU + IL-1β 1 **196**	CO 23 VULV TPPU + IL-1β 2 **197**	CO 23 VULV TPPU + IL-1β 3 **198**	CA 13 VEST TPPU + IL-1β 1 **49**
0.0003	0.0003	0.0003	0.0003	0.0003	0.0003
0.0003	0.0003	0.0003	0.0003	0.0003	0.0003
0.0003	0.0003	0.0003	0.0003	0.0003	0.0003
0.0003	0.0003	0.0003	0.0003	0.0003	0.0003
0.4211	0.4713	0.4881	0.4359	0.3929	0.0003
0.0085	0.0064	0.0120	0.0003	0.0040	0.0003
0.0003	0.0003	0.0003	0.0003	0.0003	0.0003
0.0003	0.0003	0.0003	0.0003	0.0003	0.0003
0.0003	0.0003	0.0003	0.0003	0.0003	0.0003
36.4148	39.7068	42.9648	40.4867	34.4104	42.9815
0.0332	0.0400	0.0226	0.0295	0.0185	0.0405
0.0003	0.0462	0.0604	0.0559	0.0003	0.0657
0.0380	0.0003	0.0003	0.0003	0.0003	0.0003
0.0003	0.0003	0.0003	0.0003	0.0003	0.0003
0.0003	0.0003	0.0003	0.0003	0.0003	0.0003
0.0407	0.0177	0.0003	0.0003	0.0003	0.0003
1.2473	1.5032	1.3061	1.6275	1.1460	1.8676
36.2358	38.8725	42.5559	41.4609	33.0184	44.6303
0.0003	0.0003	0.0003	0.0003	0.0003	0.0003
0.0003	0.0003	0.0212	0.0003	0.0003	0.0003
0.0003	0.0003	0.0003	0.0003	0.0003	0.0003
0.0122	0.0003	0.0003	0.0003	0.0003	0.0003
0.0003	0.0003	0.0003	0.0003	0.0003	0.0003
0.0003	0.0003	0.0003	0.0003	0.0003	0.0003
0.0003	0.0003	0.0003	0.0290	0.0202	0.0445
0.0003	0.0643	0.0003	0.0586	0.0003	0.0003
0.0003	0.0003	0.0003	0.0003	0.0003	0.0003
0.0003	0.0003	0.0003	0.0003	0.0003	0.0003
0.0003	0.0003	0.0003	0.0003	0.0003	0.0003
0.0003	0.0003	0.0003	0.0003	0.0003	0.0003
0.0066	0.0003	0.0003	0.0003	0.0003	0.0003
0.0003	0.0003	0.0003	0.0003	0.0003	0.0003
0.0003	0.0003	0.2467	0.1877	0.0003	0.0359
0.0003	0.0003	0.0003	0.0003	0.0003	0.0108
0.0003	0.0003	0.0016	0.0003	0.0003	0.0003
0.0095	0.0003	0.0121	0.0088	0.0072	0.0003
0.0003	0.0087	0.0003	0.0104	0.0003	0.0003
0.0421	0.0584	0.0696	0.0582	0.0485	0.0680
0.0003	0.0003	0.0003	0.0003	0.0003	0.0003
0.0003	0.0003	0.0003	0.0003	0.0003	0.0003
0.0067	0.0003	0.0003	0.0003	0.0003	0.0003
0.0003	0.0193	0.0003	0.0003	0.0042	0.0003
0.0003	0.0003	0.0003	0.0003	0.0014	0.0003
0.0048	0.0031	0.0039	0.0052	0.0041	0.0032
0.0003	0.0241	0.0043	0.0139	0.0028	0.0003
0.0003	0.0003	0.0003	0.0003	0.0003	0.0003
0.0003	0.0003	0.0003	0.0003	0.0003	0.0003
0.0003	0.0003	0.0003	0.0003	0.0003	0.0003
0.0003	0.0128	0.0003	0.0080	0.0095	0.0048

0.0003	0.0003	0.0003	0.0003	0.0003	0.0003
0.0003	0.0066	0.0003	0.0003	0.0040	0.0003
0.0003	0.0003	0.0003	0.0003	0.0003	0.0003
1.4571	1.5118	0.8100	1.1950	0.9344	0.8166
0.0003	0.0003	0.0003	0.0003	0.0003	0.0003
0.0003	0.0003	0.0003	0.0003	0.0003	0.0003
0.0003	0.0003	0.0003	0.0003	0.0003	0.0003
0.0003	0.0003	0.0003	0.3302	0.0003	0.2212
0.0003	0.0314	0.0003	0.0003	0.0066	0.0407
0.0003	0.0003	0.0003	0.0003	0.0003	0.0003
0.0003	0.0003	0.0003	0.0003	0.0003	0.0003
0.0003	0.0003	0.0003	0.0003	0.0003	0.0003
0.1850	0.1813	0.2040	0.1570	0.1756	0.1588
0.0003	0.0003	0.0134	0.0003	0.0164	0.0003
0.1292	0.1229	0.1615	0.1237	0.3097	0.1521
0.0003	0.0003	0.0003	0.0003	0.0003	0.0003
0.1488	0.1562	0.2626	0.2357	0.2933	0.1424
0.0003	0.0003	0.0003	0.0003	0.0003	0.0003
0.0003	0.0003	0.0003	0.0003	0.0003	0.0003
0.0658	0.0579	0.0273	0.0315	0.0252	0.0003
0.0003	0.0003	0.0003	0.0003	0.0003	0.0003
0.0003	0.0003	0.0003	0.0003	0.0003	0.0003
0.0003	0.0003	0.0003	0.0003	0.0003	0.0003
0.0003	0.0003	0.0003	0.0003	0.0003	0.0003
0.0003	0.0003	0.0003	0.0003	0.0003	0.0003
0.0003	0.0003	0.0003	0.0003	0.0003	0.0003
0.0003	0.0003	0.0003	0.0003	0.0003	0.0003
0.0003	0.0003	0.0003	0.0003	0.0003	0.0003
0.0003	0.0003	0.0003	0.0003	0.0003	0.0003
0.0003	0.0003	0.0135	0.0047	0.0003	0.0189
0.0517	0.0003	0.0453	0.0003	0.0003	0.0003
0.0120	0.0003	0.0003	0.0161	0.0003	0.0003
0.0003	0.0003	0.0003	0.0003	0.0003	0.0003
0.0003	0.0003	0.0003	0.0003	0.0003	0.0003
0.0050	0.0003	0.0003	0.0003	0.0003	0.0095
0.1463	0.1231	0.1448	0.1084	0.1193	0.2106
0.0003	0.0003	0.0003	0.0003	0.0003	0.0003
0.1291	0.2713	0.0003	0.2253	0.1977	0.3712
0.0003	0.0003	0.0003	0.0003	0.0203	0.0003
0.0003	0.5447	0.0003	0.0003	0.0003	0.0003
0.0003	0.0035	0.0003	0.0003	0.0003	0.0003
0.0156	0.0286	0.0418	0.0313	0.0567	0.0377
4.7317	7.4755	8.2934	8.0781	8.0977	7.6852
0.9760	1.3117	1.7902	1.6241	1.6501	1.5660
0.0003	0.0003	0.0003	0.0003	0.0003	0.0003
0.4293	0.5864	0.5419	0.5286	0.5462	0.4965
0.0813	0.0999	0.1004	0.0958	0.0865	0.0757
0.0003	0.0003	0.0003	0.0003	0.0003	0.0003
0.0003	0.0003	0.0003	0.0003	0.0003	0.0003
0.0003	0.0003	0.0003	0.0003	0.0003	0.0003
0.0003	0.0003	0.0003	0.0003	0.0003	0.0003
0.0003	0.0003	0.0003	0.0003	0.0003	0.0003
0.0556	0.0630	0.0495	0.0530	0.0603	0.0857

0.0003	0.0003	0.0003	0.0003	0.0003	0.0003
0.0003	0.0003	0.0003	0.0003	0.0003	0.0003
0.0003	0.0003	0.0003	0.0003	0.0003	0.0003
0.0003	0.0003	0.0003	0.0003	0.0003	0.0003
0.0121	0.0262	0.0003	0.0003	0.0099	0.0114
0.0241	0.0292	0.0158	0.0159	0.0136	0.0097
0.0003	0.0003	0.0003	0.0003	0.0003	0.0003
0.0003	0.0003	0.0003	0.0003	0.0003	0.0048
0.0003	0.0461	0.0309	0.0270	0.0003	0.0340
0.0003	0.0003	0.0003	0.0003	0.0117	0.0003
0.0003	0.0003	0.0003	0.0003	0.0003	0.0003
0.0119	0.0120	0.0003	0.0003	0.0003	0.0308
0.0003	0.0003	0.0003	0.0003	0.0003	0.0003
0.0003	0.0003	0.0003	0.0003	0.0282	0.0003
0.0003	0.0256	0.0003	0.0003	0.0003	0.0003
0.0284	0.0003	0.0003	0.0003	0.0207	0.0003
0.0003	0.0003	0.0003	0.0003	0.0003	0.0003
0.0003	0.0003	0.0003	0.0003	0.0003	0.0003
0.0003	0.0003	0.0003	0.0003	0.0003	0.0003
0.4895	0.5296	0.4576	0.4786	0.4506	0.5507
0.0003	0.0003	0.0003	0.0003	0.0003	0.0003
0.0120	0.0384	0.0003	0.0003	0.0003	0.0473
0.0003	0.0782	0.0003	0.0003	0.0003	0.0003
0.0003	0.0003	0.0003	0.0003	0.0003	0.0003
0.0003	0.0003	0.0003	0.0003	0.0003	0.0003
0.0003	0.0003	0.0003	0.0003	0.0003	0.0003
0.0003	0.0003	0.0003	0.0003	0.0003	0.0003
0.0003	0.0003	0.0003	0.0736	0.0003	0.0003
0.0090	0.0100	0.0003	0.0003	0.0003	0.0223
0.0003	0.0003	0.0003	0.0003	0.0003	0.0003
0.0003	0.0003	0.0003	0.0003	0.0003	0.0003
0.0003	0.0003	0.0003	0.0171	0.0003	0.0003
0.0003	0.0003	0.0003	0.0125	0.0003	0.0003
0.0003	0.0003	0.0003	0.0003	0.0003	0.0003
0.0003	0.0003	0.0003	0.0003	0.0003	0.0003
0.0003	0.0003	0.0003	0.0003	0.0003	0.0003
0.0003	0.0003	0.0003	0.0003	0.0003	0.0003
0.0003	0.0003	0.0003	0.0003	0.0003	0.0021
0.0003	0.0003	0.0003	0.0003	0.0003	0.0003
0.0221	0.0003	0.0003	0.0003	0.0003	0.0094
0.0003	0.0034	0.0003	0.0003	0.0003	0.0003
0.0023	0.0003	0.0008	0.0003	0.0003	0.0003
0.0003	0.0003	0.0003	0.0003	0.0003	0.0003
0.0003	0.0003	0.0003	0.0003	0.0003	0.0003
0.1379	0.1977	0.1565	0.1954	0.1445	0.2877

CA 13 VEST TPPU + IL-1β 2 **50**	CA 13 VEST TPPU + IL-1β 3 **51**	CA 14 VEST TPPU + IL-1β 1 **85**	CA 14 VEST TPPU + IL-1β 2 **86**	CA 14 VEST TPPU + IL-1β 3 **87**	CA 20 VEST TPPU + IL-1β 1 **121**	CA 20 VEST TPPU + IL-1β 2 **122**
0.0003	0.0003	0.0003	0.0003	0.0003	0.0030	0.0003
0.0003	0.0003	0.0003	0.0003	0.0003	0.0003	0.0003
0.0003	0.0003	0.0003	0.0003	0.0003	0.0003	0.0003
0.0003	0.0003	0.0003	0.0003	0.0003	0.0003	0.0003
0.5185	0.0003	0.0452	0.1332	0.0003	0.7677	0.8492
0.0003	0.0088	0.0003	0.0037	0.0003	0.0109	0.0003
0.0003	0.0003	0.0003	0.0003	0.0003	0.0003	0.0003
0.0003	0.0003	0.0003	0.0003	0.0003	0.0003	0.0003
0.0003	0.0003	0.0003	0.0003	0.0003	0.0003	0.0003
45.1245	57.1062	13.5277	11.8905	10.9536	64.7265	66.6829
0.0325	0.0459	0.0003	0.0003	0.0003	0.0376	0.0373
0.0003	0.0946	0.0003	0.0003	0.0003	0.1278	0.1138
0.0003	0.0003	0.0003	0.0003	0.0003	0.0003	0.0003
0.0003	0.0003	0.0003	0.0003	0.0003	0.0003	0.0003
0.0013	0.0003	0.0003	0.0003	0.0003	0.0003	0.0003
0.0003	0.0003	0.0287	0.0242	0.0003	0.0003	0.0248
1.9224	2.3042	0.4723	0.4688	0.4338	1.5839	2.0645
43.4991	53.4150	12.6870	12.1852	11.4567	64.8770	67.7418
0.0003	0.0003	0.0003	0.0003	0.0003	0.0003	0.0003
0.0003	0.0003	0.0003	0.0193	0.0003	0.0500	0.0394
0.0003	0.0003	0.0003	0.0003	0.0003	0.0003	0.0003
0.0003	0.0003	0.0365	0.0003	0.0134	0.0003	0.0003
0.0003	0.0003	0.0003	0.0003	0.0003	0.0003	0.0003
0.0003	0.0003	0.0003	0.0003	0.0003	0.0003	0.0003
0.0552	0.0571	0.0003	0.0003	0.0003	0.0450	0.0476
0.0003	0.0003	0.0003	0.0003	0.0317	0.0003	0.0003
0.0003	0.0003	0.0003	0.0003	0.0207	0.0003	0.0003
0.0003	0.0003	0.0003	0.0003	0.0003	0.0003	0.0003
0.0003	0.0003	0.0003	0.0003	0.0003	0.0003	0.0003
0.0003	0.0003	0.0003	0.0003	0.0496	0.0003	0.0003
0.0003	0.0003	0.0003	0.0003	0.0003	0.0003	0.0003
0.0003	0.0003	0.0003	0.0003	0.0003	0.0187	0.0003
0.0354	0.0942	0.0430	0.0003	0.0003	0.0003	0.0003
0.0064	0.0003	0.0003	0.0076	0.0228	0.0003	0.0003
0.0003	0.0003	0.0003	0.0003	0.0003	0.0003	0.0003
0.0003	0.0003	0.0086	0.0003	0.0003	0.0003	0.0003
0.0129	0.0003	0.0003	0.0100	0.0003	0.0003	0.0003
0.0630	0.0958	0.0548	0.0692	0.0489	0.0433	0.0510
0.0003	0.0003	0.0003	0.0003	0.0003	0.0003	0.0003
0.0003	0.0003	0.0003	0.0003	0.0003	0.0003	0.0003
0.0048	0.0089	0.0003	0.0003	0.0003	0.0003	0.0003
0.0003	0.0003	0.0067	0.0003	0.0003	0.0003	0.0122
0.0003	0.0003	0.0003	0.0003	0.0003	0.0003	0.0003
0.0022	0.0016	0.0018	0.0042	0.0061	0.0128	0.0158
0.0085	0.0028	0.0109	0.0226	0.0067	0.0115	0.0104
0.0003	0.0003	0.0003	0.0003	0.0003	0.0003	0.0003
0.0003	0.0003	0.0003	0.0003	0.0003	0.0003	0.0003
0.0003	0.0003	0.0003	0.0003	0.0003	0.0003	0.0003
0.0058	0.0148	0.0003	0.0003	0.0003	0.0049	0.0003

0.0003	0.0003	0.0003	0.0003	0.0003	0.0003	0.0003
0.0003	0.0003	0.0003	0.0003	0.0003	0.0003	0.0003
0.0003	0.0003	0.0003	0.0003	0.0003	0.0003	0.0003
0.8623	0.7159	1.7236	2.2063	1.4645	2.1869	1.8578
0.0003	0.0003	0.0003	0.0003	0.0003	0.0003	0.0003
0.0003	0.0003	0.0003	0.0003	0.0003	0.0003	0.0003
0.0003	0.0003	0.0003	0.0003	0.0003	0.0003	0.0003
0.0003	0.0003	0.5837	0.0003	0.0003	0.0003	0.2720
0.0003	0.0368	0.0003	0.0003	0.0003	0.0003	0.0003
0.0003	0.0003	0.0003	0.0003	0.0003	0.0003	0.0003
0.0003	0.0003	0.0003	0.0003	0.0003	0.0003	0.0003
0.0003	0.0003	0.0003	0.0003	0.0003	0.0003	0.0003
0.1630	0.1363	0.1085	0.0934	0.1015	0.0728	0.1203
0.0003	0.0266	0.0003	0.0003	0.0003	0.0003	0.0003
0.1481	0.3825	0.0667	0.0003	0.0003	0.1008	0.1264
0.0003	0.0003	0.0003	0.0003	0.0003	0.0003	0.0003
0.2132	0.1706	0.0856	0.1388	0.0984	0.1108	0.1081
0.0003	0.0003	0.0003	0.0003	0.0003	0.0003	0.0003
0.0003	0.0003	0.0003	0.0003	0.0003	0.0003	0.0003
0.0003	0.0003	0.0003	0.0003	0.0317	0.0467	0.0710
0.0003	0.0003	0.0003	0.0003	0.0003	0.0003	0.0003
0.0003	0.0003	0.0003	0.0003	0.0003	0.0003	0.0003
0.0003	0.0003	0.0003	0.0003	0.0212	0.0003	0.0003
0.0003	0.0003	0.0003	0.0003	0.0003	0.0003	0.0003
0.0003	0.0003	0.0003	0.0003	0.0003	0.0003	0.0003
0.0003	0.0003	0.0003	0.0003	0.0003	0.0003	0.0003
0.0003	0.0003	0.0003	0.0003	0.0003	0.0003	0.0003
0.0003	0.0003	0.0003	0.0003	0.0003	0.0003	0.0003
0.0003	0.0003	0.0003	0.0003	0.0003	0.0003	0.0003
0.0003	0.0175	0.0003	0.0003	0.0125	0.0141	0.0003
0.0003	0.0514	0.0361	0.0415	0.0003	0.0003	0.0815
0.0102	0.0083	0.0003	0.0003	0.0162	0.0003	0.0003
0.0003	0.0003	0.0003	0.0003	0.0003	0.0159	0.0003
0.0003	0.0003	0.0003	0.0003	0.0003	0.0003	0.0003
0.0003	0.0003	0.0062	0.0126	0.0003	0.0003	0.0003
0.2551	0.2147	0.1167	0.2396	0.1108	0.1361	0.1511
0.0003	0.0003	0.0003	0.0003	0.0003	0.0003	0.0003
0.4685	0.3549	0.0992	0.2527	0.1927	0.2330	0.3001
0.0003	0.0003	0.0003	0.0003	0.0003	0.1164	0.0003
0.0003	0.0003	0.0003	0.8844	0.7125	1.5394	0.0003
0.0003	0.0003	0.0003	0.0003	0.0003	0.0003	0.0015
0.0273	0.0209	0.0166	0.0198	0.0166	0.0253	0.0282
7.0811	8.3364	3.5842	4.1118	3.3781	6.5880	7.9196
1.4935	1.7625	0.6476	0.6970	0.6244	1.4200	1.6012
0.0003	0.0003	0.0003	0.0003	0.0003	0.0003	0.0003
0.4481	0.4951	0.2487	0.2910	0.2475	0.4476	0.6394
0.0691	0.1086	0.0601	0.0850	0.0445	0.1028	0.1244
0.0003	0.0003	0.0003	0.0003	0.0003	0.0003	0.0003
0.0003	0.0003	0.0003	0.0003	0.0003	0.0003	0.0003
0.0003	0.0003	0.0003	0.0114	0.0047	0.0074	0.0003
0.0003	0.0003	0.0003	0.0003	0.0003	0.0003	0.0003
0.0003	0.0003	0.0003	0.0003	0.0003	0.0003	0.0003
0.0892	0.0607	0.0411	0.0390	0.0405	0.0352	0.0595

0.0003	0.0003	0.0003	0.0003	0.0003	0.0232	0.0003
0.0003	0.0003	0.0003	0.0003	0.0003	0.0003	0.0003
0.0003	0.0003	0.0003	0.0003	0.0003	0.0003	0.0003
0.0003	0.0003	0.0003	0.0003	0.0003	0.0003	0.0003
0.0003	0.0003	0.0003	0.0158	0.0003	0.0003	0.0003
0.0109	0.0140	0.0092	0.0136	0.0166	0.0164	0.0190
0.0003	0.0003	0.0003	0.0003	0.0003	0.0003	0.0003
0.0003	0.0003	0.0076	0.0003	0.0003	0.0003	0.0003
0.0003	0.0286	0.0165	0.0003	0.0003	0.0289	0.0003
0.0003	0.0003	0.0003	0.0003	0.0003	0.0003	0.0003
0.0003	0.0003	0.0003	0.0003	0.0003	0.0003	0.0003
0.0125	0.0129	0.0064	0.0144	0.0003	0.0185	0.0239
0.0003	0.0003	0.0003	0.0003	0.0003	0.0003	0.0003
0.0003	0.0003	0.0003	0.0003	0.0003	0.0003	0.0003
0.0003	0.0003	0.0003	0.0003	0.0003	0.0003	0.0003
0.0003	0.0003	0.0003	0.0003	0.0322	0.0003	0.0003
0.0003	0.0140	0.0003	0.0003	0.0003	0.0003	0.0003
0.0003	0.0003	0.0003	0.0003	0.0003	0.0185	0.0003
0.0003	0.0003	0.0003	0.0003	0.0003	0.0003	0.0003
0.5079	0.5583	0.1621	0.1255	0.1106	0.8205	0.8226
0.0003	0.0003	0.0003	0.0003	0.0003	0.0003	0.0003
0.0393	0.0263	0.0003	0.0343	0.0003	0.0003	0.0003
0.0003	0.0370	0.0003	0.0003	0.0506	0.0003	0.0186
0.0003	0.0003	0.0003	0.0003	0.0003	0.0003	0.0003
0.0003	0.0003	0.0003	0.0003	0.0003	0.0003	0.0003
0.0003	0.0003	0.0003	0.0003	0.0003	0.0003	0.0003
0.0003	0.0003	0.0003	0.0003	0.0003	0.0003	0.0003
0.0003	0.0003	0.0003	0.0003	0.0003	0.0003	0.0003
0.0003	0.0003	0.0003	0.0003	0.0003	0.0206	0.0003
0.0003	0.0003	0.0003	0.0003	0.0003	0.0003	0.0003
0.0003	0.0003	0.0003	0.0003	0.0003	0.0003	0.0003
0.0003	0.0003	0.0003	0.0149	0.0003	0.0003	0.0086
0.0003	0.0003	0.0003	0.0003	0.0003	0.0003	0.0003
0.0144	0.0124	0.0157	0.0187	0.0003	0.0003	0.0003
0.0003	0.0003	0.0003	0.0003	0.0003	0.0003	0.0003
0.0056	0.0003	0.0003	0.0003	0.0003	0.0003	0.0003
0.0003	0.0003	0.0003	0.0003	0.0003	0.0003	0.0003
0.0003	0.0003	0.0003	0.0003	0.0003	0.0003	0.0003
0.0003	0.0003	0.0003	0.0003	0.0003	0.0003	0.0003
0.0003	0.0003	0.0370	0.0003	0.0159	0.0003	0.0136
0.0003	0.0003	0.0037	0.0096	0.0041	0.0097	0.0038
0.0005	0.0036	0.0027	0.0038	0.0025	0.0012	0.0017
0.0003	0.0003	0.0003	0.0003	0.0003	0.0003	0.0003
0.0003	0.0003	0.0003	0.0003	0.0003	0.0003	0.0003
0.3345	0.3296	0.1850	0.2155	0.1544	0.1401	0.1441

CA 20 VEST TPPU + IL-1β 3 **123**	CA 13 VULV TPPU + IL-1β 1 **52**	CA 13 VULV TPPU + IL-1β 2 **53**	CA 13 VULV TPPU + IL-1β 3 **54**	CA 14 VULV TPPU + IL-1β 1 **88**	CA 14 VULV TPPU + IL-1β 2 **89**
0.0003	0.0003	0.0003	0.0003	0.0003	0.0003
0.0003	0.0003	0.0003	0.0003	0.0003	0.0003
0.0003	0.0003	0.0003	0.0003	0.0003	0.0003
0.0003	0.0003	0.0003	0.0003	0.0003	0.0003
0.7171	0.3252	0.8586	0.2905	0.4826	0.6087
0.0077	0.0075	0.0003	0.0003	0.0003	0.0003
0.0003	0.0003	0.0003	0.0003	0.0003	0.0003
0.0003	0.0003	0.0003	0.0003	0.0003	0.0003
0.0003	0.0003	0.0003	0.0003	0.0003	0.0003
66.9523	34.1741	30.8619	29.4744	47.1078	49.4469
0.0259	0.0257	0.0203	0.0236	0.0242	0.0325
0.1201	0.0003	0.0003	0.0003	0.0621	0.0003
0.0003	0.0266	0.0003	0.0577	0.0003	0.0003
0.0003	0.0003	0.0003	0.0003	0.0003	0.0003
0.0030	0.0003	0.0003	0.0003	0.0003	0.0003
0.0003	0.0003	0.0274	0.0330	0.0003	0.0003
1.8837	1.2107	0.9744	0.9207	1.3400	1.4287
64.4468	33.3811	30.4386	28.8167	47.1823	50.2310
0.0003	0.0003	0.0003	0.0003	0.0003	0.0003
0.0470	0.0003	0.0003	0.0003	0.0003	0.0349
0.0003	0.0003	0.0003	0.0003	0.0003	0.0003
0.0003	0.0003	0.0003	0.0003	0.0201	0.0003
0.0003	0.0003	0.0003	0.0003	0.0003	0.0003
0.0003	0.0003	0.0003	0.0003	0.0003	0.0003
0.0564	0.0003	0.0003	0.0003	0.0381	0.0003
0.0003	0.0003	0.0292	0.0003	0.0631	0.0768
0.0003	0.0003	0.0003	0.0003	0.0003	0.0003
0.0003	0.0003	0.0003	0.0003	0.0003	0.0003
0.0003	0.0003	0.0003	0.0003	0.0003	0.0003
0.0003	0.0271	0.0003	0.0003	0.0003	0.0003
0.0003	0.0003	0.0003	0.0003	0.0003	0.0067
0.0003	0.0115	0.0003	0.0003	0.0003	0.0003
0.0003	0.0003	0.1120	0.0596	0.0003	0.0003
0.0003	0.0003	0.0097	0.0003	0.0099	0.0136
0.0003	0.0003	0.0003	0.0003	0.0003	0.0003
0.0003	0.0003	0.0003	0.0003	0.0087	0.0076
0.0003	0.0003	0.0003	0.0003	0.0003	0.0003
0.0003	0.0649	0.0484	0.0456	0.0700	0.0498
0.0003	0.0003	0.0003	0.0003	0.0003	0.0003
0.0003	0.0003	0.0003	0.0003	0.0003	0.0003
0.0003	0.0059	0.0069	0.0003	0.0154	0.0049
0.0003	0.0054	0.0003	0.0003	0.0003	0.0173
0.0003	0.0003	0.0003	0.0003	0.0003	0.0003
0.0126	0.0043	0.0030	0.0014	0.0026	0.0045
0.0157	0.0097	0.0060	0.0088	0.0061	0.0072
0.0003	0.0003	0.0003	0.0003	0.0003	0.0003
0.0003	0.0003	0.0003	0.0003	0.0003	0.0003
0.0003	0.0003	0.0003	0.0003	0.0003	0.0003
0.0044	0.0033	0.0003	0.0053	0.0060	0.0003

0.0003	0.0003	0.0003	0.0003	0.0003	0.0003
0.0003	0.0003	0.0003	0.0003	0.0003	0.0003
0.0003	0.0003	0.0003	0.0003	0.0003	0.0003
2.0042	0.9401	0.8571	0.8313	1.6016	1.6035
0.0003	0.0003	0.0003	0.0003	0.0003	0.0003
0.0003	0.0003	0.0003	0.0003	0.0003	0.0003
0.0003	0.0003	0.0003	0.0003	0.0003	0.0003
0.2498	0.0003	0.2878	0.0003	0.0003	0.0003
0.0003	0.0003	0.0505	0.0488	0.0003	0.0003
0.0003	0.0003	0.0003	0.0003	0.0003	0.0003
0.0003	0.0003	0.0003	0.0003	0.0003	0.0003
0.0003	0.0003	0.0003	0.0003	0.0003	0.0003
0.0832	0.1305	0.1149	0.1048	0.1578	0.1375
0.0003	0.0003	0.0003	0.0243	0.0003	0.0003
0.0573	0.2866	0.1191	0.1241	0.1802	0.1542
0.0003	0.0003	0.0003	0.0003	0.0003	0.0003
0.1192	0.1698	0.1294	0.1124	0.1965	0.2412
0.0003	0.0003	0.0003	0.0003	0.0003	0.0003
0.0003	0.0003	0.0003	0.0003	0.0003	0.0003
0.0350	0.0003	0.0003	0.0348	0.0276	0.0385
0.0003	0.0003	0.0003	0.0003	0.0003	0.0003
0.0003	0.0003	0.0003	0.0003	0.0003	0.0003
0.0003	0.0003	0.0003	0.0003	0.0003	0.0003
0.0003	0.0003	0.0003	0.0003	0.0003	0.0003
0.0003	0.0003	0.0003	0.0003	0.0003	0.0003
0.0003	0.0003	0.0003	0.0003	0.0003	0.0003
0.0003	0.0003	0.0003	0.0003	0.0003	0.0003
0.0003	0.0003	0.0003	0.0003	0.0003	0.0003
0.0003	0.0003	0.0003	0.0003	0.0003	0.0003
0.0003	0.0003	0.0266	0.0177	0.0003	0.0003
0.0003	0.0003	0.0003	0.0003	0.0003	0.0003
0.0108	0.0003	0.0003	0.0003	0.0191	0.0003
0.0003	0.0003	0.0003	0.0003	0.0003	0.0003
0.0003	0.0003	0.0003	0.0003	0.0003	0.0003
0.0003	0.0049	0.0003	0.0082	0.0003	0.0003
0.1249	0.2571	0.2523	0.3175	0.1261	0.1225
0.0003	0.0003	0.0003	0.0003	0.0003	0.0003
0.2616	0.4018	0.4035	0.4376	0.0966	0.1215
0.0003	0.0003	0.0003	0.0003	0.0003	0.0435
0.8428	0.0003	0.0003	0.0003	0.8645	0.0003
0.0003	0.0012	0.0003	0.0034	0.0003	0.0003
0.0202	0.0296	0.0383	0.0308	0.0284	0.0352
7.3436	6.8963	5.7737	5.7281	8.6484	8.9834
1.7661	1.2315	1.0853	1.0869	1.9821	2.0309
0.0003	0.0003	0.0003	0.0003	0.0003	0.0003
0.5450	0.4034	0.3441	0.3122	0.4356	0.5128
0.1414	0.0540	0.0637	0.0433	0.0707	0.1424
0.0003	0.0003	0.0003	0.0003	0.0003	0.0003
0.0003	0.0003	0.0003	0.0003	0.0003	0.0003
0.0003	0.0083	0.0003	0.0119	0.0003	0.0003
0.0003	0.0003	0.0003	0.0003	0.0003	0.0003
0.0003	0.0003	0.0003	0.0003	0.0003	0.0003
0.0441	0.0708	0.0543	0.0003	0.0531	0.0699

0.0003	0.0003	0.0003	0.0003	0.0003	0.0003
0.0003	0.0003	0.0003	0.0003	0.0003	0.0003
0.0003	0.0003	0.0003	0.0003	0.0003	0.0003
0.0003	0.0003	0.0003	0.0003	0.0003	0.0003
0.0003	0.0003	0.0003	0.0003	0.0003	0.0121
0.0198	0.0103	0.0103	0.0106	0.0141	0.0153
0.0003	0.0003	0.0003	0.0003	0.0003	0.0003
0.0003	0.0003	0.0003	0.0003	0.0003	0.0003
0.0160	0.0003	0.0003	0.0530	0.0607	0.0458
0.0003	0.0003	0.0003	0.0246	0.0003	0.0003
0.0003	0.0003	0.0003	0.0003	0.0003	0.0003
0.0149	0.0110	0.0110	0.0098	0.0122	0.0167
0.0003	0.0003	0.0003	0.0003	0.0003	0.0003
0.0003	0.0003	0.0003	0.0003	0.0003	0.0003
0.0003	0.0003	0.0003	0.0003	0.0003	0.0003
0.0003	0.0003	0.0003	0.0003	0.0003	0.0003
0.0210	0.0003	0.0003	0.0003	0.0003	0.0003
0.0003	0.0003	0.0003	0.0003	0.0003	0.0003
0.0003	0.0003	0.0003	0.0003	0.0003	0.0003
0.7798	0.3985	0.3559	0.3503	0.5600	0.5140
0.0003	0.0003	0.0003	0.0003	0.0003	0.0003
0.0285	0.0003	0.0138	0.0241	0.0003	0.0003
0.0336	0.0003	0.0293	0.0633	0.0180	0.0003
0.0003	0.0003	0.0003	0.0003	0.0003	0.0003
0.0003	0.0003	0.0003	0.0003	0.0003	0.0003
0.0003	0.0003	0.0003	0.0003	0.0003	0.0003
0.0003	0.0003	0.0003	0.0003	0.0003	0.0003
0.0003	0.0003	0.0003	0.0560	0.0003	0.0003
0.0003	0.0093	0.0096	0.0320	0.0221	0.0003
0.0003	0.0003	0.0003	0.0003	0.0003	0.0003
0.0003	0.0003	0.0003	0.0003	0.0003	0.0003
0.0061	0.0187	0.0085	0.0003	0.0003	0.0003
0.0003	0.0179	0.0003	0.0246	0.0003	0.0003
0.0003	0.0139	0.0003	0.0003	0.0113	0.0003
0.0003	0.0003	0.0003	0.0003	0.0003	0.0003
0.0003	0.0003	0.0003	0.0003	0.0003	0.0003
0.0003	0.0003	0.0003	0.0003	0.0003	0.0003
0.0003	0.0003	0.0003	0.0003	0.0003	0.0003
0.0003	0.0003	0.0003	0.0003	0.0003	0.0003
0.0080	0.0003	0.0003	0.0003	0.0228	0.0181
0.0055	0.0049	0.0032	0.0023	0.0003	0.0041
0.0016	0.0003	0.0003	0.0021	0.0003	0.0003
0.0003	0.0003	0.0003	0.0003	0.0003	0.0003
0.0003	0.0003	0.0003	0.0003	0.0003	0.0003
0.1288	0.2484	0.2007	0.2787	0.2009	0.1617

CA 14 VULV TPPU + IL-1β 3 **90**	CA 20 VULV TPPU + IL-1β 1 **124**	CA 20 VULV TPPU + IL-1β 2 **125**	CA 20 VULV TPPU + IL-1β 3 **126**
0.0003	0.0003	0.0003	0.0003
0.0003	0.0003	0.0044	0.0003
0.0003	0.0003	0.0003	0.0003
0.0003	0.0003	0.0003	0.0003
0.5164	0.4477	0.3894	0.5137
0.0054	0.0003	0.0003	0.0003
0.0003	0.0003	0.0003	0.0003
0.0003	0.0003	0.0003	0.0003
0.0003	0.0003	0.0003	0.0003
46.1536	39.7770	31.7032	42.6901
0.0325	0.0003	0.0256	0.0353
0.0814	0.0003	0.0408	0.0003
0.0003	0.0003	0.0003	0.0003
0.0003	0.0003	0.0003	0.0003
0.0003	0.0003	0.0003	0.0003
0.0401	0.0003	0.0187	0.0304
1.3895	1.3025	0.9023	1.3645
47.2974	40.5433	30.2866	43.6918
0.0003	0.0003	0.0003	0.0003
0.0003	0.0328	0.0003	0.0003
0.0003	0.0003	0.0003	0.0003
0.0646	0.0003	0.0003	0.0237
0.0003	0.0003	0.0003	0.0003
0.0003	0.0003	0.0003	0.0003
0.0003	0.0322	0.0299	0.0451
0.0790	0.0003	0.0519	0.0653
0.0003	0.0003	0.0003	0.0916
0.0003	0.0003	0.0003	0.0003
0.0003	0.0003	0.0003	0.0003
0.0003	0.0003	0.0003	0.0003
0.0003	0.0003	0.0003	0.0057
0.0003	0.0003	0.0003	0.0003
0.0003	0.3537	0.0003	0.0003
0.0003	0.0003	0.0003	0.0003
0.0003	0.0003	0.0003	0.0003
0.0003	0.0003	0.0003	0.0003
0.0003	0.0003	0.0059	0.0053
0.0653	0.0275	0.0397	0.0548
0.0003	0.0003	0.0032	0.0003
0.0003	0.0003	0.0003	0.0003
0.0115	0.0003	0.0003	0.0003
0.0122	0.0003	0.0003	0.0003
0.0003	0.0003	0.0003	0.0003
0.0053	0.0166	0.0159	0.0088
0.0105	0.0192	0.0093	0.0048
0.0003	0.0003	0.0003	0.0003
0.0003	0.0003	0.0003	0.0003
0.0003	0.0003	0.0003	0.0003
0.0003	0.0003	0.0160	0.0003

0.0003	0.0003	0.0003	0.0003
0.0003	0.0003	0.0003	0.0003
0.0003	0.0003	0.0003	0.0003
1.5113	2.8657	1.8520	2.7104
0.0003	0.0003	0.0003	0.0003
0.0003	0.0003	0.0003	0.0003
0.0003	0.0003	0.0209	0.0003
0.6235	0.3270	0.5207	0.0003
0.0003	0.0003	0.0003	0.0003
0.0003	0.0003	0.0003	0.0003
0.0003	0.0003	0.0003	0.0003
0.0003	0.0003	0.0003	0.0003
0.1574	0.0765	0.0887	0.1000
0.0293	0.0003	0.0003	0.0162
0.1407	0.1455	0.1027	0.1307
0.0003	0.0003	0.0003	0.0003
0.1594	0.1383	0.0673	0.1382
0.0003	0.0003	0.0003	0.0003
0.0003	0.0003	0.0003	0.0003
0.0328	0.0442	0.0303	0.0434
0.0003	0.0003	0.0003	0.0003
0.0003	0.0003	0.0003	0.0003
0.0003	0.0003	0.0003	0.0003
0.0003	0.0003	0.0003	0.0003
0.0003	0.0003	0.0003	0.0003
0.0003	0.0003	0.0003	0.0003
0.0003	0.0003	0.0003	0.0003
0.0003	0.0003	0.0003	0.0003
0.0003	0.0003	0.0003	0.0003
0.0003	0.0003	0.0003	0.0003
0.0003	0.0003	0.0003	0.0003
0.0156	0.0003	0.0003	0.0003
0.0003	0.0003	0.0003	0.0003
0.0003	0.0003	0.0003	0.0003
0.0003	0.0045	0.0003	0.0003
0.1325	0.1182	0.0714	0.1030
0.0003	0.0003	0.0003	0.0003
0.0003	0.2426	0.1117	0.1201
0.0003	0.0948	0.0003	0.0003
0.6240	0.0003	0.8495	0.0003
0.0003	0.0003	0.0003	0.0003
0.0267	0.0244	0.0318	0.0302
7.7136	7.7758	5.7375	6.5010
1.3849	1.7040	1.3477	1.3899
0.0003	0.0003	0.0003	0.0003
0.4619	0.6660	0.5054	0.4893
0.0642	0.1001	0.0907	0.1219
0.0003	0.0003	0.0003	0.0003
0.0003	0.0003	0.0003	0.0003
0.0150	0.0003	0.0047	0.0003
0.0003	0.0003	0.0003	0.0003
0.0003	0.0003	0.0003	0.0003
0.0644	0.0466	0.0446	0.0455

0.0151	0.0003	0.0003	0.0003
0.0003	0.0003	0.0149	0.0003
0.0003	0.0003	0.0003	0.0003
0.0003	0.0003	0.0003	0.0026
0.0003	0.0162	0.0003	0.0086
0.0201	0.0160	0.0117	0.0197
0.0003	0.0003	0.0003	0.0003
0.0003	0.0003	0.0065	0.0003
0.2578	0.0003	0.0003	0.0003
0.0003	0.0003	0.0003	0.0003
0.0003	0.0003	0.0003	0.0003
0.0246	0.0105	0.0168	0.0235
0.0003	0.0003	0.0003	0.0003
0.0003	0.0003	0.0003	0.0003
0.0003	0.0003	0.0003	0.0003
0.0003	0.0227	0.0003	0.0437
0.0003	0.0003	0.0003	0.0083
0.0003	0.0003	0.0003	0.0003
0.0003	0.0003	0.0003	0.0003
0.5671	0.4864	0.3606	0.5299
0.0003	0.0003	0.0164	0.0003
0.0281	0.0003	0.0003	0.0003
0.0003	0.0350	0.0003	0.0003
0.0003	0.0003	0.0003	0.0003
0.0003	0.0003	0.0003	0.0003
0.0003	0.0003	0.0003	0.0003
0.0003	0.0003	0.0003	0.0003
0.0003	0.0603	0.0003	0.0291
0.0155	0.0051	0.0003	0.0003
0.0003	0.0003	0.0003	0.0003
0.0003	0.0003	0.0003	0.0003
0.0110	0.0003	0.0003	0.0003
0.0003	0.0003	0.0003	0.0003
0.0108	0.0003	0.0003	0.0234
0.0003	0.0003	0.0003	0.0003
0.0003	0.0003	0.0003	0.0003
0.0003	0.0003	0.0003	0.0003
0.0003	0.0003	0.0003	0.0003
0.0003	0.0003	0.0003	0.0003
0.0433	0.0003	0.0052	0.0161
0.0066	0.0030	0.0022	0.0003
0.0018	0.0003	0.0025	0.0020
0.0003	0.0003	0.0003	0.0003
0.0003	0.0003	0.0003	0.0003
0.1994	0.1169	0.0825	0.1598

Table S3. Full 17-plex Luminex Dataset

Cytokine concentrations are in pg/mL. Cytokines highlighted in red were mostly below the limit of the detection and therefore not plotted on a graph in Figure S3.

			1. G-CSF (57)	2. GM-CSF (34)	3. IFN-y (21)	4. IL-1B (39)	5. IL-2 (38)	6. IL-4 (52)	7. IL-5 (33)	8. IL-6 (19)	9. IL-7 (74)	10. IL-8 (54)	11. IL-10 (56)	12. IL-12 (75)	13. IL-13 (51)	14. IL-17 (76)	15. MCP-1 (53)	16. MIP-1B (18)	17. TNF-a (36)
**Type**	**Well**	**Description**	**Conc in Range**	**Conc in Range**	**Conc in Range**	**Conc in Range**	**Conc in Range**	**Conc in Range**	**Conc in Range**	**Conc in Range**	**Conc in Range**	**Conc in Range**	**Conc in Range**	**Conc in Range**	**Conc in Range**	**Conc in Range**	**Conc in Range**	**Conc in Range**	**Conc in Range**
B	A12	BLANK																	
B	B12	BLANK																	
S1	A1	STANDARD	143028.39	6463.54		5237.19	43431.32	2709.6	163174.97	6924.87	59374.74	7520.19	OOR >	34196.79	4575.04	60308.36	4320.1	6077.98	OOR >
S1	A2	STANDARD	OOR >	OOR >		OOR >	OOR >	OOR >	OOR >	OOR >	OOR >	OOR >	29306.56	OOR >	OOR >	OOR >	OOR >	OOR >	73014.65
S2	B1	STANDARD	36714.06	1696.26	8144.44	2554.27	9965.91	811.16	45465.87	1693.23	14371.6	3062.71	6542.6	8547.51	1100.21	14728.71	2154	1628.63	36101.02
S2	B2	STANDARD	36073.05	1800.38	OOR >	2033.9	11243.68	909.19	45331.9	1819.51	14649.99	2854.74	7734.55	9111.76	1158.64	16323.82	2547.34	1852.31	35583.93
S3	C1	STANDARD	9074.82	426.82	2109.97	535.54	2807.04	206.49	10495.31	430.76	3829.12	750.41	1848.47	2229.36	295.97	3670.92	536.57	399.9	8502.05
S3	C2	STANDARD	9711.62	443.52	2093.73	511.18	3097.69	227.05	11134.13	437.98	3844.83	729.78	1913.25	2176.48	303.24	3804.93	534.87	417.3	9582.68
S4	D1	STANDARD	2276.76	113.73	535.4	136.85	720.34	53.89	2748.75	114.39	930.74	177.73	475.22	556.91	73.98	946.76	140.74	105.56	2279.09
S4	D2	STANDARD	2287.09	107.65	459.26	141.19	722.84	54.86	2748.75	114.14	875.42	190.11	436.32	548.01	72.08	981.61	142.84	109.06	2222.18
S5	E1	STANDARD	555.01	26.45	127.07	33.05	166.71	12.76	713.91	24.27	229.44	48.41	109.85	135.77	16.96	240.23	32.83	27.33	510.9
S5	E2	STANDARD	551.15	27.09	138.38	34.32	182.59	13.43	685.16	26.89	221.83	45	114.51	141.07	16.96	240.23	35.2	26.1	536.72
S6	F1	STANDARD	153.05	8.05	32.58	8.55	42.4	3.44	169.55	7.61	60.89	8.23	27.92	35.59	5.31	60.74	9.14	6.45	
S6	F2	STANDARD		5.96	30.56	7.99	47.02	3.55	163.19	7.8	70.14	14.71	31.7	31.53	4.44	55.56	8.14	6.37	159.81
S7	G1	STANDARD	OOR <	2.04	8.06	2.45	11.57	0.98	43.31	2.07	OOR <	2.02	7.48	11.47	1.13	14.96	2.26	1.81	30.9
S7	G2	STANDARD	36.68	1.29	8.06	1.79	11.42	0.71	43.31	0.89	15.02	3.82	6.26	7.55	1.13	15.48	1.95	1.47	36.05
S8	H1	STANDARD		OOR <	2.53	OOR <	3.41	OOR <	12.23	0.73		OOR <	OOR <	3.69	OOR <	OOR <	0.6	0.67	10.3
S8	H2	STANDARD		0.45	OOR <	0.62	OOR <	0.28	OOR <	OOR <		0.95	2.5	OOR <	0.32	3.92	OOR <	OOR <	OOR <
C1	C12	CONTROL	2105.81	57.86	23.13	27.89	339.53	6.54	1475.88	50.22	159.28	26.11	139.11	216.98	37.43	298.6	42.61	31.24	867.41
C1	D12	CONTROL	1854.1	62.04	27.14	28.53	345.78	7.04	1529.73	68.89	179.18	26.43	146.76	232.06	39.73	314.83	46.46	32.01	748.55
X1	A3	13 VEST IL-1B	115514.28	483.51	466.62	61.68	OOR <	136.26	8934.18	79823.52	OOR <	80991.34	217.99	754.7	OOR <	OOR <	41871.38	832.01	2059.94
X1	B3	13 VEST IL-1B	100843.49	497.2	406.07	61.68	OOR <	123.71	8934.18	75562.95	OOR <	128333.72	OOR <	368.61	31.94	OOR <	38563.44	766.35	2574.92
X2	A4	13 VEST GSK	7120.75	39.07	42.63	5.32	OOR <	9.8	778	5395.62	OOR <	7957.45	OOR <	75.47	3.19	OOR <	6816.95	52.98	257.49
X2	B4	13 VEST GSK	6712.14	37.18	48.67	6.17	OOR <	10.45	704.67	5567.09	OOR <	10735.38	OOR <	36.86	3.78	OOR <	6642.05	60.49	360.52
X3	A5	13 VEST EC5026	7179.2	33.35	44.65	OOR <	OOR <	11.1	725.61	6331.78	OOR <	8318.47	OOR <	27.36	OOR <	OOR <	8179.85	65.29	205.99
X3	B5	13 VEST EC5026	6984.44	31.41	39.59	OOR <	OOR <	11.1	809.46	6055.8	OOR <	7477.85	OOR <	95.02	OOR <	OOR <	8343.77	70.53	205.99
X4	A6	14 VEST IL-1B	98078.27	289.67	406.07	OOR <	OOR <	123.71	8304.4	73030.33	OOR <	111883.12	OOR <	560.67	OOR <	OOR <	52952.12	713.44	2059.94
X4	B6	14 VEST IL-1B	98473.09	418.85	406.07	53.23	OOR <	98.02	7884.89	64956.22	OOR <	122872.34	OOR <	368.61	26.12	OOR <	47548.28	636.88	1545.1
X5	A7	14 VEST GSK	15599.41	27.48	51.69	6.17	OOR <	16.09	725.61	8630.68	OOR <	15041.95	OOR <	36.86	OOR <	OOR <	16357.27	88.99	257.49
X5	B7	14 VEST GSK	12388.02	25.49	58.7	11.2	OOR <	14.86	620.97	8259.12	OOR <	10421.29	25.02	36.86	OOR <	OOR <	15854.59	82.38	205.99
X6	A8	14 VEST EC5026	12447.89	20.39	60.7	7.01	OOR <	14.25	704.67	9105.41	OOR <	13876.91	OOR <	36.86	OOR <	OOR <	16489.74	88.99	154.51
X6	B8	14 VEST EC5026	13908.75	19.34	61.69	6.17	OOR <	14.25	746.56	9052.65	OOR <	15944.51	OOR <	OOR <	OOR <	OOR <	15340.91	74.19	154.51
X7	A9	20 VEST IL-1B	166915.34	328.69	416.18	78.52	OOR <	196.84	8619.22	135854.85	OOR <	158835.81	OOR <	754.7	OOR <	440.81	54558.18	998.57	2574.92
X7	B9	20 VEST IL-1B	112334.6	294.59	426.29	OOR <	OOR <	172.99	7465.64	100997.21	OOR <	143702.41	OOR <	179.52	OOR <	OOR <	47108.88	939.9	1545.1
X8	A10	20 VEST GSK	7452.2	17.23	46.66	OOR <	OOR <	12.37	558.28	8196.46	OOR <	8376.82	OOR <	46.44	OOR <	39.2	11911.25	68.51	205.99
X8	B10	20 VEST GSK	6420.86	16.16	37.57	OOR <	OOR <	12.37	547.84	8231.69	OOR <	8734.26	OOR <	OOR <	OOR <	OOR <	12508.96	73.37	205.99
X9	A11	20 VEST EC5026	10955.58	16.69	56.7	8.69	OOR <	13.63	662.8	11067.36	OOR <	9458.91	OOR <	OOR <	OOR <	OOR <	13847.3	77.86	154.51
X9	B11	20 VEST EC5026	8116.87	21.42	48.67	6.17	OOR <	14.86	641.88	11001.77	OOR <	10256.04	OOR <	36.86	OOR <	OOR <	13103.48	87.33	128.77
X10	C3	13 VULV IL-1B	113327.81	662.36	456.55	OOR <	OOR <	136.26	8514.27	78163.43	OOR <	133069.13	OOR <	950.19	31.94	OOR <	51375.77	685.13	2574.92
X10	D3	13 VULV IL-1B	132273.49	573.88	506.84	86.91	OOR <	154.79	10721.6	87064.93	OOR <	187328.66	OOR <	754.7	OOR <	OOR <	55360.29	910.71	2574.92
X11	C4	13 VULV GSK	14913.88	43.28	55.7	6.17	OOR <	14.25	914.42	6875.16	OOR <	19492.98	OOR <	36.86	OOR <	39.2	12301.3	69.72	360.52
X11	D4	13 VULV GSK	9788.09	49.26	56.7	OOR <	OOR <	13.63	861.92	5234.22	OOR <	14788.83	OOR <	95.02	OOR <	OOR <	10779.78	60.49	103.04
X12	C5	13 VULV EC5026	13627.96	43.75	45.66	7.85	OOR <	14.25	736.09	6263.76	OOR <	17161.68	OOR <	56.07	2.61	44.08	11027.29	69.32	103.04
X12	D5	13 VULV EC5026	15478.32	43.28	58.7	OOR <	OOR <	15.48	924.93	7123.39	OOR <	19402.66	OOR <	36.86	4.36	OOR <	13659.65	75.82	205.99
X13	C6	14 VULV IL-1B	76865.3	519.91	446.47	53.23	OOR <	123.71	8724.19	71178.05	OOR <	112643.36	OOR <	560.67	31.94	OOR <	41664.65	596.97	1545.1
X13	D6	14 VULV IL-1B	67121.36	537.98	385.81	OOR <	OOR <	98.02	7989.74	51818.64	OOR <	101705.85	OOR <	273.58	OOR <	OOR <	37120.14	589.02	1545.1
X14	C7	14 VULV GSK	8665.92	31.41	77.62	7.85	OOR <	13.63	704.67	7359.48	OOR <	15581.88	OOR <	56.07	OOR <	OOR <	13322.59	60.49	103.04
X14	D7	14 VULV GSK	9965.8	32.39	53.69	5.32	OOR <	13.63	778	7265.48	OOR <	17541.2	OOR <	46.44	OOR <	OOR <	15187.94	67.71	205.99
X15	C8	14 VULV EC5026	7706.07	27.48	60.7	10.37	OOR <	13.63	736.09	8075.54	OOR <	16381.52	18.55	75.47	3.19	OOR <	13895.62	77.86	103.04
X15	D8	14 VULV EC5026	8156.04	30.44	48.67	OOR <	OOR <	9.8	662.8	6761.29	OOR <	14215.7	OOR <	OOR <	3.19	OOR <	13993	60.89	OOR <
X16	C9	20 VULV IL-1B	75693.31	442.09	526.91	OOR <	OOR <	172.99	7256.13	121470.5	OOR <	136186.05	OOR <	560.67	OOR <	OOR <	61300.18	935.72	2059.94
X16	D9	20 VULV IL-1B	78624.63	474.35	466.62	86.91	OOR <	160.89	8724.19	116691.17	OOR <	136766.11	OOR <	464.35	OOR <	OOR <	58946.85	1028.06	1030.42
X17	C10	20 VULV GSK	6207.57	27.98	55.7	OOR <	OOR <	13.63	620.97	8895.06	OOR <	14957.19	OOR <	OOR <	2.61	39.2	15621.39	93.57	77.32
X17	D10	20 VULV GSK	7315.65	35.27	60.7	7.85	OOR <	14.86	652.34	9362.67	OOR <	15334.91	OOR <	36.86	OOR <	39.2	17433.17	92.32	257.49
X18	C11	20 VULV EC5026	7686.53	29.46	58.7	7.01	OOR <	18.5	704.67	12771.8	OOR <	14465.33	OOR <	56.07	OOR <	OOR <	24378.6	115.12	257.49
X18	D11	20 VULV EC5026	8941	34.31	60.7	9.53	OOR <	18.5	746.56	12288.31	OOR <	56021.19	OOR <	OOR <	4.36	39.2	20803.17	104.07	154.51
X19	E3	4 VEST IL-1B	224992.95	446.72	626.92	95.29	OOR <	243.36	10195.44	122883.1	OOR <	207129.94	OOR <	950.19	OOR <	440.81	87448.84	965	2832.44
X19	F3	4 VEST IL-1B	229535.15	347.94	626.92	112.02	OOR <	208.61	9774.78	127161.48	OOR <	150285.45	OOR <	754.7	43.56	367.75	85512.11	1040.72	3089.99
X20	E4	4 VEST GSK	9669.69	30.44	60.7	6.17	OOR <	12.37	809.46	5039.11	OOR <	7793.11	OOR <	36.86	3.19	OOR <	17305.9	54.16	205.99
X20	F4	4 VEST GSK	8901.68	32.39	68.67	6.17	OOR <	12.37	851.43	5047.47	OOR <	12229.69	OOR <	56.07	OOR <	OOR <	16099.63	50.63	154.51
X21	E5	4 VEST EC5026	8273.59	26.49	64.69	7.85	OOR <	11.1	851.43	5584.33	OOR <	9105.64	OOR <	56.07	OOR <	44.08	18097.38	62.09	309
X21	F5	4 VEST EC5026	9926.29	23.47	74.64	9.53	OOR <	14.86	798.97	6468.52	120.37	7888.53	OOR <	56.07	OOR <	OOR <	19568.81	60.49	257.49
X22	E6	10 VEST IL-1B	95710.78	451.34	456.55	86.91	OOR <	220.28	8514.27	160978.41	OOR <	104074.61	OOR <	368.61	31.94	440.81	49929.13	1049.17	2574.92
X22	F6	10 VEST IL-1B	94725.08	376.53	536.93	86.91	OOR <	196.84	9144.24	143522.97	1502.17	142328.11	OOR <	560.67	43.56	392.05	41252.2	1159.71	2059.94
X23	E7	10 VEST GSK	11491.78	30.44	62.69	12.04	OOR <	32.19	914.42	22408.14	OOR <	14417.72	OOR <	65.75	OOR <	46.53	13121.3	141.48	334.76
X23	F7	10 VEST GSK	13327.45	30.44	62.69	16.2	OOR <	29.98	987.99	21799.68	OOR <	65984.84	OOR <	36.86	OOR <	53.89	12332.63	172	309
X24	E8	10 VEST EC5026	10796.93	29.95	82.58	12.87	OOR <	35.47	1040.59	27199	OOR <	31794.73	OOR <	36.86	3.19	44.08	18045.28	183.66	360.52
X24	F8	10 VEST EC5026	13207.33	27.48	84.57	14.54	OOR <	32.19	893.42	28275.34	OOR <	24420.58	18.55	36.86	5.52	51.43	15217.59	159.53	309
X25	E9	23 VEST IL-1B	39385.79	188.17	506.84	78.52	OOR <	172.99	7675.23	132442.44	OOR <	106510.73	OOR <	754.7	26.12	OOR <	70868.13	1006.99	1545.1
X25	F9	23 VEST IL-1B	55693.95	333.52	426.29	128.71	OOR <	231.86	8094.61	176493.72	OOR <	351647.98	OOR <	368.61	OOR <	440.81	78326.44	1172.55	2059.94
X26	E10	23 VEST GSK	1623.02	16.16	48.67	OOR <	OOR <	11.1	537.4	7019.3	OOR <	8208.46	OOR <	36.86	OOR <	OOR <	12486.73	59.3	OOR <
X26	F10	23 VEST GSK	1382.79	26.99	44.65	OOR <	OOR <	12.37	600.06	7093.6	OOR <	6836.12	OOR <	36.86	4.36	OOR <	11550.88	68.51	128.77
X27	E11	23 VEST EC5026	2088	20.39	54.69	5.32	OOR <	13.63	746.56	9072.93	OOR <	8690.35	OOR <	OOR <	OOR <	OOR <	14348.77	70.53	103.04

X27	F11	23 VEST EC5026	1976.08	18.29	52.69	OOR <	OOR <	12.37	537.4	7992.04	OOR <	7225.73	OOR <	OOR <	OOR <	OOR <	12256.53	69.32	103.04
X28	G3	4 VULV IL-1B	124279.31	428.17	586.97	78.52	OOR <	148.65	9564.54	98265.95	OOR <	110336.54	OOR <	179.52	OOR <	OOR <	54915.41	923.21	1030.42
X28	H3	4 VULV IL-1B	111540.33	437.46	466.62	95.29	OOR <	148.65	8934.18	79011.26	OOR <	103109.52	OOR <	950.19	31.94	OOR <	50583.21	701.29	2059.94
X29	G4	4 VULV GSK	20278.36	43.28	76.63	11.2	OOR <	25.48	1040.59	13709.63	OOR <	27526.45	OOR <	75.47	OOR <	44.08	20385.93	103.23	205.99
X29	H4	4 VULV GSK	22850.23	36.23	60.7	13.7	OOR <	23.19	1009.03	14898.53	OOR <	24204.63	OOR <	36.86	4.36	39.2	18529.31	96.5	205.99
X30	G5	4 VULV EC5026	22293.03	38.13	83.58	12.04	OOR <	26.61	998.51	16327.24	OOR <	19402.66	OOR <	46.44	3.78	48.98	21440.39	123.69	257.49
X30	H5	4 VULV EC5026	23056.83	40.48	82.58	16.2	OOR <	26.05	1019.54	18108.09	OOR <	21878.89	OOR <	65.75	3.19	44.08	23017.26	141.92	309
X31	G6	10 VULV IL-1B	41675.63	455.95	365.5	61.68	OOR <	71.29	7884.89	38332.55	OOR <	80749.34	OOR <	852.28	OOR <	OOR <	36529.05	402.01	3089.99
X31	H6	10 VULV IL-1B	42439.89	437.46	324.75	OOR <	OOR <	84.81	8094.61	39500.68	OOR <	77515.3	OOR <	464.35	31.94	OOR <	42066.62	405.82	1545.1
X32	G7	10 VULV GSK	3805.21	29.46	56.7	6.17	OOR <	8.48	631.42	3915.71	OOR <	9903.24	OOR <	OOR <	OOR <	OOR <	10563.5	46.35	205.99
X32	H7	10 VULV GSK	4684.35	32.87	45.66	6.17	OOR <	11.1	620.97	3844.12	OOR <	11601.28	OOR <	46.44	OOR <	OOR <	10024	45.58	103.04
X33	G8	10 VULV EC5026	6130.08	28.47	48.67	OOR <	OOR <	11.1	620.97	6144.4	OOR <	20396.35	OOR <	OOR <	OOR <	OOR <	11555.89	55.73	103.04
X33	H8	10 VULV EC5026	5743.19	30.93	58.7	7.85	OOR <	9.8	641.88	6367.68	OOR <	27855.78	OOR <	36.86	OOR <	OOR <	13776.89	72.16	257.49
X34	G9	23 VULV IL-1B	119096.3	404.81	566.97	78.52	OOR <	254.78	7884.89	173395.75	OOR <	177261.06	OOR <	464.35	26.12	440.81	102821.36	1297.39	2832.44
X34	H9	23 VULV IL-1B	105195.59	414.18	486.74	86.91	OOR <	214.45	7884.89	146021.47	OOR <	158135.21	OOR <	368.61	OOR <	OOR <	90833.07	1310.38	OOR <
X35	G10	23 VULV GSK	6926.05	23.47	56.7	6.17	OOR <	16.09	662.8	9922.91	OOR <	15280.48	OOR <	OOR <	OOR <	OOR <	16823.48	101.96	205.99
X35	H10	23 VULV GSK	7803.8	21.42	70.66	7.85	OOR <	18.5	704.67	11379.92	OOR <	11246.21	OOR <	36.86	OOR <	OOR <	24185.25	92.32	OOR <
X36	G11	23 VULV EC5026	10995.26	24.48	68.67	11.2	OOR <	22.03	746.56	16126.11	OOR <	18776.44	OOR <	56.07	OOR <	OOR <	24688.38	126.71	257.49
X36	H11	23 VULV EC5026	7023.37	19.86	60.7	7.85	OOR <	17.3	830.44	12869.43	OOR <	11656.83	OOR <	36.86	OOR <	OOR <	19661.04	99.02	154.51

*** = Value not available;   --- = Designated as an outlier

*Value = Value extrapolated beyond standard range

OOR = Out of Range;   OOR> = Out of Range Above;   OOR< = Out of Range Below

Exp Conc = Expected Concentration;   Obs Conc = Observed Concentration
